# Noncanonical Surface Glycoconjugates from *Bacteroides stercoris* DSM 19555 Reprogram Innate Immune Responses

**DOI:** 10.1021/jacsau.6c00878

**Published:** 2026-07-25

**Authors:** Luca De Simone Carone, Stefania De Chiara, Marcello Mercogliano, Francesca Olmeo, Valentina Mazziotti, Alba Silipo, Freda M. Farquharson, Petra Louis, Antonio Molinaro, Flaviana Di Lorenzo

**Affiliations:** † Department of Chemical Sciences, 9307 University of Naples Federico II, Via Cinthia, 4, Naples 80126, Italy; ‡ CEINGE-Biotecnologie Avanzate Franco Salvatore, Via Gaetano Salvatore 486, Naples 80145, Italy; § The Rowett Institute, University of Aberdeen, Foresterhill, Aberdeen AB25 2ZD, U.K.

**Keywords:** gut microbiota, lipopolysaccharide (LPS), capsular polysaccharide (CPS), bacteroides, immunomodulation

## Abstract

Deciphering the role(s) of surface glycoconjugates in gut microbiota-immune interactions is essential for improving human health. This study shows that *Bacteroides stercoris* DSM 19555 produces two previously undescribed surface glycoconjugates: an atypical rough-type lipopolysaccharide (R-LPS) and a positively charged capsular polysaccharide (CPS). Structural analysis revealed an R-LPS featuring a 2-aminoethyl phosphate-substituted 2-keto-3-deoxy-d-*manno*-octulosonic acid (Kdo) bearing a phosphodiester-linked hexose, a fucose-lacking core oligosaccharide, and a highly heterogeneous *mono*-phosphorylated, hypoacylated lipid A. In parallel, a branched, *galacto*-configured, and amino sugar-rich cationic CPS is also identified. Both glycoconjugates promote anti-inflammatory IL-10 production while limiting classical pro-inflammatory outputs, with CPS exhibiting the strongest immunomodulatory activity. In an inflamed epithelial-immune coculture model, both glycoconjugates shape cytokine responses toward an IL-10-associated, low-inflammatory profile. These findings uncover a structurally and functionally distinct glycan repertoire in *B. stercoris* DSM 19555 and support the emerging view that gut Bacteroidales surface glycans behave as active modulators of innate immune responses rather than as simple inflammatory triggers.

## Graphical abstract

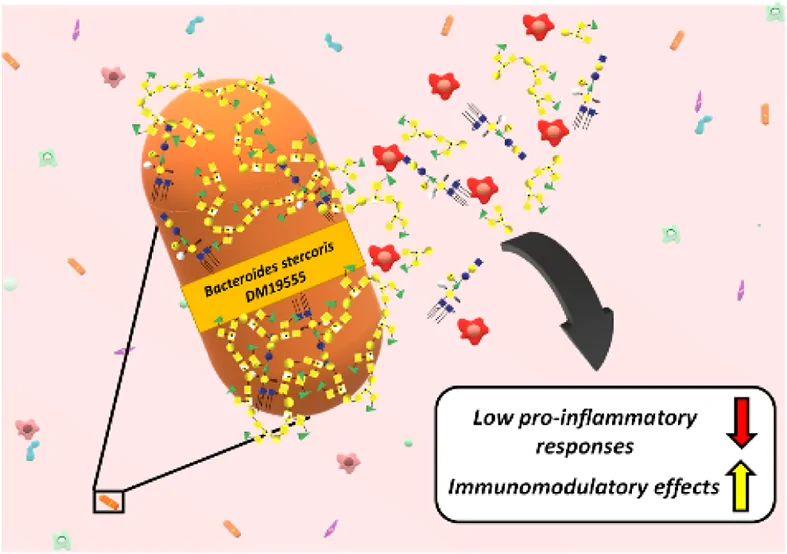

## Introduction

The human gut microbiota is increasingly recognized as a major regulator of host physiology, contributing to metabolism, immune education, and maintenance of epithelial homeostasis.^1^ Yet, the molecular basis of host-microbe communication remains only partially understood. A growing body of evidence indicates that glycans are central mediators of this bidirectional dialog. Present on both host tissues and microbial surfaces, glycans regulate bacterial colonization, nutrient utilization, receptor engagement, and immune cell activation, thereby contributing to microbial persistence and immune homeostasis.^2,3^ Among the most abundant and immunologically relevant microbial components are surface glycoconjugates, which constitute the primary chemical interface between bacteria and the host’s immune system. In Gram-negative bacteria, lipopolysaccharides (LPSs) and capsular polysaccharides (CPSs) are key determinants of bacterial fitness and niche adaptation.^4,5^ Despite their central role, the structural diversity and immunological impact of these glycoconjugates remain largely unexplored for the majority of gut commensals. This represents a major knowledge gap, as subtle structural variations in LPSs and CPSs are known to profoundly influence host immune responses, shifting signaling outcomes from strongly proinflammatory to weakly inflammatory or even immunomodulatory states. Intriguingly, recent studies have begun to challenge the long-standing paradigm of LPSs as universally proinflammatory molecules. In particular, several LPSs from gut commensals display unusual chemical architectures that correlate with attenuated or regulatory immune responses.^6–14^ These findings strongly underline that LPSs are not inherently pathogenic signals but rather complex molecules capable of promoting immune homeostasis. However, the molecular principles underlying these effects have only started to emerge. A similar conceptual shift is occurring for the CPSs. Historically regarded as virulence factors and vaccine targets in pathogens, CPSs are now recognized as key modulators of host-microbe interactions in commensal bacteria, with evidence linking them to both protective and disease-associated effects.^15,16^ Nevertheless, the chemical structures, biosynthetic pathways, and immunological functions of CPSs from gut commensals remain largely undefined.

*Bacteroides stercoris* is a prevalent member of the human gut microbiota whose association with host physiology remains ambiguous. It has been linked to metabolically favorable states, including enrichment in healthy individuals and in fiber-rich dietary contexts,^17^ showing antiobesity activity in experimental murine models,^18^ but has also been reported in inflammatory and metabolic disorders, including Crohn’s disease^19^ and diabetic nephropathy.^20^ However, the functional interpretation of these observations remains largely speculative, as they are almost exclusively derived from sequencing-based studies. To date, no structural information is available on the major (potentially immunostimulatory) glycoconjugates of *B. stercoris*, and their biosynthetic pathways and immunological properties remain completely unexplored. This lack of molecular insight represents a critical gap, preventing a mechanistic understanding of how this prevalent commensal interacts with the host immune system.

Here, we provide the first structural and functional characterization of both the LPS and the CPS from *B. stercoris* DSM 19555. We show that this bacterium produces previously unreported surface glycoconjugates, including an atypical R-type LPS (an LPS devoid of the O-antigen polysaccharide or R-LPS) and a positively charged CPS, and identify the genomic loci likely responsible for their biosynthesis. Functional analyses across multiple cell models reveal that both glycoconjugates display the ability to promote regulatory immune outputs, including anti-inflammatory IL-10 production, while limiting pro-inflammatory responses. Together, these findings reveal a structurally distinct glycan portfolio in *B. stercoris* DSM 19555 that biases innate immune responses toward regulation, providing a molecular framework to rationalize its context-dependent associations with host health and disease.

## Results

### *B. stercoris* Produces Surface Glycoconjugates with Peculiar Chemical Structures

To elucidate the structure of R-LPS and CPS, we employed an integrated analytical approach combining chemical analyses, high-resolution mass spectrometry (MS), and nuclear magnetic resonance (NMR) spectroscopy. R-LPS and CPS were isolated from bacterial cells, extensively purified, and subjected to compositional analysis. The rough nature of the R-LPS was confirmed by SDS-PAGE followed by silver staining. Subsequently, two aliquots of R-LPS were processed in parallel by complete deacylation and mild acid hydrolysis to separate the lipid A and core oligosaccharide (core OS) domains, enabling independent structural analyses of the glycolipid and carbohydrate portions. The resulting fractions, together with CPS, were further purified by sequential size-exclusion chromatography and then characterized by 1D and 2D NMR spectroscopy (see Supporting Information for full NMR structural assignment). NMR analysis revealed that the first sugar of the core OS, i.e., the 2-keto-3-deoxy-d-*manno*-octulosonic acid (Kdo) residue, is substituted at position O-7 by a 2-aminoethyl phosphate (*P*EtN) and at position O-4 by a phosphate group while connecting the lipid A glucosamine-disaccharide backbone to a branched core OS composed of α-l-rhamnopyranose (α-Rha*p*), β-d-galactofuranose (β-Gal*f*), β-d-glucopyranose (β-Glc*p*), α-d-galactopyranose (α-Gal*p*), and 2-acetamido-2-deoxy-β-d-glucopyranose (β-Glc*p*NAc) (Table S1, and Figure 1a,b,d, S1–S4). Notably, negative-ion ESI-MS analysis of the intact R-LPS revealed the occurrence of an additional hexose unit, likely glucose based on compositional analysis, not detected by NMR, possibly due to its partial loss during the deacylation procedure (Figure 1c, Table S2, calculated mass 3109.640 Da). In fact, ESI-MS/MS fragmentation confirmed a *P*EtN-Kdo-*P*-Hex motif, thus enabling positioning of this hexose as a phosphodiester-linked substituent at position O-4 of Kdo (see Supporting Information for full MS and MS/MS structural description, Table S2 and Figure 1c, S5–S6), representing a previously unreported feature among *Bacteroides* LPSs described to date. Moreover, MS analysis conducted on the bacterial pellet, intact R-LPS, and the isolated lipid A fraction (Figure 1d, Table S3 and Figure S7), supported by fatty acid analysis (Table S3), revealed a highly heterogeneous *mono*-phosphorylated and tetra- and penta-acylated lipid A, consistent with *Bacteroides*-type structures.^6,11^

**Figure 1:**
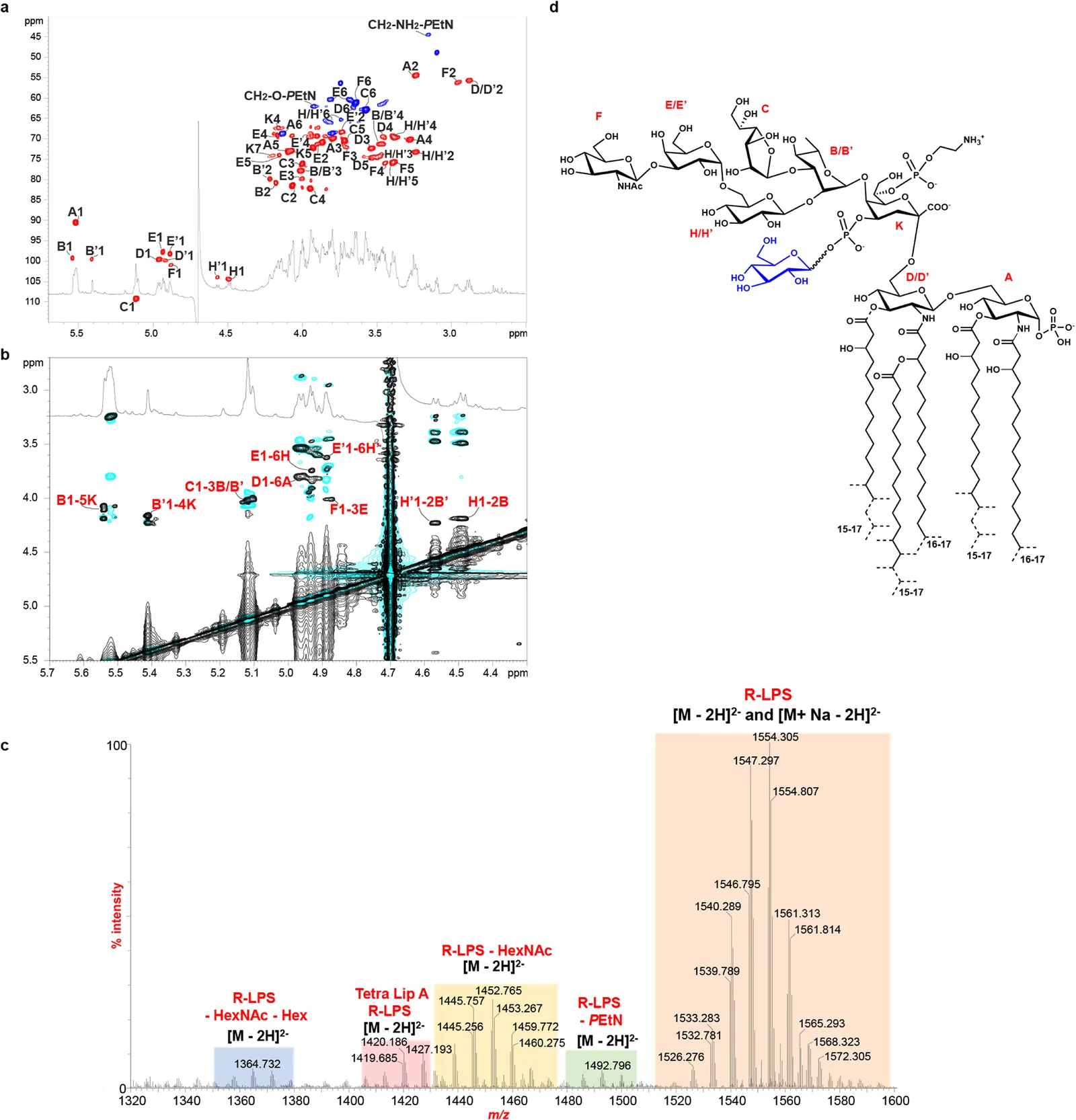
Structural characterization of *B. stercoris*R-LPS. (**a**) (600 MHz, 298 K, D_2_O, Table S1) Superimposed ^1^H and ^1^H, ^13^C HSQC (red and blue) spectra of the core OS obtained after full deacylation of R-LPS. Main heteronuclear one-bond correlations are reported. (**b**) Zoom of the overlapped ^1^H, ROESY (black), and TOCSY (light blue) spectra of the core OS after full deacylation of R-LPS. Key *inter*-residue NOE correlations involving sugar units are indicated; residue annotations correspond to those reported in Table S1. (**c**) Enlargement of the negative-ion ESI mass spectrum of *B. stercoris* R-LPS (see also Table S2). (**d**) Proposed structure of the R-LPS derived from the combined compositional, NMR, and MS analyses, with residue labeling as defined in Table S1. The additional hexose residue detected by ESI-MS and shown in blue is tentatively assigned as glucose, based on compositional analyses, although its anomeric configuration remains to be defined.

In parallel, CPS was characterized as a branched pentasaccharide enriched in *galacto*-configured residues and amino sugars. The repeating unit is made up of a 4,6-disubstituted 2-amino-2-deoxy-β-d-galactopyranose (β-Gal*p*N) residue, which carries a terminal β-Gal*p* at O-4 and a 2-acetamido-2-deoxy-β-d-galactopyranose (β-Gal*p*NAc) at O-6. The latter is further extended at O-3 by an α-Rha*p* residue, which is in turn substituted at O-4 by a β-Gal*p*NAc unit, itself further substituted at O-4 by β-Gal*p*N residue (see Supporting Information, Table S4 and Figures S1, S8–S9). Strikingly, the coexistence of free amino groups and *N*-acetylated residues confers an overall positive charge to the polymer, a feature that further distinguishes this CPS from previously described *Bacteroides* polysaccharides. Together, these data demonstrate that *B. stercoris* produces two structurally distinct and previously unreported glycoconjugates: an R-LPS featuring an atypical core OS with *P*EtN decoration and phosphodiester-linked hexose in its innermost part and a novel positively charged CPS (Figure 1d and Figure 2c, respectively).

**Figure 2:**
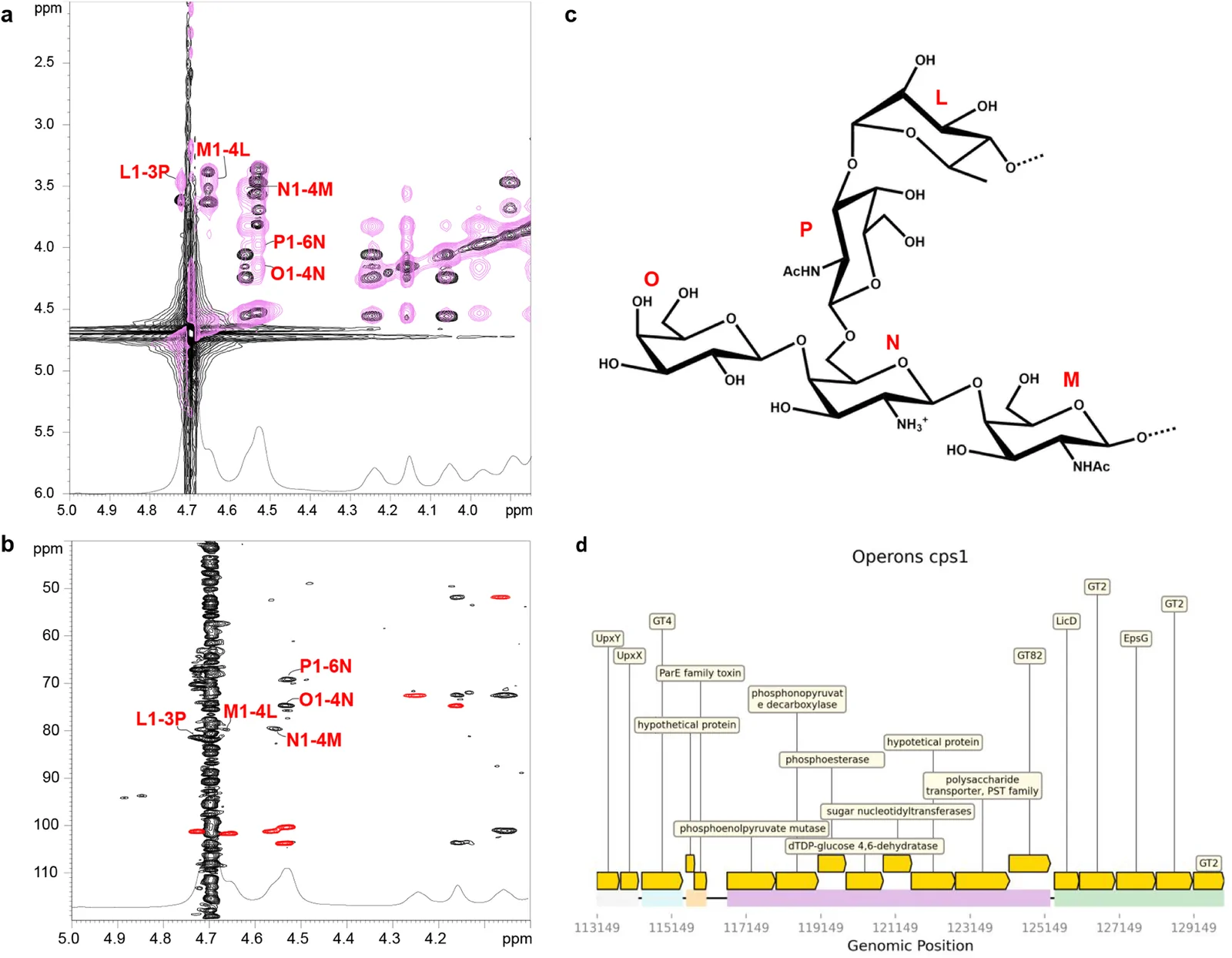
Structural characterization of *B. stercoris*CPS. (**a**) (600 MHz, 298 K, D_2_O, Table S4) Superimposed ^1^H, TOCSY (black) and NOESY (pink) spectra of the CPS. Main *inter*-residue NOE correlations are indicated. (**b**) Zoom of the overlapped ^1^H, ^1^H, ^13^C HSQC (red) and ^1^H, ^13^C HMBC (black) spectra of the CPS with key *inter*-residue long-range correlations highlighted. (**c**) Proposed structure of the repeating unit building up *B. stercoris* CPS. Residues are labeled as defined in Table S4. (**d**) Genomic organization of the putative CPS biosynthetic locus in *B. stercoris* DSM 19555. Arrows represent predicted protein-coding genes oriented in the direction of transcription, with functional annotations indicated above. Distinct background colors denote predicted operons, and genomic coordinates (bp) are shown below.

The unusual structural features uncovered by NMR and MS were mirrored at the genomic level (see Supporting Information for full description, Figures S10–S13 and Table S5). *In silico* analysis of the *B. stercoris* DSM 19555 genome identified a complete set of lipid A biosynthetic genes consistent with a penta-acylated backbone, including homologs of the Raetz pathway enzymes and lipid A-modifying enzymes (Table S5). A discrete ∼12 kb biosynthetic locus (BLV03_RS02290-BLV03_RS02340) encoded a conserved inner core module together with a highly divergent outer core glycosyltransferase (GT) repertoire, including GT6, GT32, and the poorly characterized GT90 family, likely driving outer core assembly and potentially contributing to the unusual phosphodiester-linked hexose decoration. Genome mining additionally identified four putative CPS biosynthetic loci, among which one cluster (cps1; Figure 2d) displayed a genetic organization highly consistent with biosynthesis of the positively charged CPS described here, with glycosyltransferase content closely matching the experimentally determined repeating unit. Notably, the presence of candidate carbohydrate deacetylases (BLV03_RS03015 and BLV03_RS12960) suggests a plausible genetic basis for the N-deacetylation contributing to polysaccharide cationization. Finally, comparative CAZyme and GT profiling within Bacteroidales and other gut-associated Gram-negative bacteria further highlighted that *B. stercoris* DSM 19555 displays a peculiar glycosylation landscape characterized by a GT repertoire partially overlapping with host glycosylation pathways, supporting its potential to produce structurally diverse and host-relevant glycoconjugates.

### *B. stercoris* Glycoconjugates Elicit Attenuated Inflammatory and IL-10-Associated Macrophage Responses

To define the innate immune profile of *B. stercoris* R-LPS and CPS, we first assessed TLR activation using HEK-Blue reporter cells expressing hTLR4/MD-2/CD14, TLR2, TLR2/1, or TLR2/6 (Figure S14a-e). HEK-Blue cells expressing MD-2 and CD14 but not TLR4 were also employed as an additional control. *B. stercoris* R-LPS displayed weak TLR4 agonism, reaching approximately half of the NF-κB activation induced by the potent pro-inflammatory *E. coli* LPS at 100 ng mL^–1^ (*p* < 0.0001, Figure S14a). The CPS showed no TLR4 activation at low concentrations and only modest activation at the highest dose tested (500 ng mL^–1^, Figure S14a). None of them activated the NF-κB pathway in HEK-Blue MD-2/CD14 (Figure S14b). In contrast, both *B. stercoris* glycoconjugates efficiently activated TLR2 and TLR2/1, while neither stimulated TLR2/6, indicating preferential engagement of the TLR2/1 heterodimer (Figure S14c-e). These data, in particular, position *B. stercoris* R-LPS as a mild TLR4 agonist with pronounced TLR2/1-mediated signaling, consistent with emerging features of commensal-derived LPSs such as that of *Akkermansia muciniphila*, which has been associated with beneficial effects on the host.^8^

We next evaluated whether R-LPS or CPS could antagonize TLR4-dependent signaling induced by *E. coli* LPS. *Rhodobacter sphaeroides* LPS (LPS RS), a well-established TLR4 antagonist,^21,22^ was used as a positive control (Figure S14 f,g). HEK-TLR4 cells were preincubated with increasing concentrations of R-LPS or CPS prior to stimulation with 10 ng mL^–1^ of *E. coli* LPS. While LPS RS efficiently impaired TLR4 activation, neither *B. stercoris* R-LPS nor CPS inhibited *E. coli* LPS-induced TLR4-dependent NF-κB activation (Figure S14f,g). To assess the contribution of TLR4 signaling in immune cell lines, THP-1-derived resting macrophages (M0) were used and pretreated with LPS RS before stimulation with *B. stercoris* R-LPS, CPS, or *E. coli* LPS (Figure 3a). As expected, *E. coli* LPS-induced activation was strongly antagonized by LPS RS, whereas NF-κB activation induced by *B. stercoris* R-LPS or CPS remained largely unaffected. These observations are consistent with a limited contribution of canonical TLR4-dependent signaling to their activity.

**Figure 3:**
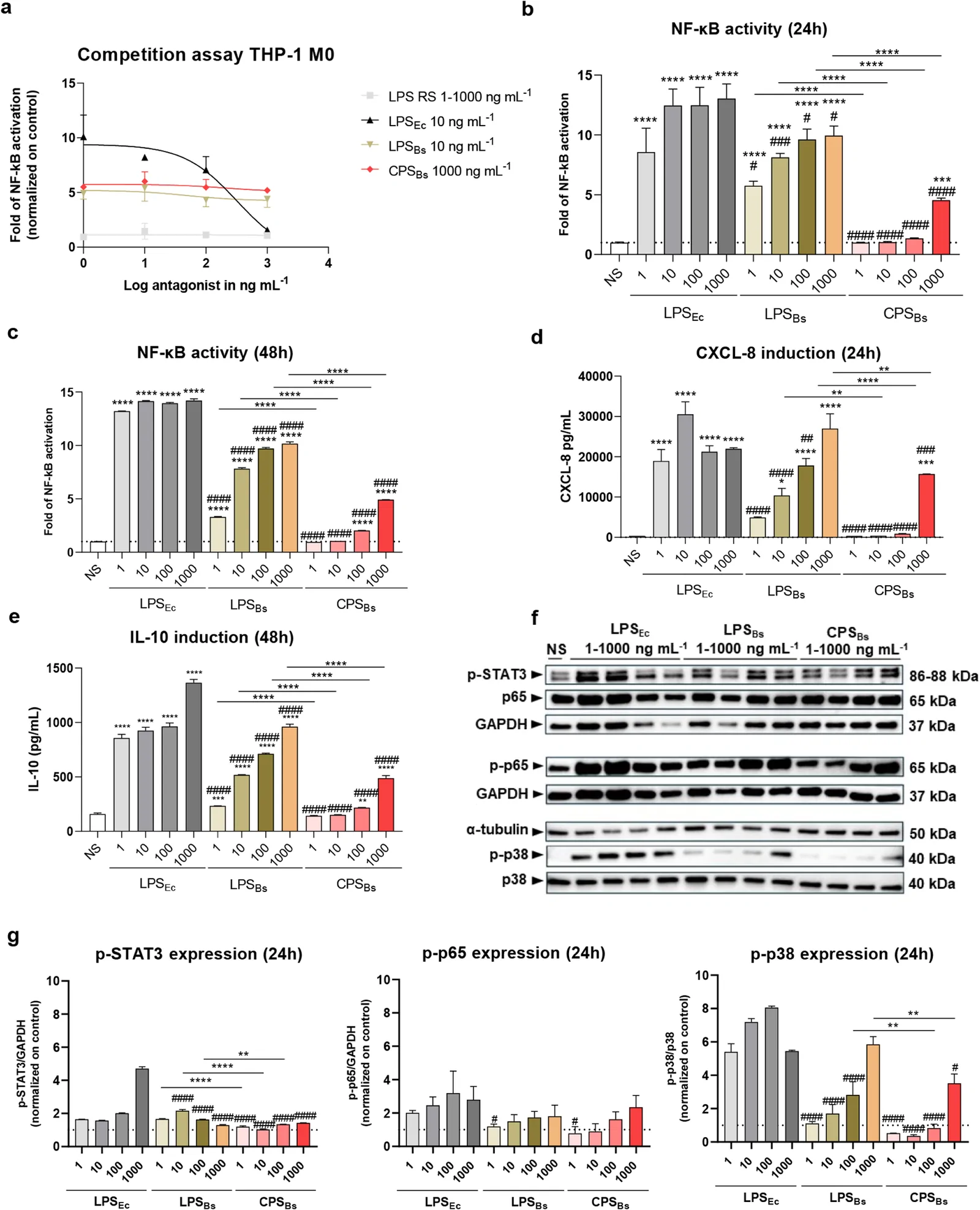
Effects of *B. stercoris* glycoconjugates on THP-1-derived M0 macrophages. (**a**) Competition assay: cells were pretreated with increasing concentrations (1–1000 ng mL^–1^) of *R. sphaeroides* (LPS RS) followed by stimulation with 10 ng mL^–1^ of *B. stercoris* R-LPS (LPS_Bs_) or *E. coli* LPS (LPS_Ec_) or with 1000 ng mL^–1^ of CPS (CPS_Bs_). (**b–e**) Stimulation assays: cells were stimulated with LPS_Ec_, LPS_Bs_, or CPS_Bs_ for 24 or 48 h. NF-κB-dependent SEAP activity (OD 620 nm) was measured and reported in (**b**,**c**), while CXCL-8 and IL-10 secretion were quantified by ELISA and reported in (**d**,**e**). (**f**) Effects of glycoconjugates on protein expression assessed by immunoblotting following stimulation with LPS_Ec_, LPS_Bs_, or CPS_Bs_. (**g**) Densitometric analysis of phospho-p65, phospho-STAT3, and phospho-p38 levels expressed relative to control conditions and normalized to GAPDH or to the corresponding total form. Data are presented as mean ± s.d. of three independent experiments, except for Western blot densitometric analyses, which are reported as mean ± SEM. Statistical significance was determined by one-way ANOVA (*p < 0.05; **p < 0.01; ***p < 0.001; ****p < 0.0001 vs NS; ##*p* < 0.01; ###*p* < 0.001; ####*p* < 0.0001 vs LPS_Ec_ at the corresponding concentration). Asterisks above horizontal lines connecting bars indicate statistically significant differences between LPS_Bs_ and CPS_Bs_ at the corresponding concentration.

We then evaluated the immunostimulatory profile of *B. stercoris* glycoconjugates in THP-1-derived resting macrophages (M0). Both R-LPS and CPS induced significantly lower NF-κB activation compared to *E. coli* LPS across all tested concentrations (1–1000 ng mL^–1^) (Figure 3b,c). Immunoblot analysis confirmed reduced phosphorylation of p65 and p38, while STAT3 (S727) phosphorylation was maintained (Figure 3f,g). Importantly, neither molecule, except for *E. coli* LPS, reduced mitochondrial metabolic activity (Figure S15), indicating limited induction of cellular stress. Consistent with this profile, both *B. stercoris* glycoconjugates significantly increased CXCL-8 and IL-10 production compared with unstimulated controls across multiple concentrations. However, CXCL-8 secretion remained substantially lower than that induced by *E. coli* LPS (Figure 3d), whereas IL-10 production was robust (Figure 3e), supporting a shift toward regulatory immune outputs despite attenuated pro-inflammatory signaling. Notably, at higher concentrations (100–1000 ng mL^–1^), *E. coli* LPS induced lower CXCL-8 release compared to intermediate doses, a behavior that coincided with a marked decrease in cell viability (Figure S15) and is consistent with the onset of endotoxin tolerance in THP-1 cells.^23^ This effect was not observed for *B. stercoris* glycoconjugates, which maintained a consistent cytokine response across the tested concentration range. Accordingly, in preactivated macrophages (M­(IFNγ + LPS)), *E. coli* LPS had minimal impact on NF-κB activity and did not enhance CXCL-8 secretion (Figure S16). In contrast, stimulation with *B. stercoris* glycoconjugates resulted in a reduction of CXCL-8 levels, in some conditions below those of unstimulated controls (Figure S16). In particular, CPS consistently decreased CXCL-8 secretion across all concentrations tested (*p* < 0.05–0.0001, Figure S16), suggesting a potential capacity to modulate ongoing inflammatory responses rather than inducing classical pro-inflammatory activation.

Phenotypic analysis of THP-1 M0 treated with the various glycoconjugates further supported a nonclassical activation profile (Figure S17 for gating strategy). At 1 ng mL^–1^, both R-LPS and CPS significantly increased the proportion of CD206^+^ macrophages compared to resting M0 cells (*p* < 0.01 and *p* < 0.0001, respectively), whereas *E. coli* LPS abolished CD206 expression (Figure 4a). Of note, only CPS significantly increased CD206 mean fluorescence intensity (MFI) (*p* < 0.001), despite both *B. stercoris* glycoconjugates increasing the proportion of CD206^+^ cells, suggesting a more pronounced effect of CPS on CD206 expression. R-LPS induced moderate upregulation of CD200R and CD80 at low doses, although CD80 expression remained lower than in M1 controls (M­(IFNγ + LPS)), consistent with partial activation rather than full classical polarization. The concomitant increase of CD200R, a receptor associated with inhibitory signaling and immune regulation, is in line with the acquisition of a modulatory phenotype.^24^ At the same time, CD14 and HLA-DR expression were sustained at all concentrations compared with M0, indicating preservation of markers associated with innate sensing and antigen presentation. This is noteworthy, as macrophages displaying higher expression of CD206 and CD200R, i.e., features commonly associated with M2-like states, have often been reported to exhibit reduced HLA-DR expression,^25^ whereas its maintenance here may suggest that immunoregulatory features are not accompanied by a loss of immune responsiveness. At higher concentrations, R-LPS shifted toward a more M1-like phenotype, albeit less pronounced than the M­(IFNγ + LPS) control, indicating a dose-dependent tuning of macrophage activation and supporting the notion of an intermediate activation state rather than a binary polarization. Notably, CPS displayed a distinct behavior, inducing HLA-DR and CD14 expression at higher concentrations while maintaining low or negligible CD80 levels and preserving CD206 expression, consistent with an immunomodulatory profile. In addition, CD11c expression, a marker associated with subsets of intestinal macrophages and inflammatory activation,^26^ remained largely stable across *E. coli* LPS and *B. stercoris* R-LPS treatments. In contrast, CPS treatment was associated with a reduction in both the proportion of CD11c^+^ cells and CD11c MFI, with a similar trend observed in M­(IL-4) cells (Figure 4c; see also Figure S18), indicating a shift away from inflammatory-like macrophage subsets.

**Figure 4:**
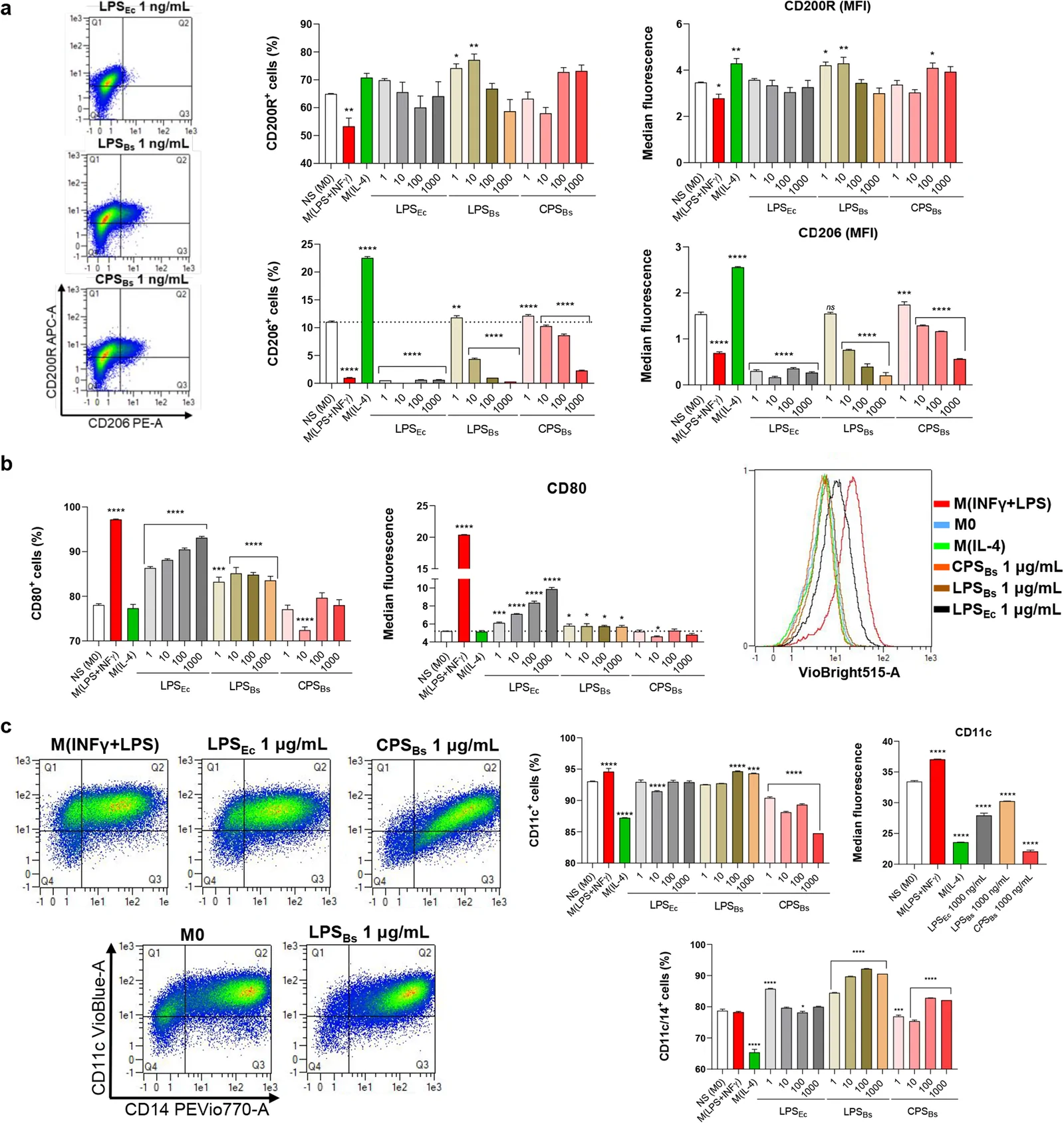
Macrophage activation marker in response to*B. stercoris*glycoconjugates. (**a**) Representative flow cytometry dot plots showing CD206 and CD200R expression on macrophages stimulated for 48 h under different conditions. Quadrants indicate CD200R^+^ (Q1), CD206^+^ (Q3), double-positive (Q2), and double-negative (Q4) populations. Quantification of CD200R^+^ and CD206^+^ macrophages together with their median fluorescence intensity (MFI) from three independent experiments are shown as histograms across all tested conditions. (**b**) Histograms showing the frequency of CD80^+^ cells across all tested concentrations. MFI of CD80 expression in the live cell population is reported for all conditions. Representative overlaid histograms (height-normalized) are shown for the highest concentration of each treatment. (**c**) Representative flow cytometry dot plots showing CD14 and CD11c expression on macrophages stimulated for 48 h under different conditions. Quadrants indicate CD11c^+^ (Q1), CD14^+^ (Q3), double-positive (Q2), and double-negative (Q4) populations. Quantification of CD11c^+^ cells and CD11c^+^/CD14^+^ double-positive populations is shown. MFI of CD11c expression corresponding to the displayed plots is also reported (see also Figure S18). Statistical analysis was performed using one-way ANOVA with Dunnett’s correction, confronting each treatment with unstimulated M0 macrophages. *p < 0.05; **p < 0.01; ***p < 0.001; ****p < 0.0001.

### *B. stercoris* Glycoconjugates Modulate Inflammatory Outputs in an Inflamed Gut Coculture Model

To investigate the behavior of *B. stercoris* R-LPS and CPS in a tissue-relevant context, we employed a Transwell coculture system combining the differentiated colonic human cell line Caco-2 with THP-1-derived M (IFNγ + LPS) macrophages. This widely used human-based model recapitulates key features of a “leaky gut” environment but can be controlled to maintain a moderate inflammatory state without overt barrier disruption, thereby enabling the investigation of immune responses under conditions that more closely resemble physiological or low-grade inflammation rather than severe epithelial damage (Figure 5a). Baseline TEER values (>300 Ω·cm^2^; 581.1 ± 30.9 Ω·cm^2^) confirmed intact epithelial integrity prior to macrophage addition (Figure S19a, day 16). In the coculture system, the presence of activated macrophages induced a moderate reduction in TEER compared to precoculture conditions, consistent with a controlled inflamed epithelial-immune interface (Figure S19a, day 17). Following apical stimulation, *E. coli* LPS, *B. stercoris* R-LPS, and CPS did not induce a relevant drop in TEER over the 18 h time frame compared to the control, indicating the absence of overt barrier disruption. Notably, *B. stercoris* CPS showed a consistent tendency to maintain or slightly increase TEER values relative to control conditions (Figure S19b). However, this effect was not accompanied by significant changes in occludin or claudin-1 levels, nor by alterations in FITC-dextran permeability (not shown), suggesting that epithelial junctional architecture remained largely preserved. Western blot analysis confirmed sustained p65 phosphorylation in basolateral macrophages, consistent with their M1-like activation state (Figure 5c). Accordingly, a significant increase in NF-κB activity was detected only in response to high-dose *E. coli* LPS (*p* < 0.0001; Figure S19d), whereas *B. stercoris* R-LPS and CPS did not further amplify NF-κB activation under these conditions. In epithelial cells, *E. coli* LPS markedly enhanced p65 phosphorylation, while R-LPS induced a comparatively attenuated response, and CPS reduced p65 phosphorylation at intermediate concentrations (Figure 5b). Although these differences, as determined by densitometric analysis, did not reach statistical significance compared to NS (Figure 5b), the overall trend supports a reduced activation profile. Differential STAT3 activation was observed across treatments, indicating distinct signaling outputs within the epithelial-immune interface. Interestingly, cytokine profiling of the basolateral compartment revealed robust secretion of CXCL-8, IL-6, and TNF-α upon *E. coli* LPS stimulation (Figure 5d–g), with IL-6 and TNF-α predominantly increased at the highest concentration tested, accompanied by the expected compensatory induction of IL-10. In contrast, *B. stercoris* R-LPS predominantly induced IL-10 production across concentrations, without significant upregulation of pro-inflammatory cytokines. Similarly, *B. stercoris* CPS elicited dose-dependent IL-10 secretion and selectively increased IL-6 only at the highest concentration, in the absence of significant TNF-α release (Figure 5d–g). Collectively, these data indicate that, within an inflamed but nondisrupted epithelial-immune interface, *B. stercoris* glycoconjugates tend to limit inflammatory amplification by maintaining IL-10 production without broadly increasing pro-inflammatory mediators.

**Figure 5:**
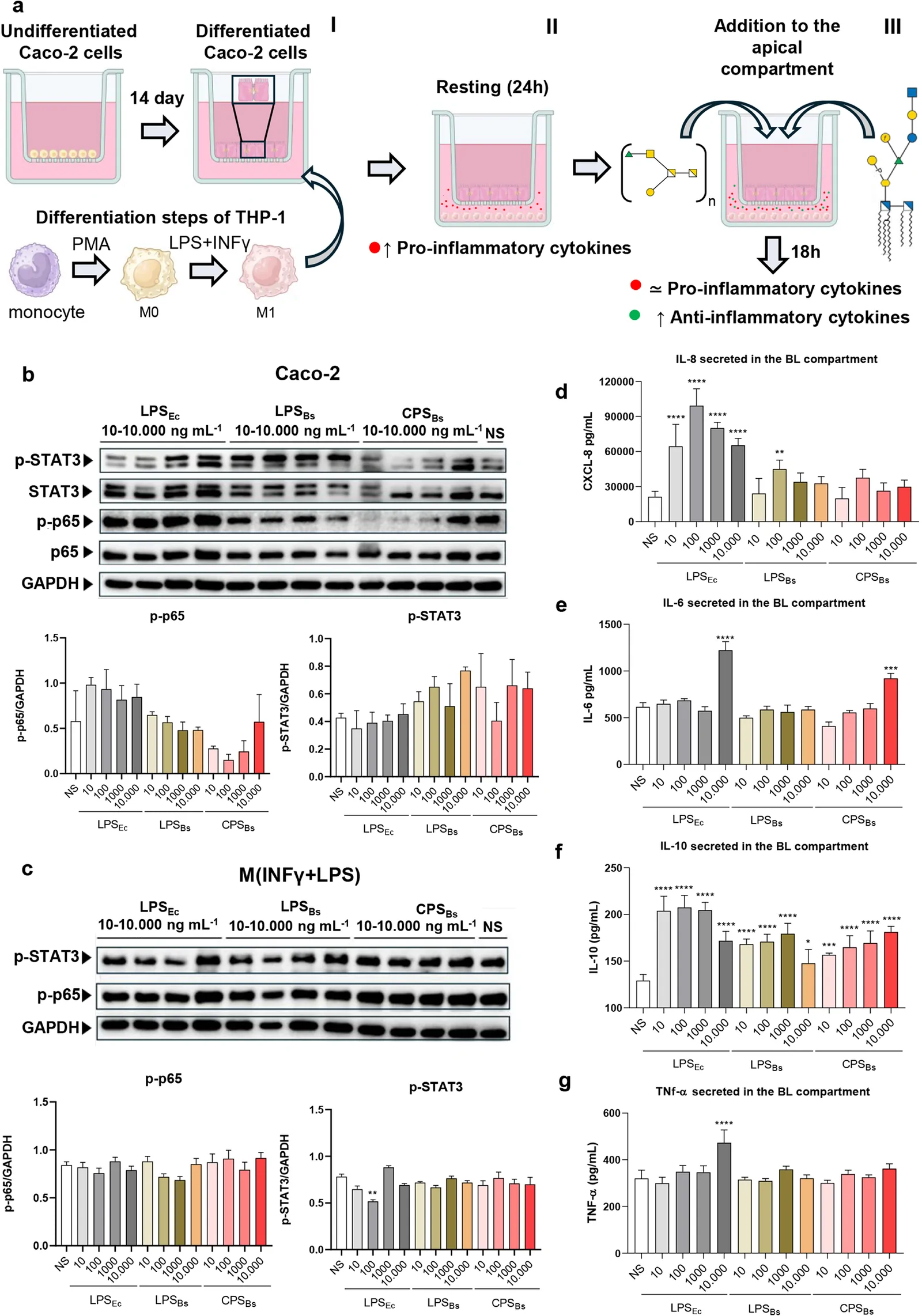
Effects of *B. stercoris* glycoconjugates in a gut coculture model. (**a**) Schematic representation of the experimental design (created with BioRender.com; some elements adapted from Servier Medical Art). (**b**) Effect of 18 h apical stimulation with LPS_Ec_, LPS_Bs_, or CPS_Bs_ on protein expression in differentiated Caco-2 cells. Representative densitometric analysis of phospho-p65 and phospho-STAT3 from two independent experiments is shown. (**c**) Effect of the same treatments on protein expression in THP-1-derived M­(IFNγ + LPS) macrophages. Densitometric analyses of p-p65 and p-STAT3 are shown. (**d–g**) Cytokine levels in the basolateral compartment following 18 h apical stimulation with increasing concentrations of LPS_Ec_, LPS_Bs_, and CPS_Bs_. Data are presented as mean ± s.d. Western blot densitometric analyses are reported as mean ± SEM. Statistical significance was determined by one-way ANOVA (* *p* < 0.05; ** *p* < 0.01; *** *p* < 0.001; **** *p* < 0.0001 vs NS). Quantification by densitometric analysis was performed using Fiji (ImageJ, NIH) and normalized to GAPDH.

## Discussion

Members of the genus *Bacteroides* are key components of the human gut microbiota, contributing to metabolic homeostasis and immune education.^27^ Identifying keystone species and their bioactive molecules is, therefore, critical to understanding how microbial communities influence host physiology. In this context, *B. stercoris* has emerged as a particularly relevant species. It has been associated with metabolic health and anti-inflammatory phenotypes^17^ and has recently been proposed as a biomarker of microbiota resilience, as its abundance appears relatively stable even following major perturbations such as dietary interventions or antibiotic treatment.^28^ However, its relationship with host physiology remains context-dependent and not fully resolved, as it has also been associated with inflammatory and metabolic disorders.^19,20^ These apparently conflicting observations likely reflect the limited understanding of the molecular mechanisms underlying its interaction with the host immune system. Here, we delineate the structural and functional features of the major surface glycoconjugates of *B. stercoris* DSM 19555, providing a molecular framework to interpret its immunological impact on the host. By combining NMR spectroscopy with high-resolution MS and MS/MS, we show that *B. stercoris* produces an atypical R-LPS, lacking the O-antigen moiety, together with an equally uncommon cationic CPS. The R-LPS displays a core OS architecture based on an inner core trisaccharide β-Gal*f*-(1 → 3)-α-Rha*p*-(1 → 5)-α-Kdo, shared with *B. eggerthii*, *Phocaeicola vulgatus*, and *P. dorei* ^6,11,26^ but further modified by a hexose residue, likely glucose, linked to the Kdo at O-4 via a phosphodiester bridge, with the same Kdo also substituted at O-7 by a *P*EtN group. This was in agreement with our *in silico* analyses, which revealed a conserved inner core biosynthetic module shared with *B. eggerthii* and *P. vulgatus*, including a *waaP*-like kinase, rhamnosyltransferase, and galactofuranosyltransferase activities, organized within a predicted operon, consistent with the assembly of the above trisaccharide motif. In addition, the R-LPS features a nonstoichiometric terminal β-Glc*p*NAc residue in the outer region of the core OS. While this sugar has been previously reported in *B. eggerthii* together with β-Glc*p* and α-Gal*p*, the overall architecture differs from related species, as no fucose residue, neither at the nonreducing end (as in *B. eggerthii*)^11^ nor as an internal substituent (as observed in *P. vulgatus* and *P. dorei*)^6,29^ was detected. The absence of fucose is particularly notable, given the presence in *B. stercoris* DSM 19555 genome of a GT11-family enzyme, typically associated with fucosylation and also identified in fucosylated LPSs from species such as *A. muciniphila*, *P. vulgatus*, and *B. eggerthii*.^11^ This suggests that, in *B. stercoris* DSM 19555, this pathway might be inactive, differently regulated, or redirected toward alternative substrates. As for the lipid A portion, this consists of a complex blend of *mono*-phosphorylated and tetra- and penta-acylated species, consistent with *Bacteroides* lipid A features and with the genomic identification of a single *lpxL* homologue, the absence of *lpxM*, and a full asset of lipid A-modifying enzymes such as homologues to *lpxE/lpxF* phosphatases and a *pagL* deacylase (Table S5).^30,31^

The CPS represents an equally novel glycan, consisting of a branched pentasaccharide repeating unit containing α-Rha*p* and β-Gal*p* and enriched in amino sugars, including two Gal*p*NAc and one nonacetylated Gal*p*N residue, which collectively confer an overall positive charge to the polymer. Genome mining identified four putative CPS loci. Among these, cps1 was characterized by a compatible glycosyltransferase repertoire and the canonical *upxY*-*upxZ* regulatory architecture of active *Bacteroides* CPS loci,^32^ thus emerging as the most likely biosynthetic cluster for the structure described here. Notably, the presence of integrase/recombinase genes flanking cps1 suggests phase-variable regulation of CPS expression,^30,33^ a mechanism known to modulate surface glycan display in response to environmental cues. Such structural and regulatory features are consistent with a dynamic role of the CPS in host-microbe interactions, potentially contributing to immune modulation, niche adaptation, and persistence within the gut environment.^15,16,32,34^ Indeed, many Gram-negative gut commensals (e.g., *B. thetaiotaomicron* and *B. fragilis*) encode multiple phase-variable CPSs that enable rapid adaptation to fluctuating environmental and immunological pressures.^13,32,34^ In addition, specific capsular structures, particularly zwitterionic CPSs, have been shown to promote immune tolerance by modulating cytokine production toward anti-inflammatory profiles.^16^ In this context, the positively charged nature of the *B. stercoris* CPS represents an unusual feature within the *Bacteroides* genus and, to the best of our knowledge, among gut-associated CPSs described to date. Together with the distinctive structural characteristics of the R-LPS, this prompted an investigation of their immunological activity.

Consistent with their distinctive chemical features, *B. stercoris* glycoconjugates displayed a “noncanonical” immunological profile. While *E. coli* LPS induced potent NF-κB activation and a robust pro-inflammatory response accompanied by compensatory negative-feedback IL-10 production, both *B. stercoris* R-LPS and CPS elicited only limited pro-inflammatory responses while maintaining significant IL-10 production. As for the R-LPS, this behavior contrasts with prototypical TLR4-activating LPSs and is compatible with a distinct activation profile, potentially involving differential NF-κB activation and/or cross-talk with STAT3-associated regulatory pathways. The preservation of STAT3 phosphorylation across conditions supports this interpretation, though its direct contribution to IL-10 induction and potential role in macrophage polarization remain to be clarified.^35,36^ Moreover, the preferential activation of TLR2/TLR1, together with the limited contribution of TLR4-dependent signaling, further distinguishes *B. stercoris* R-LPS from pathogen-associated LPSs. Similar receptor usage has been reported for structurally atypical LPSs from commensal bacteria, supporting the concept that specific lipid A and glycan features can redirect innate immune recognition across distinct receptor pathways, ultimately leading to attenuated inflammatory outputs.^6,8,9^ Notably, a comparable receptor engagement has been described for polysaccharide A from *B. fragilis*, which activates the TLR2/TLR1 heterodimer through a lipid A-like-associated anchor.^37^ In this context, the contribution of a very minor lipid A-like-associated moiety in *B. stercoris* CPS cannot be excluded. However, the observation that TLR2/TLR1 activation occurs only at higher concentrations, compared to R-LPS, suggests a lower efficiency of receptor engagement. This difference may reflect a reduced abundance or accessibility of such lipid-associated features or, alternatively, point to additional structural determinants, whereby the polysaccharide architecture itself contributes to receptor activation.

Strikingly, in M­(IFNγ + LPS) macrophages, CPS reduced CXCL-8 production, indicating an ability not only to avoid triggering inflammation but also to actively modulate ongoing inflammatory responses. This behavior is consistent with immunomodulatory mechanisms described for probiotic-derived extracellular vesicles^38^ and specific commensal CPSs that contribute to intestinal immune homeostasis.^39,40^ Notably, R-LPS and CPS elicited distinct yet partially overlapping activation profiles in THP-1 macrophages. R-LPS induced a moderate activation state, characterized by concomitant upregulation of CD206, CD200R, and CD80 at low doses and preservation of CD14 and HLA-DR expression across concentrations, consistent with a modulatory phenotype retaining antigen-presenting competence. The shift toward a more M1-like profile at higher concentrations further supports a dose-dependent tuning of macrophage activation. By contrast, CPS displayed a distinct phenotypic profile during macrophage polarization, characterized by high CD206 expression at low to intermediate concentrations (1–100 ng mL^–1^), low or negligible CD80 levels, and induction of HLA-DR and CD14 at higher concentrations. Analysis of CD11c further refined this observation. While CD11c^high^CCR2^+^CX3CR1^+^ populations are typically associated with inflammatory states, CD11c^–^ subsets are linked to homeostatic, tolerogenic conditions.^41^ In our system, CD11c downregulation was mostly enhanced in CPS-treated macrophages and M­(IL-4) controls but not following R-LPS or *E. coli* LPS stimulation. This indicates that the two *B. stercoris* glycoconjugates are associated with partially distinct macrophage activation profiles despite shared low-inflammatory features and identifies CPS as the more potently immunomodulatory component. It is therefore tempting to hypothesize that R-LPS may provide a basal low-inflammatory interface with host innate receptors, whereas the predicted phase-variable CPS may add a more dynamically regulated immunomodulatory layer capable of influencing macrophage responses under inflammatory conditions. These observations were further evaluated in a tissue-mimicking coculture system, where immune and epithelial compartments interact. Under conditions of controlled inflammation without overt barrier disruption,^41^ *B. stercoris* glycoconjugates preserved epithelial integrity while selectively shaping cytokine outputs. In contrast to *E. coli* LPS, both *B. stercoris* R-LPS and CPS promoted IL-10-dominated responses without concomitant increases in pro-inflammatory cytokines like CXCL-8. This profile is consistent with a macrophage response oriented toward inflammatory restraint rather than amplification of pro-inflammatory activation^42–44^ and parallels observations reported for probiotic-derived components in similar coculture systems.^45^

Collectively, these data support a model in which *B. stercoris* surface glycoconjugates behave as active modulators of innate immune signaling. By maintaining IL-10 production while attenuating broad pro-inflammatory outputs, they may contribute to a low-inflammatory host-microbe interface. This framework is consistent with the enrichment of *B. stercoris* in metabolically healthy individuals and fiber-rich dietary contexts. At the same time, the weak inflammatory potential of its surface glycoconjugates may help explain why the bacterium can persist even in inflamed intestinal environments, such as Crohn’s disease, without necessarily acting as a direct driver of tissue inflammation. Moreover, as stated above, it is worth noting that the CPS, which displays the strongest immunomodulatory activity, is encoded within a phase-variable biosynthetic locus. Accordingly, differences in its expression across strains or environmental conditions could influence the surface glycan composition and may contribute to the context-dependent associations reported for *B. stercoris*, although this remains to be experimentally demonstrated. By contrast, LPS is expected to remain constitutively expressed. The coexistence with CPS, a common feature among gut-associated Gram-negative bacteria,^15^ likely enables fine-tuning of the bacterial surface and its interactions with the host through modulation of CPS expression while maintaining the structural and physiological functions of LPS.

From a translational perspective, these findings provide a molecular basis for future studies aimed at linking *B. stercoris* glycoconjugate signatures to the host inflammatory status. In particular, they may inform efforts to stratify human-derived commensal isolates, investigate bacterial surface glycoconjugate signatures associated (or not) with intestinal inflammation, and assess microbiota-derived glycoconjugates as candidate postbiotic immunomodulators. However, these implications remain prospective and require experimental and clinical validation. In this context, some limitations of this study should be acknowledged. First, although our data indicate distinct receptor engagement and immunomodulatory profiles for R-LPS and CPS, dedicated studies will be required to identify additional receptors and downstream signaling pathways involved in these responses.^46^ Second, this work focuses on the reference strain *B. stercoris* DSM 19555. Whether the R-LPS and cationic CPS described here are conserved, differentially expressed, or associated with health versus inflammatory disease in human-derived isolates remains to be established through targeted LPS/CPS glycomic and lipid A profiling of clinical cohorts. Nevertheless, our findings reinforce an emerging view in which surface glycans of gut Bacteroidales act as functional immunomodulators that shape host immune responses.^5,12,15^ Future work should aim to resolve the contribution of individual glycan motifs in the cross-talk with other immune receptors and validate these mechanisms in physiologically relevant *in vivo* models.

## Supplementary Material



## Data Availability

Data supporting the findings of this study are available within the paper and its Supporting Information file. All other data are available from the corresponding author upon reasonable request.

## Notes

NMR, MS, and MS/MS spectra; additional *in vitro* tests and experimental details; and materials and methods, including bioinformatics data (PDF)

## Abbreviations

CAZyme: carbohydrate-active enzyme
CCR2: C–C chemokine receptor type 2
CD11c: cluster of differentiation 11c
CD14: cluster of differentiation 14
CD80: cluster of differentiation 80
CD200R: CD200 receptor
CD206: cluster of differentiation 206
CPS: capsular polysaccharide
CX3CR1: CX3C chemokine receptor 1
CXCL-8: C-X-C motif chemokine ligand 8
DSM: Deutsche Sammlung von Mikroorganismen and Zellkulturen (German Collection of Microorganisms and Cell Cultures)
ELISA: enzyme-linked immunosorbent assay
ESI: electrospray ionization
FITC: fluorescein isothiocyanate
Gal*f*: β-galactofuranose
Gal*p*: galactopyranose
Gal*p*N: 2-amino-2-deoxy-β-galactopyranose
Gal*p*NAc: 2-acetamido-2-deoxy-β-galactopyranose
GAPDH: glyceraldehyde-3-phosphate dehydrogenase
Glc*p*: β-glucopyranose
Glc*p*NAc: 2-acetamido-2-deoxy-β-glucopyranose
GT: glycosyltransferase
HEK: human embryonic kidney cells
HLA-DR: human leukocyte antigen DR isotype
HMBC: heteronuclear multiple bond correlation
HSQC: heteronuclear single quantum coherence
IL-6: interleukin-6
IL-10: interleukin-10
Kdo: 2-keto-3-deoxy-*manno*-octulosonic acid
LPS: lipopolysaccharide
M0: unstimulated (resting) macrophages
M1: classically activated (pro-inflammatory) macrophages
M2: alternatively activated (regulatory) macrophages
M­(IFNγ + LPS): classically activated (M1) macrophages
M­(IL-4): alternatively activated (M2) macrophages
M­(IL-10): M2c-polarized macrophages
MD-2: myeloid differentiation factor 2
MFI: median fluorescence intensity
MS: mass spectrometry
MS/MS: tandem mass spectrometry
NF-κB: nuclear factor kappa B
NMR: nuclear magnetic resonance
NOESY: nuclear Overhauser effect spectroscopy
OD: optical density
*P*EtN: 2-aminoethyl phosphate
Rha*p*: α-L-rhamnopyranose
R-LPS: rough-type lipopolysaccharide
ROESY: rotating-frame Overhauser effect spectroscopy
SDS-PAGE: sodium dodecyl sulfate-polyacrylamide gel electrophoresis
SEAP: secreted embryonic alkaline phosphatase
STAT3: signal transducer and activator of transcription 3
TEER: transepithelial electrical resistance
THP-1: human monocytic leukemia cell line
TLR1: Toll-like receptor 1
TLR2: Toll-like receptor 2
TLR4: Toll-like receptor 4
TLR6: Toll-like receptor 6
TNF-α: tumor necrosis factor alpha
