# Supplementary material for: Noncanonical Surface Glycoconjugates from Bacteroides stercoris DSM 19555 Reprogram Innate Immune Responses
Source: JACS Au. 2026 Jul 25;6(8):4763–75. doi: 10.1021/jacsau.6c00878 (PMC13508109; doi:10.1021/jacsau.6c00878)
Supplement: Supplementary file 1 [file au6c00878_si_001.pdf]

Supporting Information

Non-canonical surface glycoconjugates from *Bacteroides stercoris* DSM 19555 reprogram innate immune responses

Luca De Simone Carone, Stefania De Chiara, Marcello Mercogliano, Francesca Olmeo, Valentina Mazziotti, Alba Silipo, Freda M. Farquharson, Petra Louis, Antonio Molinaro, Flaviana Di Lorenzo

Supporting Tables

Table S1. ^{1}H, ^{13}C, and ^{31}P chemical shift values of the core OS obtained after full deacylation of the *B. stercoris* R-LPS.

Table S2. Negative-ion ESI-MS data and proposed composition of *B. stercoris* R-LPS related ions.

Table S3. Fatty acid content of the R-LPS isolated from *B. stercoris* DSM 19555

Table S4. ^{1}H and ^{13}C chemical shift values of the CPS isolated from *B. stercoris*.

Table S5. Lipid A biosynthesis and modification genes in *B. stercoris* DSM 19555

Supporting Figures

Figure S1. (600 MHz, 298 K, D_2O, Tables S1 and S4). ^{1}H NMR spectra of the core OS and CPS of *B. stercoris* DSM 19555

Figure S2. (600 MHz, 298 K, D_2O, Table S1). ^{1}H and $^{1}H,^{13}C$ HSQC spectra of the core OS of *B. stercoris* DSM 19555

Figure S3. (400 MHz, 298 K, D_2O, Table S1). ^{1}H, ^{31}P HSQC spectrum of the core OS of *B. stercoris* DSM 19555

Figure S4. (600 MHz, 298 K, D_2O, Table S1). ^{1}H, COSY and TOCSY spectra of the core OS of *B. stercoris* DSM 19555

Figure S5. Zoom of the negative-ion ESI MS/MS of $[M-2H]^{2-}$ ion at *m/z* 1554.305

Figure S6. Zoom of the negative-ion ESI MS/MS of $[M-H]^{-}$ ion at *m/z* 1419.349

Figure S7. MALDI-TOF MS spectra of the lipid A from *B. stercoris* DSM 19555 R-LPS

Figure S8. (600 MHz, 298 K, D_2O, Table S4). ^{1}H and $^{1}H,^{13}C$ HSQC spectra of the CPS of *B. stercoris* DSM 19555

Figure S9. (600 MHz, 298 K, D_2O, Table S4). ^{1}H, COSY and TOCSY spectra of the CPS of *B. stercoris* DSM 19555

Figure S10. Bioinformatic prediction of the putative core OS biosynthetic loci of *B. stercoris* DSM 19555.

Figure S11. GT family distribution across *Bacteroides* genomes

Figure S12. Bioinformatic prediction of the putative CPS biosynthetic loci of *B. stercoris* DSM 19555.

Figure S13. CAZyme category distribution across *Bacteroides* genomes.

Figure S14. NF-κB activation in HEK-Blue TLR cells

Figure S15. THP-1 cell viability assessed by MTT assay.

Figure S16. NF-κB activatoin and IL-8 secretion in THP-1-derived M(INFγ + LPS)

Figure S17. Gating strategy

Figure S18. Phenotypic analysis of THP-1 M0.

Figure S19. Effects on intestinal epithelial barrier function and tight junction expression.

Supporting notes

Supporting note 1. Structural characterization of the R-LPS from *B. stercoris* DSM 19555

Supporting note 2. Structural characterization of the CPS from *B. stercoris* DSM 19555

Supporting note 3. Bioinformatic analysis of putative genes encoding for *B. stercoris* DSM 19555 R-LPS biosynthesis.

Supporting note 4. Bioinformatic analysis of putative genes encoding for *B. stercoris* DSM 19555 CPS biosynthesis.

Supporting note 5. Comparative CAZyme and GT family profiling across Bacteroidales

Supporting note 6. Methods

Table S1. ^{1}H, ^{13}C, and ^{31}P chemical shift values of the core OS obtained upon full deacylation of the *B. stercoris* DSM 19555 R-LPS.

	**1**	**2**	**3**	**4**	**5**	**6**	**7**	**8**
**A**	5.52	3.24	3.79	3.27	4.08	4.13/3.79		
6-α-Glc*p*N1*P*	*90.0*	*54.3*	*69.8*	*70.0*	*73.0*	*68.7*		
	^{31}P **1.84**							
**B**	5.54	4.17	4.00	3.47	3.87	1.20		
2,3-α-Rha*p*	*99.0*	*80.7*	*77.8*	*71.0*	*70.8*	*17.1*		
**B'**	5.41	4.21	4.00	3.47	3.87	1.26		
2,3-α-Rha*p*	*99.2*	*79.8*	*77.8*	*71.0*	*70.8*	*17.3*		
**C**	5.12	4.06	3.99	3.95	3.72	3.57		
t-β-Gal*f*	*109.0*	*81.3*	*76.0*	*82.1*	*70.5*	*62.8*		
**D**	4.96	2.87	3.53	3.46	3.53	3.50		
6-β-Glc*p*N	*99.4*	*55.6*	*72.2*	*69.4*	*74.5*	*62.5*		
**D'**	4.91	2.87	3.52	3.46	3.58	3.50		
6-β-Glc*p*N	*99.7*	*55.6*	*72.2*	*69.4*	*74.5*	*62.5*		
**E**	4.93	3.90	4.00	4.16	4.15	3.80/3.67		
3-α-Gal*p*	*97.6*	*71.3*	*79.8*	*69.1*	*74.0*	*60.2*		
**E'**	4.88	3.73	3.84	3.92	3.92	3.80/3.67		
t-α-Gal*p*	*98.2*	*68.2*	*69.4*	*69.3*	*72.4*	*60.2*		
**F**	4.87	2.95	3.70	3.44	3.38	3.64		
t-β-Glc*p*N	*100.9*	*56.1*	*72.1*	*69.6*	*75.9*	*61.1*		
**H**	4.50	3.23	3.48	3.38	3.38	3.80/3.62		
6-β-Glc*p*	*104.0*	*73.2*	*74.3*	*69.4*	*75.7*	*65.9*		
**H'**	4.56	3.24	3.47	3.38	3.38	3.82/3.73		
6-β-Glc*p*	*103.9*	*73.2*	*74.0*	*69.5*	*75.7*	*65.2*		
**K**	-----	-----	2.02/1.82	4.15	4.06	3.90	4.20	3.84
5-α-Kdo4*P*7*P*EtN	*176.9*	*99.7*	*34.4*	*67.2*	*72.8*	*69.3*	*73.9*	*65.4*
				^{31}P **1.35**			^{31}P **0.41**	
*P*EtN	3.92	3.15						
	62.0	*44.4*						

Table S2. Negative-ion ESI MS data and proposed composition of *B. stercoris* R-LPS. Core OS comprises four hexoses (Hex, monoisotopic mass 162.0528), one acetylated glucosamine (HexNAc, monoisotopic mass 203.0794), one deoxyhexose (dHex, monoisotopic mass 146.0579), one Kdo (monoisotopic mass 220.0583), one 2-aminoethyl phosphate (*P*EtN, monoisotopic mass 123.0087) and one phosphate (monoisotopic mass 79.9663).

**Observed ion (*m/z*)**		**Molecular mass (Da)**		
**$(M+Na-2H)^{2-}$**	**$(M-2H)^{2-}$**	**Observed (average)**	**Calculated**	**Proposed composition**
1565.293	1.554.305	3110.119	3109.640	**$LipA_{1689}$ + core OS**
1503.785	1492.796	2987.102	2986.631	**$LipA_{1689}$ + core OS - *P*EtN**
1463.753	1452.765	2907.039	2906.561	**$LipA_{1689}$ + core OS - HexNAc**
-	1427.193	2855.415	2855.415	**$LipA_{1433}$ + core OS**
-	1371.740	2744.508	2744.508	**$LipA_{1689}$ + core OS - HexNAc - Hex**

Table S3. Fatty acid composition of the R-LPS isolated from *B. stercoris* DSM 19555. Fatty acids were analyzed by GC-MS as methyl ester derivatives. All 3-hydroxy fatty acids were assigned the (*R*) absolute configuration. For *anteiso*-branched acyl chains, the configuration is tentatively designated as (*S*), based on its prevalence in bacteria, although definitive assignment remains to be established.

Fatty acid composition
iso and *anteiso* pentadecanoic acid (*i*15:0 and *a*15:0)
pentadecanoic acid (15:0)
hexadecanoic acid (16:0)
iso and *anteiso* heptadecanoic acid (*i*17:0 and *a*17:0))
iso 3-hydroxypentadecanoic acid [*i*15:0 (3-OH)
3-hydroxypentadecanoic acid [15:0 (3-OH)]
3-hydroxyhexadecanoic acid [16:0 (3-OH)]
3-hydroxyheptadecanoic acid [17:0 (3-OH)]

Table S4. ^{1}H and ^{13}C chemical shift values of the B. *stercoris* DSM 19555 CPS obtained obtained by hot phenol-water extraction and subsequent purification.

	**1**	**2**	**3**	**4**	**5**	**6**
**L**<br>4-α-Rha*p*	4.72	3.62	3.68	3.47	3.90	1.16
	101.0	*70.7*	*70.3*	*79.9*	*67.1*	*16.8*
**M**<br>4-β-Gal*p*NAc	4.65	3.63	3.38	3.51	3.64	3.75/3.56
	101.5	*55.0*	*74.2*	*79.6*	*72.7*	*59.9*
**N**<br>4,6-β-Gal*p*N	4.56	4.06	4.24	4.15	3.82	3.97/3.83
	101.0	*52.0*	*72.5*	*74.3*	*73.2*	*69.2*
**O**<br>*t*-β-Gal*p*	4.53	3.47	3.56	3.81	3.56	3.69
	103.5	*70.9*	*72.5*	*68.5*	*74.7*	*60.8*
**P**<br>3-β-Gal*p*NAc	4.52	3.70	3.47	3.38	3.38	3.80
	100.1	*55.2*	*81.5*	*68.3*	*75.9*	*60.5*

Table S5. Lipid A biosynthesis and modification genes in *B. stercoris* DSM 19555

Gene	Predicted function	Copies	Relevance to *B. stercoris* R-LPS structure	Locus tag (DSM 19555)
**LpxA**	UDP-*N*-acetylglucosamine *O*-acyltransferase	2	Initiates lipid A biosynthesis; two copies consistent with other Bacteroidales	*BLV03_RS12590, BLV03_RS02440*
**LpxC**	UDP-3-*O*-acyl-GlcNAc deacetylase	1	Committed step of lipid A pathway	*BLV03_RS12595*
**LpxD**	UDP-3-*O*-acyl-GlcN *N*-acyltransferase	1	Adds second 3-OH fatty acid	*BLV03_RS12600*
**LpxH**	UDP-2,3-diacylglucosamine hydrolase	1	Cleaves pyrophosphate; *Bacteroides* use LpxH (not LpxI)	*BLV03_RS16345*
**LpxB**	Lipid A disaccharide synthase (GT19)	1	Joins two lipid X units	*BLV03_RS01195*
**LpxK**	Tetraacyldisaccharide-4-*P* kinase	1	Adds 4'-phosphate to lipid A	*BLV03_RS08055*
**KdtA (WaaA)**	Kdo transferase (GT30)	1	Transfers single Kdo to lipid IV_A; consistent with mono-Kdo in *Bacteroides*	*BLV03_RS09670*
**LpxL**	Lipid A lauroyltransferase	1	Secondary acyltransferase; single copy explains penta-acylation	*BLV03_RS00695*
**LpxM**	Lipid A myristoyltransferase	0	Absent; consistent with penta- (not hexa-) acylated lipid A	—
**LpxE**	Lipid A 1-phosphatase	1	Removes 1-phosphate; yields *mono*-phosphorylated lipid A	*BLV03_RS03165*
**LpxF**	Lipid A 4'-phosphatase	1	Removes 4'-phosphate; contributes to mono-phosphorylation	*BLV03_RS09540*
**PagL**	Lipid A 3-*O*-deacylase	1	3-*O*-deacylation	*BLV03_RS08970*
**EptA**	*P*EtN transferase (lipid A)	0	—	—
**EptB**	*P*EtN transferase (Kdo)	1	Adds *P*EtN to Kdo; confirmed by NMR (O-7 of Kdo)	*BLV03_RS06470*
**MsbA**	Lipid A flippase	1	Translocates LPS across inner membrane	*BLV03_RS02290*

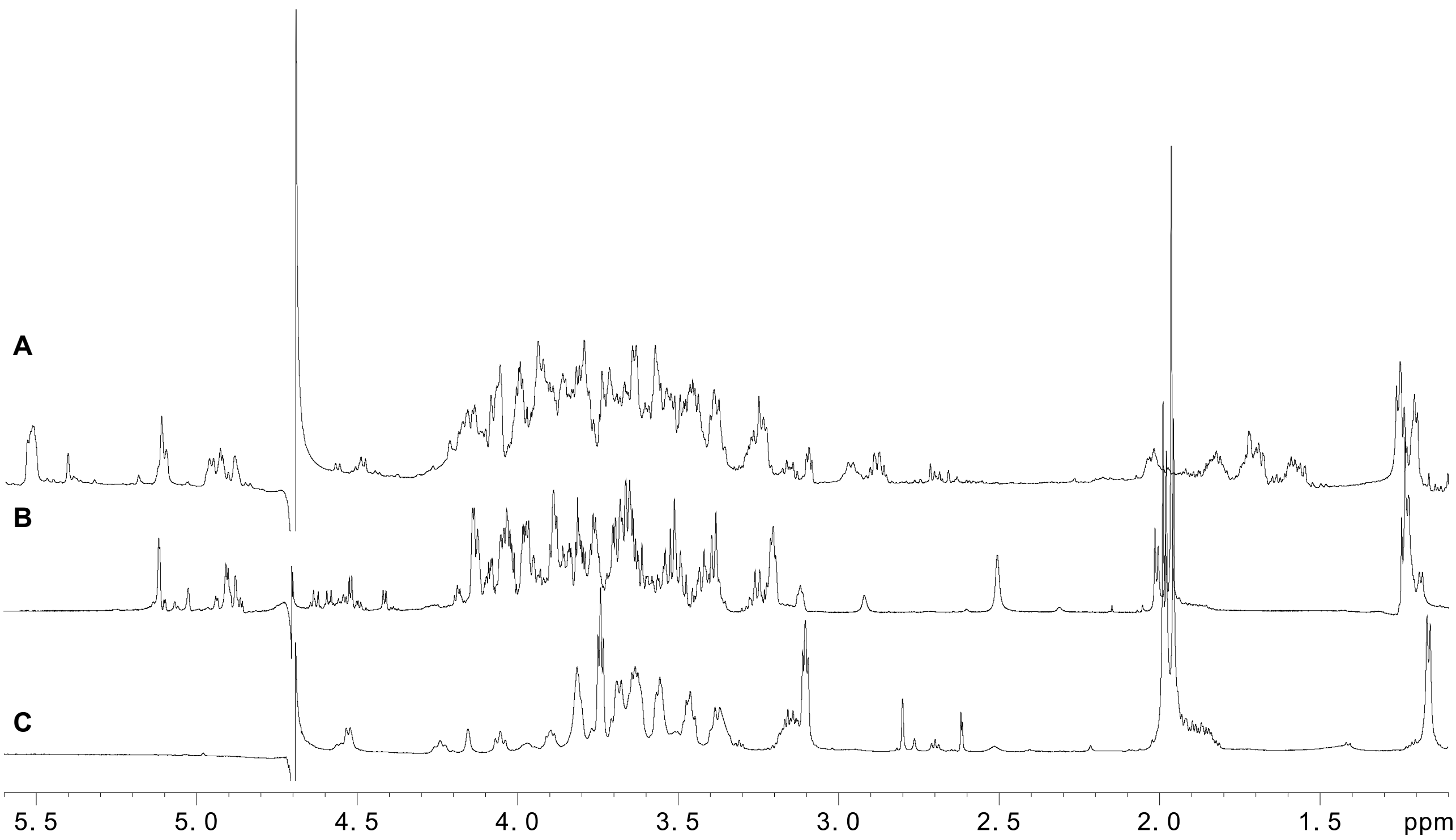

Figure S1. ^{1}H NMR spectra of (**A**) fully deacylated R-LPS, (**B**) mild acid hydrolysis product of R-LPS, and (**C**) CPS from *B. stercoris*

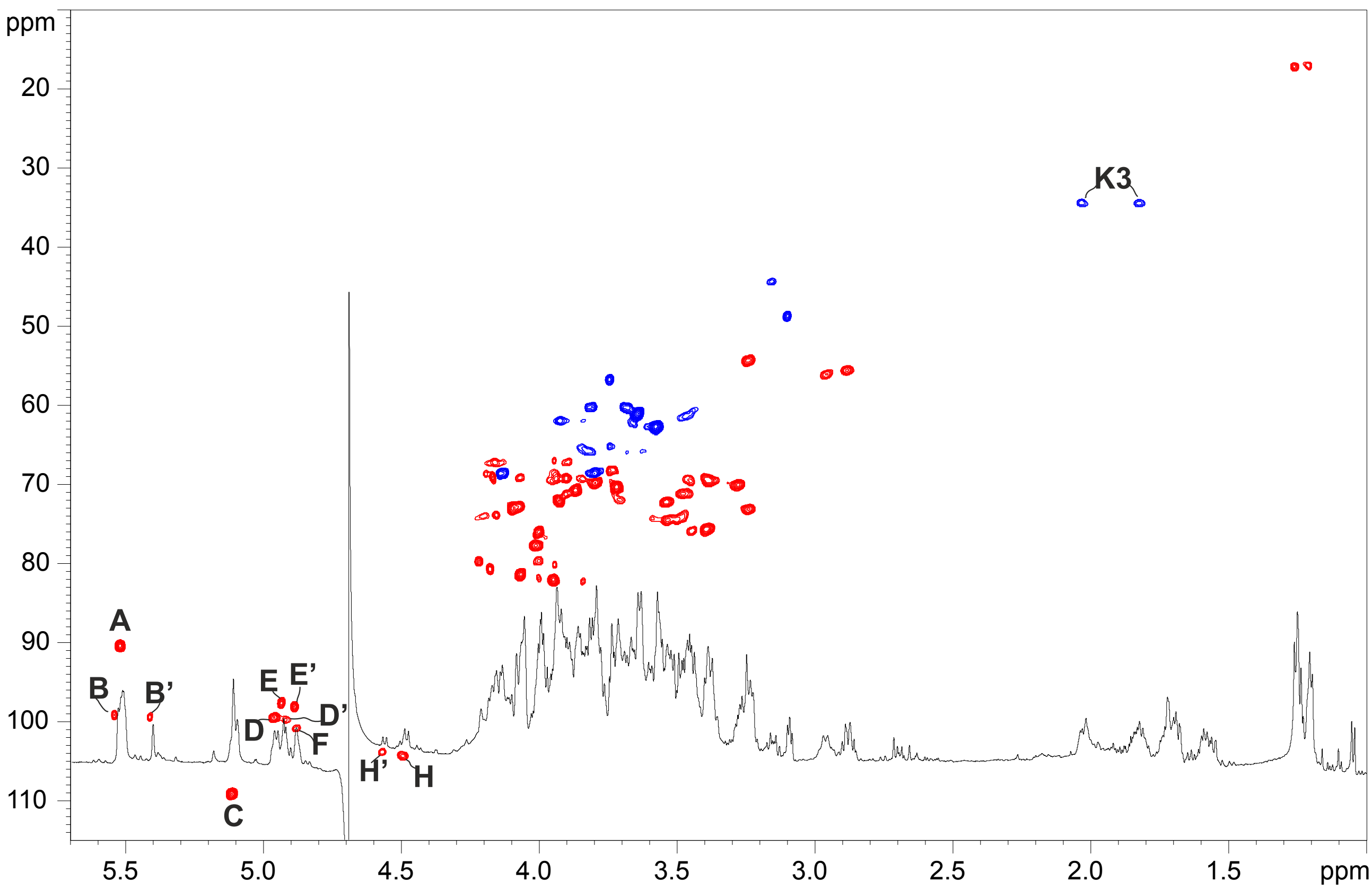

Figure S2. (600 MHz, 298 K, D_2O, Table S1). Superimposed ^{1}H and $^{1}H,^{13}C$ HSQC (red and blue) spectra of the core OS obtained after full deacylation of R-LPS from *B. stercoris*.

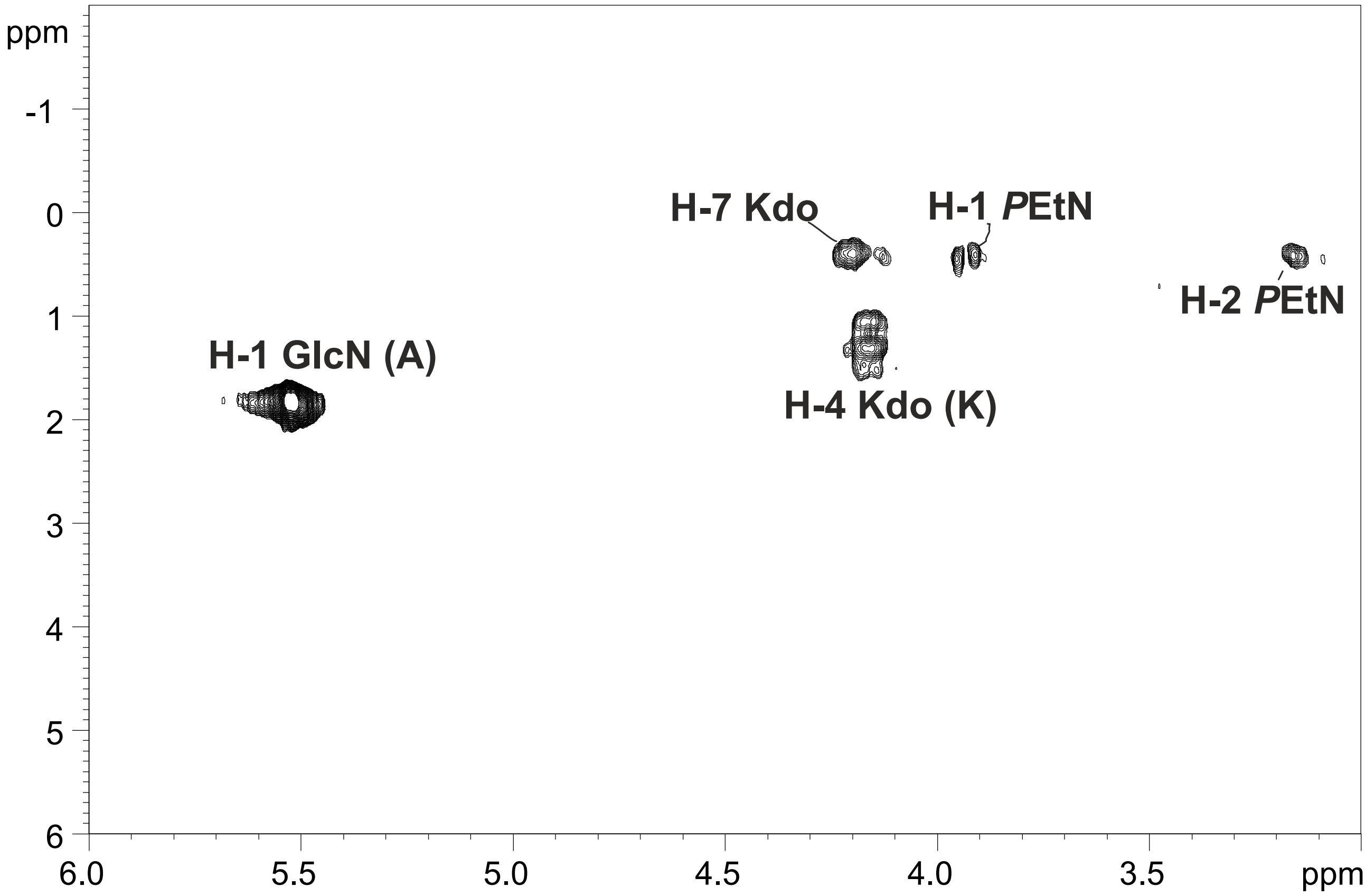

Figure S3. (400 MHz, 298 K, D_2O, Table S1). Section of the $^{1}H,^{31}P$ HSQC spectrum of the core OS of *B. stercoris*. Signals of spin system are designated as in Table S1.

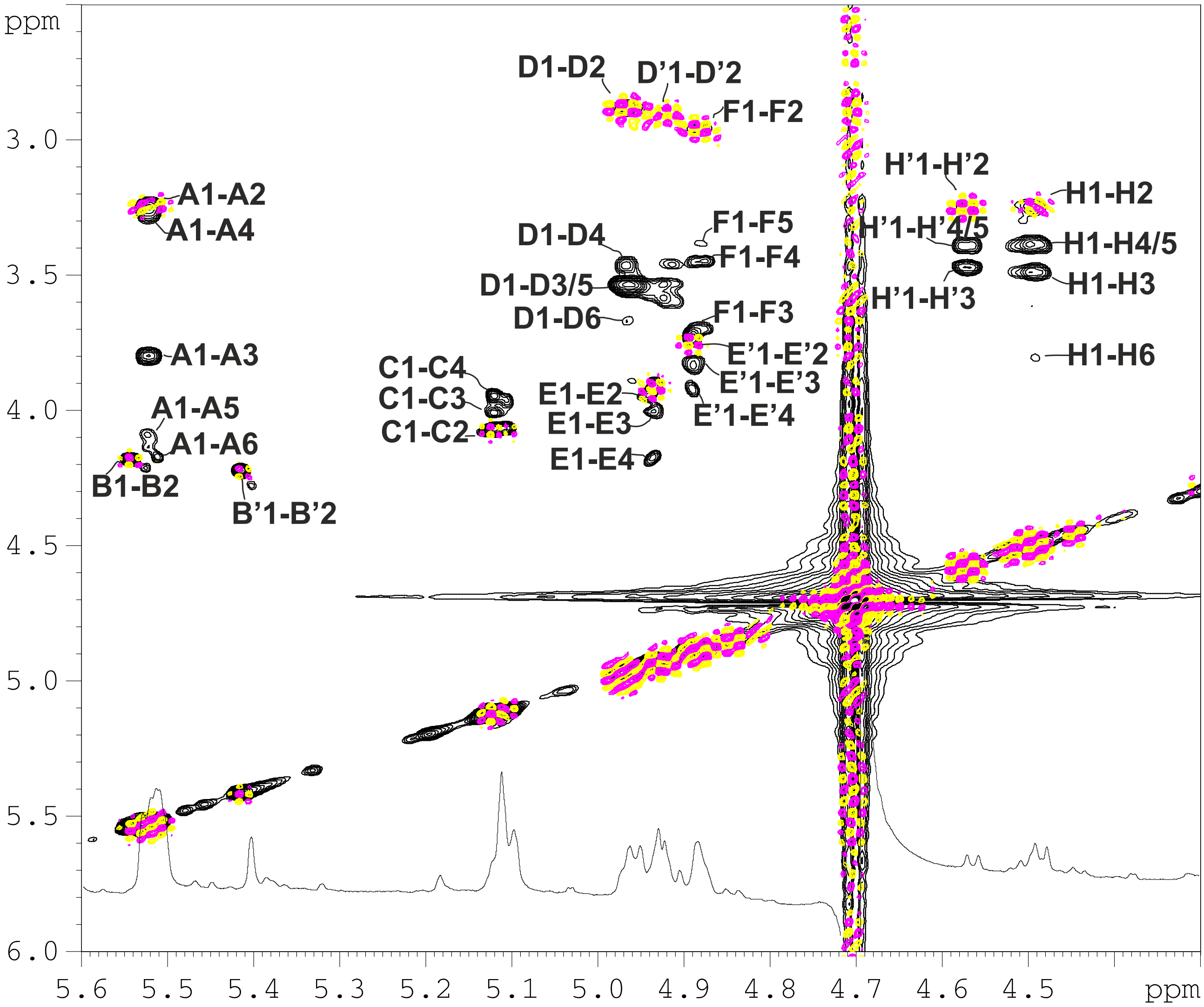

Figure S4. (600 MHz, 298 K, D_2O, Table S1). Zoom of the overlapped ^1H, COSY (yellow and pink) and TOCSY (black) spectra of the core OS of *B. stercoris*. Numbering of sugar units is as reported in Table S1.

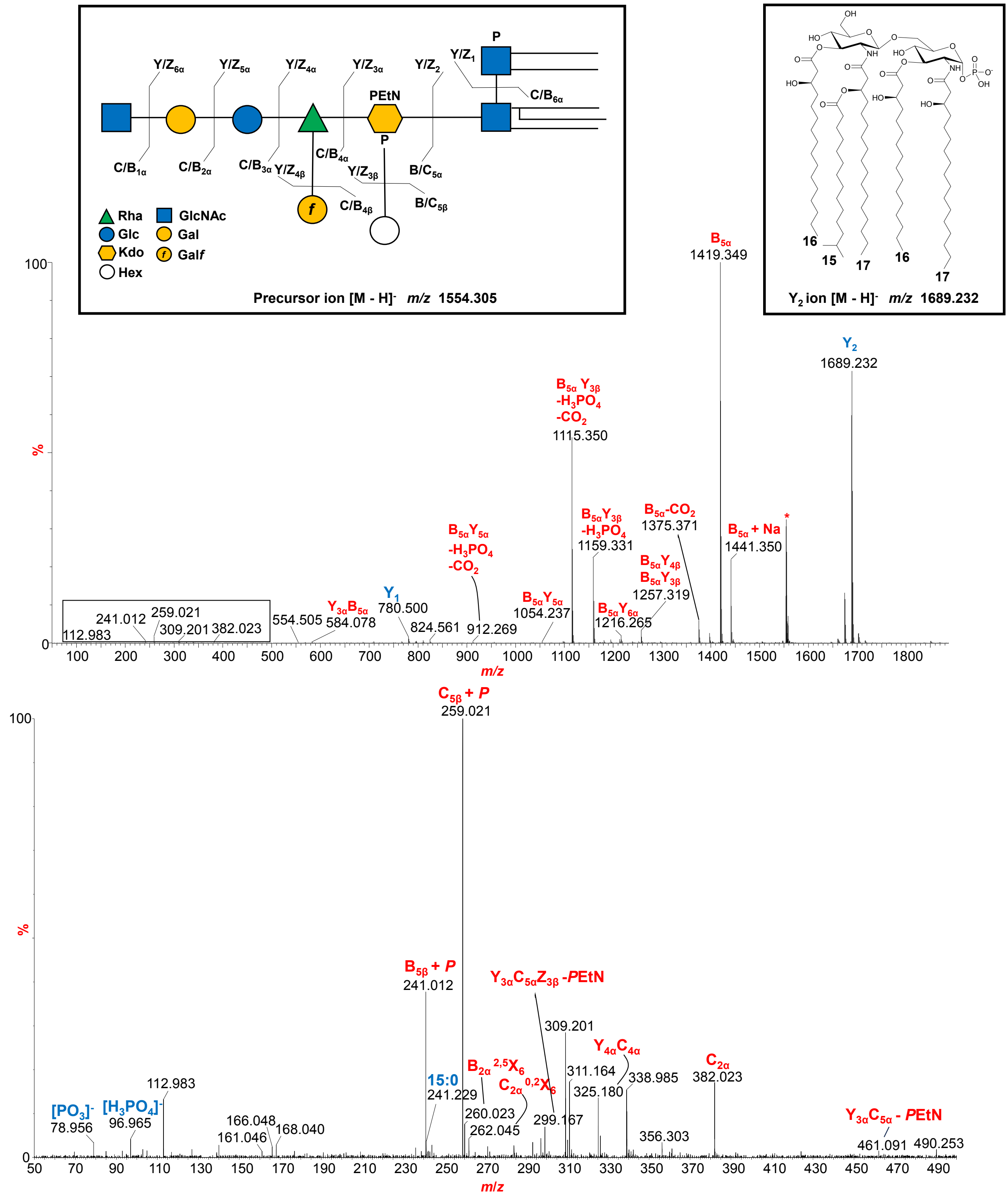

Figure S5. (Top) Expanded view of the negative-ion MS/MS spectrum of the $[M{-}2H]^{2-}$ precursor ion at *m/z* 1544.305 (3109.640 Da), corresponding to an R-LPS composed of a *mono*-phosphorylated, penta-acylated lipid A (*m/z* 1689.232, Y_2 fragment ion) linked to the core OS (*m/z* 1419.349, $B_{5\alpha}$ fragment ion). (Bottom) Enlarged view of the *m/z* 50-490 region. The spectra arise from fragmentation of the R-LPS, generating ions derived from the core OS, lipid A, and hybrid species containing both lipid and glycan moieties. Fragment ions associated with lipid A are shown in blue, whereas those originating from the saccharide portion are indicated in red. The proposed R-LPS structure is represented in the inset according to the Symbol Nomenclature for Glycans (SNFG) along with the proposed lipid A structure. The nomenclature of glycan fragment ions resulting from glycosidic bond cleavage is also indicated. *P* and *P*EtN denote phosphate and 2-aminoethyl phosphate, respectively.

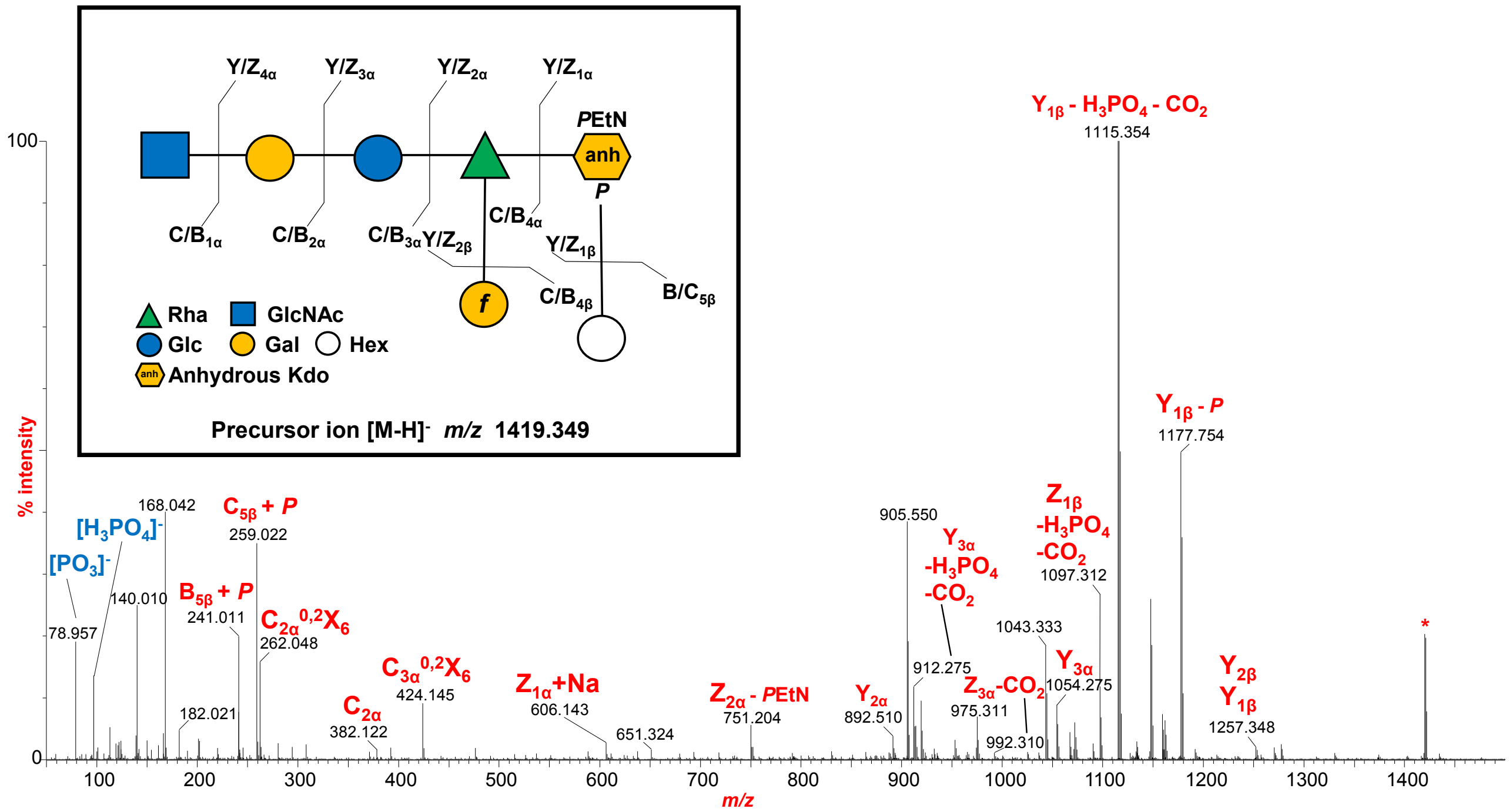

Figure S6. Negative-ion MS/MS spectrum of the $[M–H]^-$ precursor ion at *m/z* 1419.349 (1420.382 Da), corresponding to the core OS. The proposed core OS structure is represented in the inset according to the Symbol Nomenclature for Glycans (SNFG). The nomenclature of glycan fragment ions resulting from glycosidic bond cleavage is also indicated. *P* and *P*EtN denote phosphate and 2-aminoethyl phosphate, respectively.

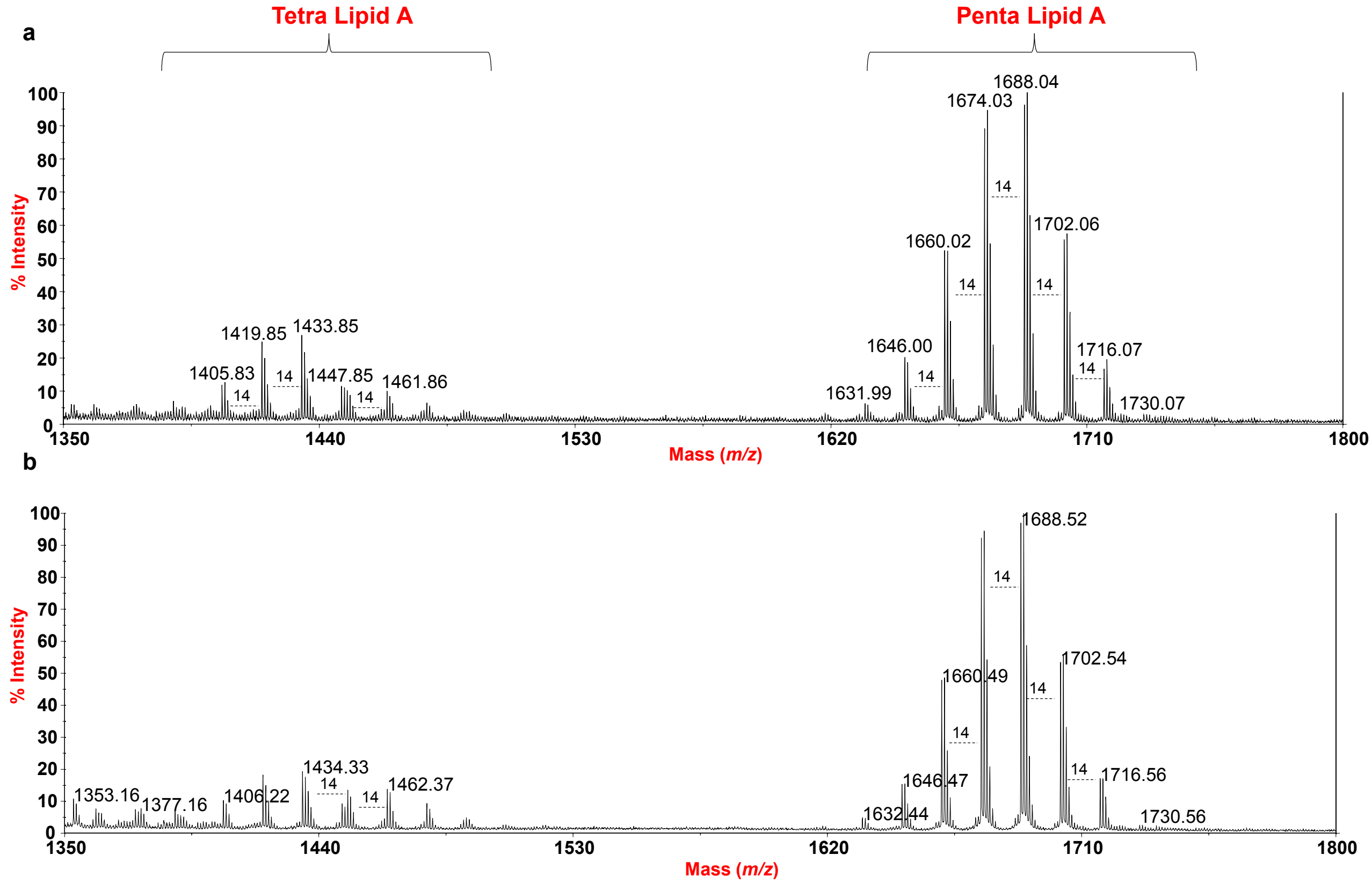

Figure S7. Reflectron MALDI-TOF MS spectra recorded in negative-ion polarity of the lipid A from *B. stercoris* R-LPS obtained through mild acid hydrolysis (**a**) and from bacterial pellet (**b**). Lipid A species are labeled as Tetra Lipid A and Penta Lipid A on the basis of the degree of acylation.

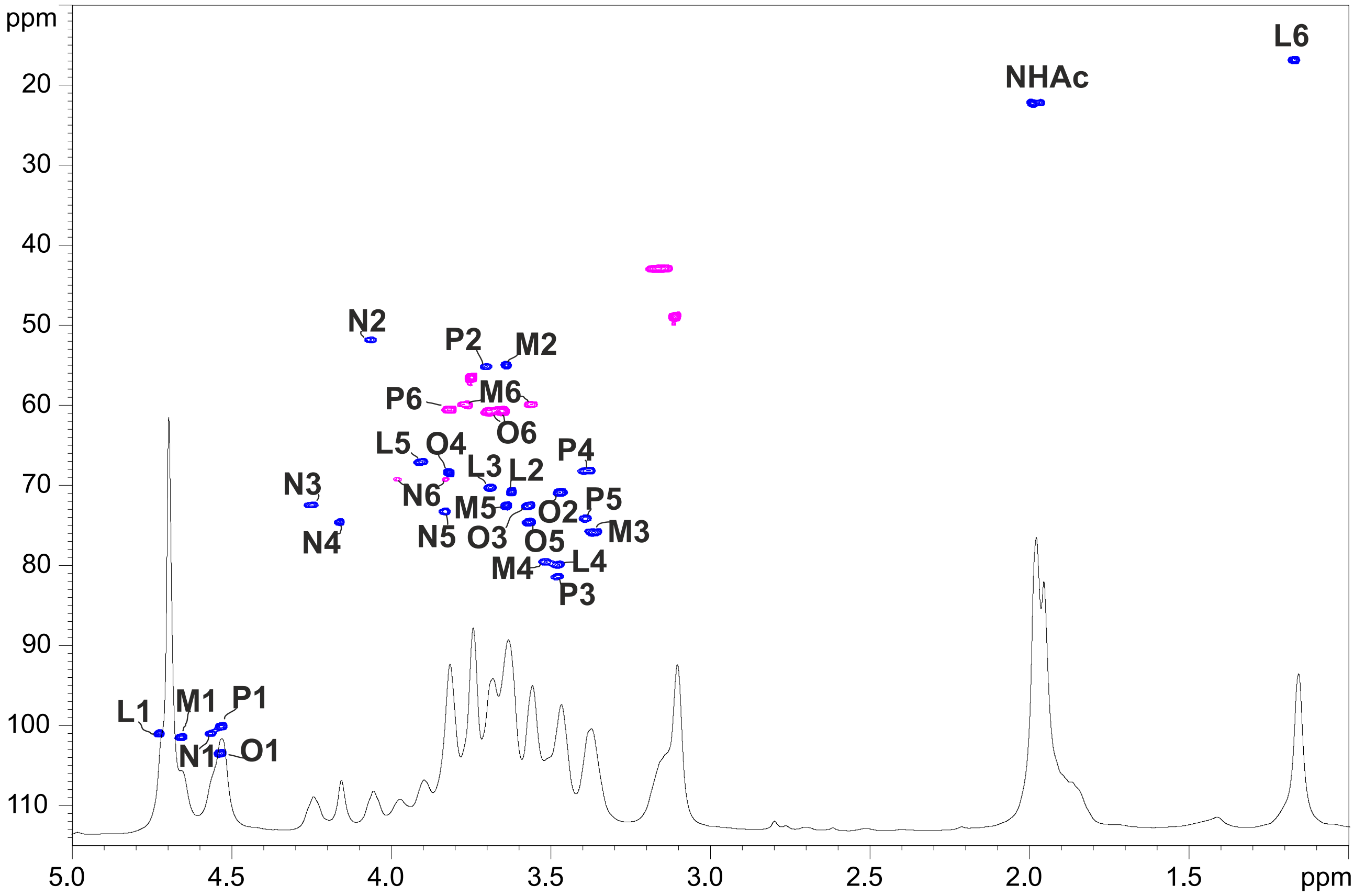

Figure S8. (600 MHz, 298 K, D_2O, Table S3). Superimposed ^1H and $^1H,^{13}C$ HSQC (blue and pink) spectra of the CPS from *B. stercoris*. Unassigned signals are attributed to contaminants not associated with the CPS.

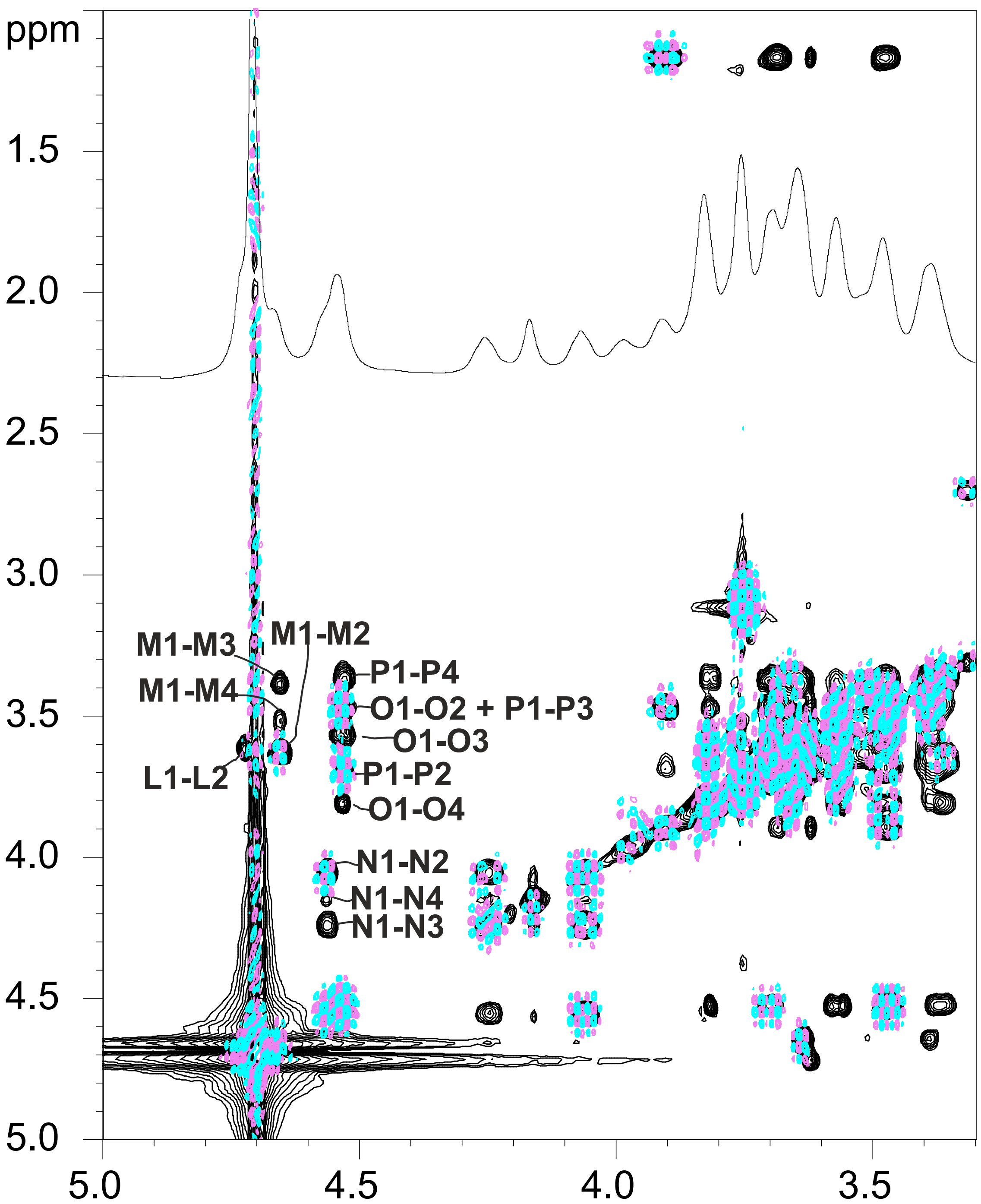

Figure S9. (600 MHz, 298 K, D_2O, Table S4). Zoom of the overlapped ^1H, COSY (light blue and pink) and TOCSY (black) spectra of the CPS of *B. stercoris*. Numbering of sugar units is as reported in Table S4.

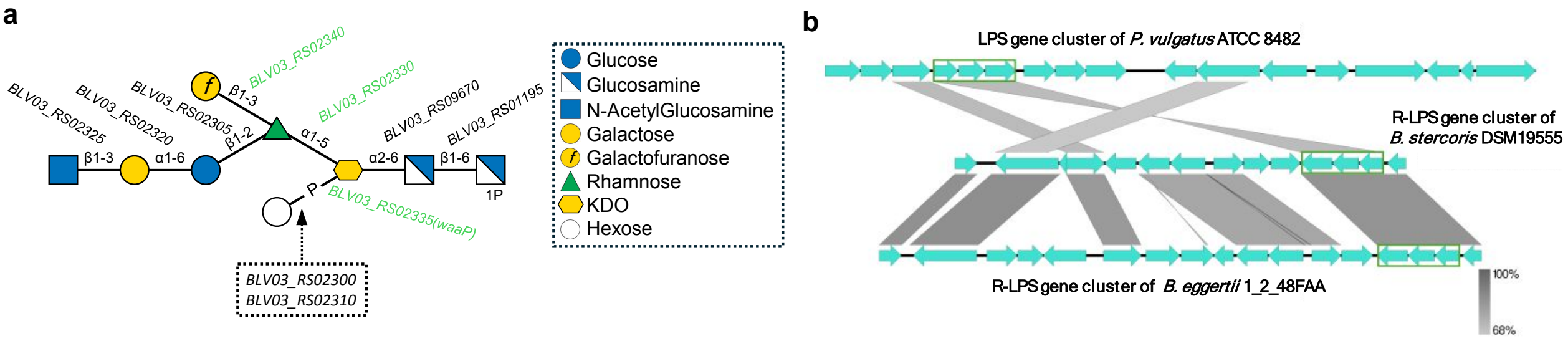

c

Locus tag	COGgene	PFAM	CAZy family	Function
BLV03_RS02290	COG1132	PF00664 PF00005	-	ATP-binding cassette, subfamily B, bacterial MsbA [EC:7.5.2.6]
BLV03_RS16765	-	-	-	hypothetical protein
BLV03_RS02295	COG0562	PF13450 PF03275	-	UDP-galactopyranose mutase [EC:5.4.99.9]
BLV03_RS02300	COG0463	PF00535	GT2	glycosyltransferase [EC:2.4.-.-]
BLV03_RS02305	COG1442	PF01501	GT8	UDP-glucose:(galactosyl)LPS alpha-1,2-glucosyltransferase [EC:2.4.1.58]
BLV03_RS02310	-	-	GT90	glycosyltransferase [EC:2.4.-.-]
BLV03_RS02315	-	PF01531	GT11	alpha-1,2-fucosyltransferase [EC:2.4.1.-]
BLV03_RS02320	-	PF03414	GT6	glycosyltransferase 6 domain-containing protein 1 [EC:2.4.1.-]
BLV03_RS02325	-	PF05704	GT32	glycosyltransferase [EC:2.4.-.-]
BLV03_RS02330	COG1216	PF00535	GT2	rhamnopyranosyl-N-acetylglucosaminyl-diphospho-decaprenol beta-1,3/1,4-galactofuranosyltransferase [EC:2.4.1.287]
BLV03_RS02335	ROG2301	PF06293.17	-	3-deoxy-D-manno-octulosonic acid kinase [EC:2.7.1.166]
BLV03_RS02340	COG1216	PF00535	GT2	glycosyltransferase [EC:2.4.-.-]

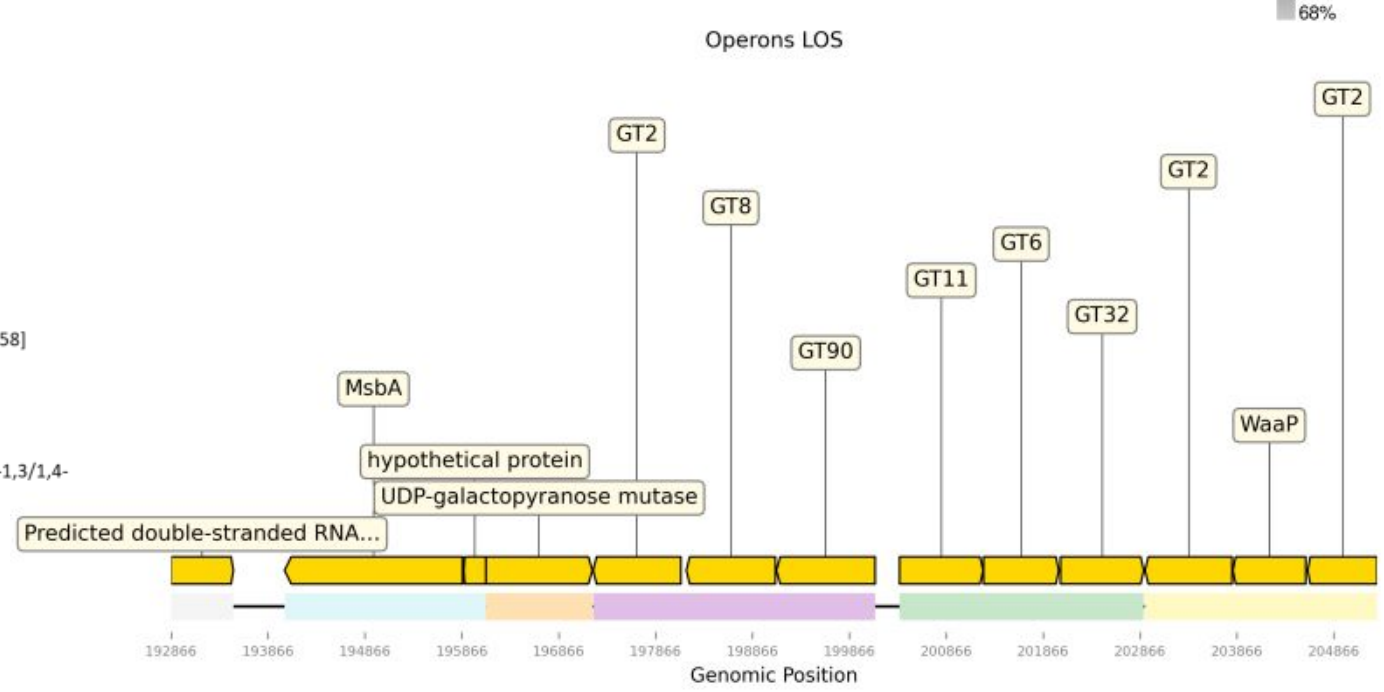

Figure S10. Bioinformatic prediction of the putative core OS biosynthetic loci of *B. stercoris* DSM 19555 and comparison to the putative LPS biosynthetic loci of structurally defined *Bacteroides* species. (**a**) Schematic representation of deacylated *B. stercoris* R-LPS according to the Symbol Nomenclature for Glycans (SNFG). Genes hypothesized to be involved in the addition of each sugar are indicated above the corresponding glycosidic linkage. Genes forming a conserved operon (sequence identity ≥ 68%) shared with *B. eggerthii* and *P. vulgatus*, including two GT2 glycosyltransferases and a *waaP* homolog, are highlighted in green; these are proposed to mediate the addition of galactofuranose, rhamnose, and phosphorylation of Kdo (green boxes in panel **b**). (**b**) Comparative alignment of the predicted LPS biosynthetic loci of *B. stercoris*, *B. eggerthii*, and *P. vulgatus*, generated by BLASTn and visualized with Easyfig 2.2.5. Genes are represented as arrows, and sequence similarity is indicated by grey shading. (**c**) Functional annotation of the genes within the locus, including predicted COG, Pfam, CAZy classification, and proposed function. On the right, a schematic overview of the predicted operon organization involved in *B. stercoris* core OS biosynthesis is shown. Genomic coordinates are reported below the diagram; individual operons are color-coded, and coding sequences are represented in yellow with brief functional annotations indicated above each gene.

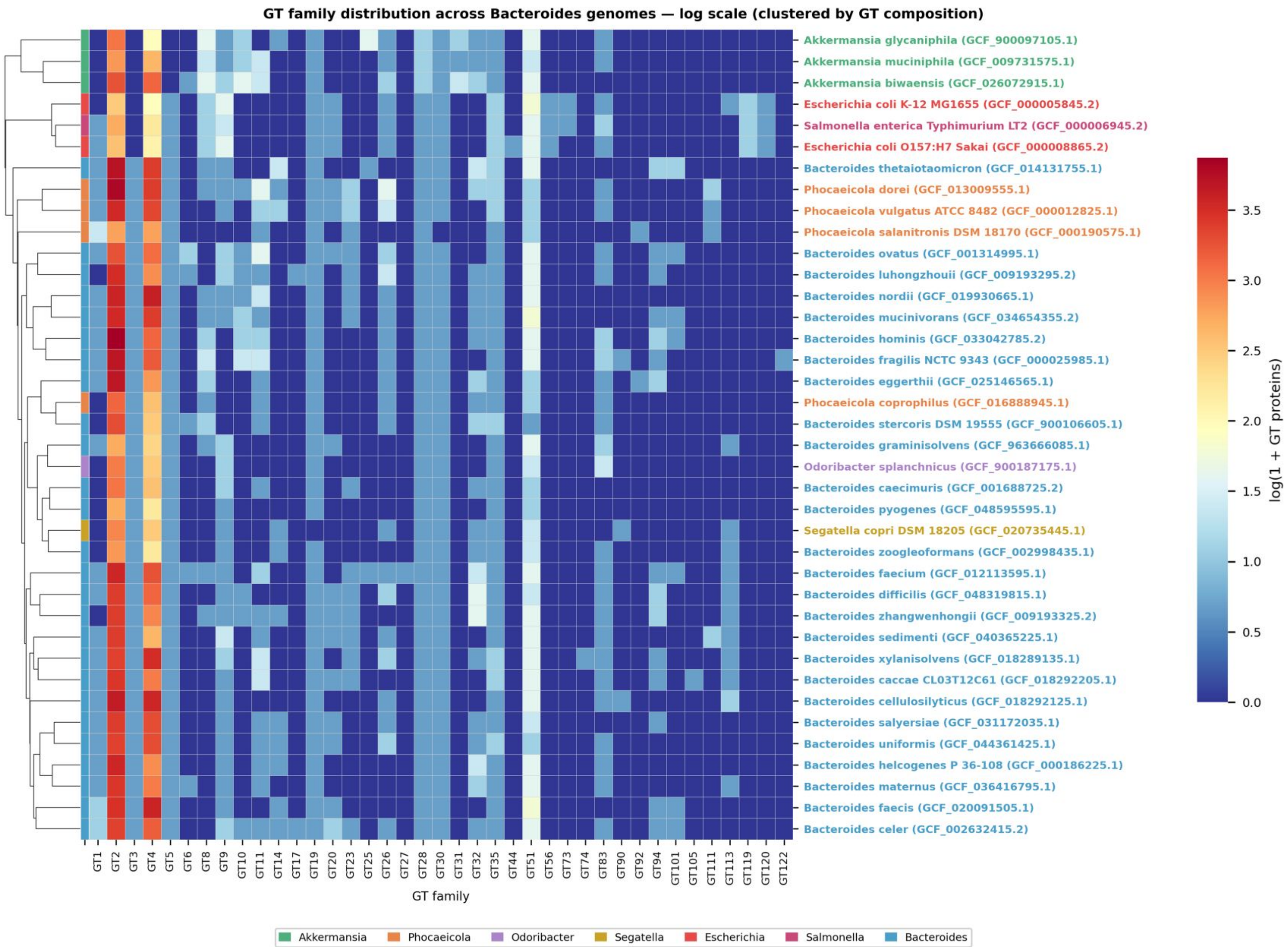

Figure S11. GT family distribution across representative Bacteroidales genomes. Heatmap showing log(1 + x)-transformed GT protein counts per family for each genome. Genomes are hierarchically clustered by GT composition using Euclidean distance and UPGMA linkage; GT families are ordered numerically. Genus-level taxonomy is indicated by the color bar.

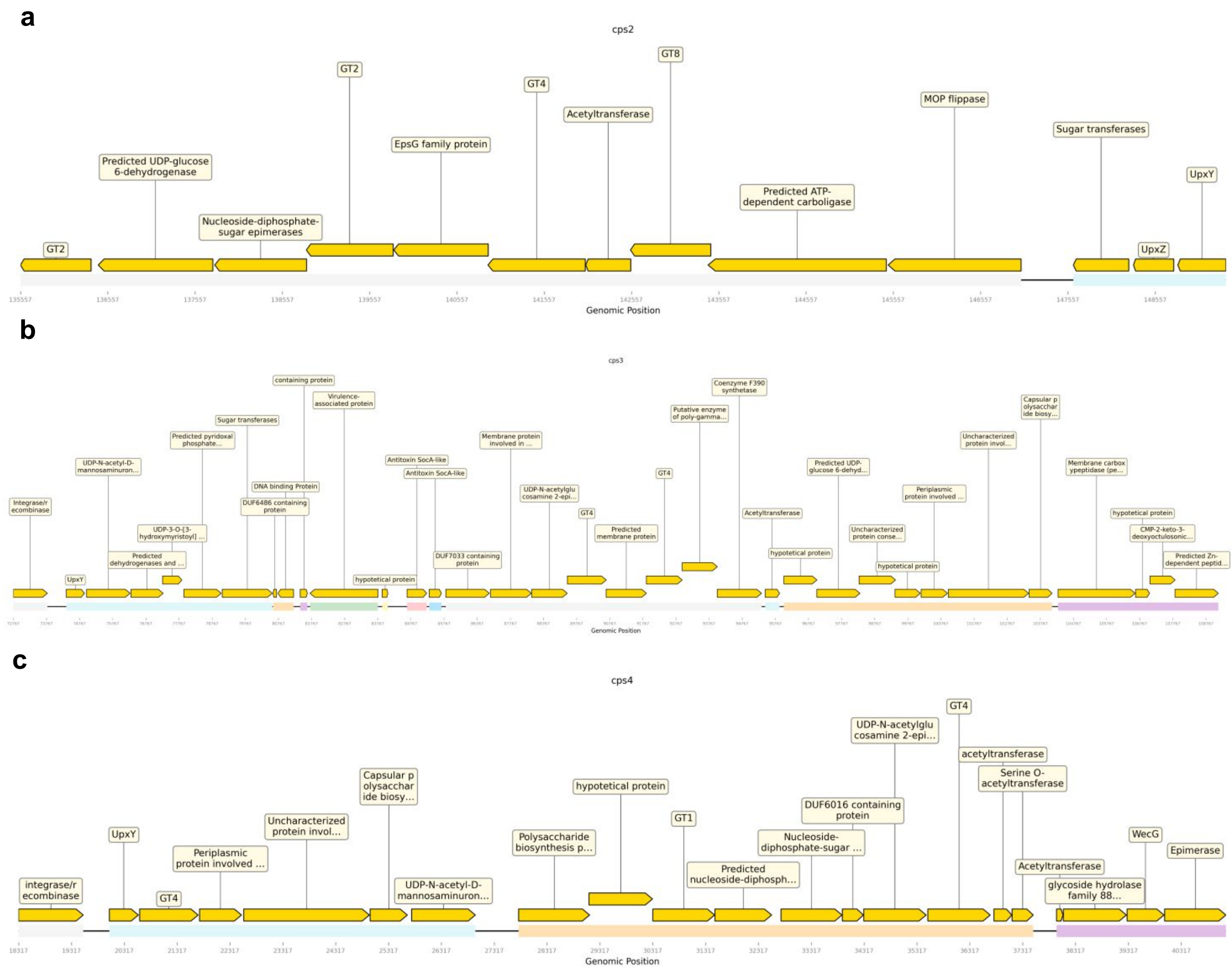

Figure S12. Bioinformatic identification of putative CPS biosynthetic loci of *B. stercoris* DSM 19555. Genomic coordinates (bp) are indicated below each locus. Predicted operons are distinguished by different background colors. Protein-coding genes are represented as yellow arrows indicating transcriptional orientation, with functional annotations reported above each gene. (**a**) cps2 biosynthetic locus. (**b**) cps3 biosynthetic locus. (**c**) cps4 biosynthetic locus.

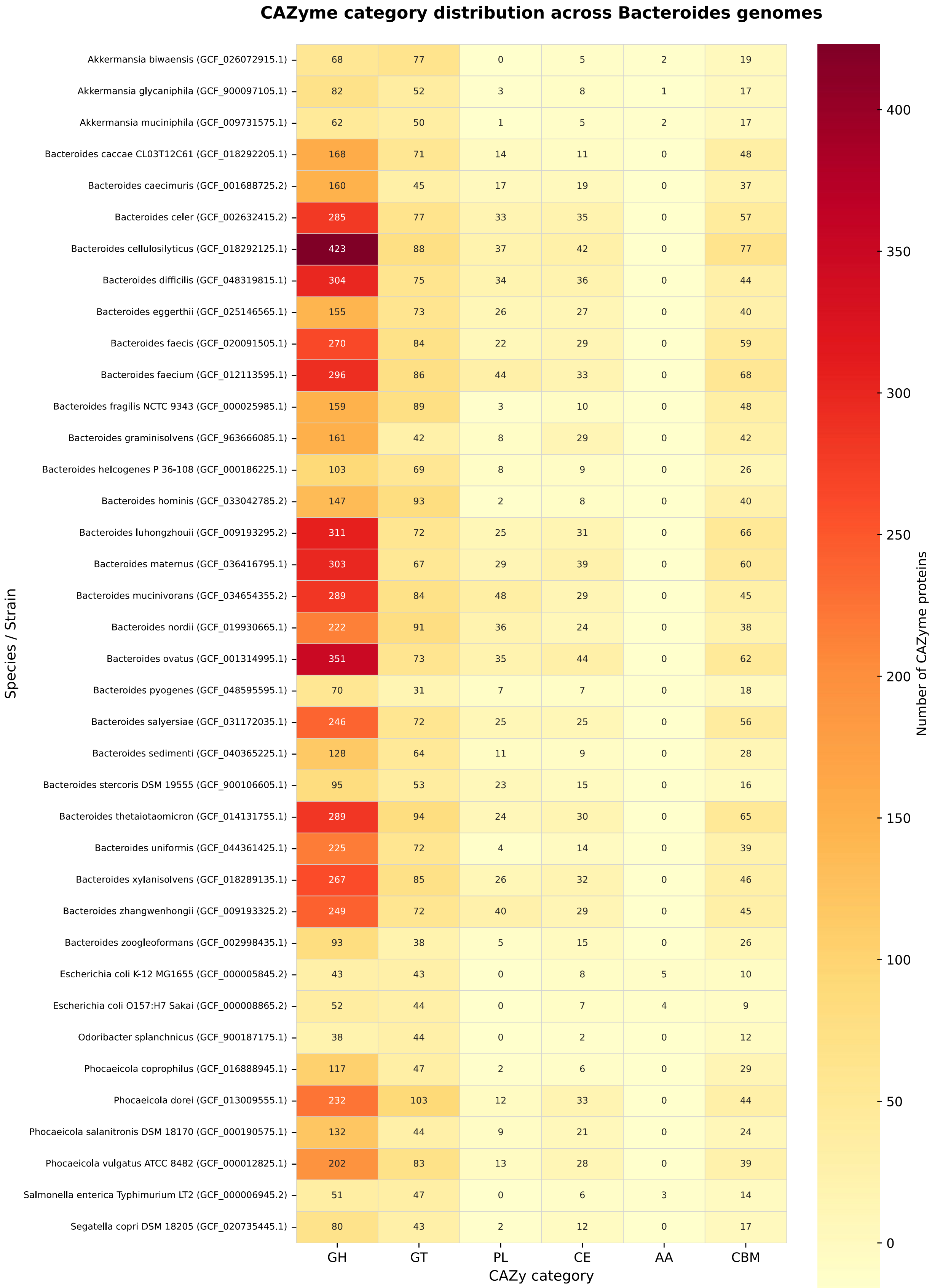

Figure S13. CAZyme category distribution across representative Bacteroidales genomes. Raw counts of predicted CAZyme proteins per category (GH, glycoside hydrolases; GT, glycosyltransferases; PL, polysaccharide lyases; CE, carbohydrate esterases; AA, auxiliary activities; CBM, carbohydrate-binding modules) are shown for each genome. Annotations were performed with dbCAN3 (v5.2.8) retaining only predictions supported by at least two tools.

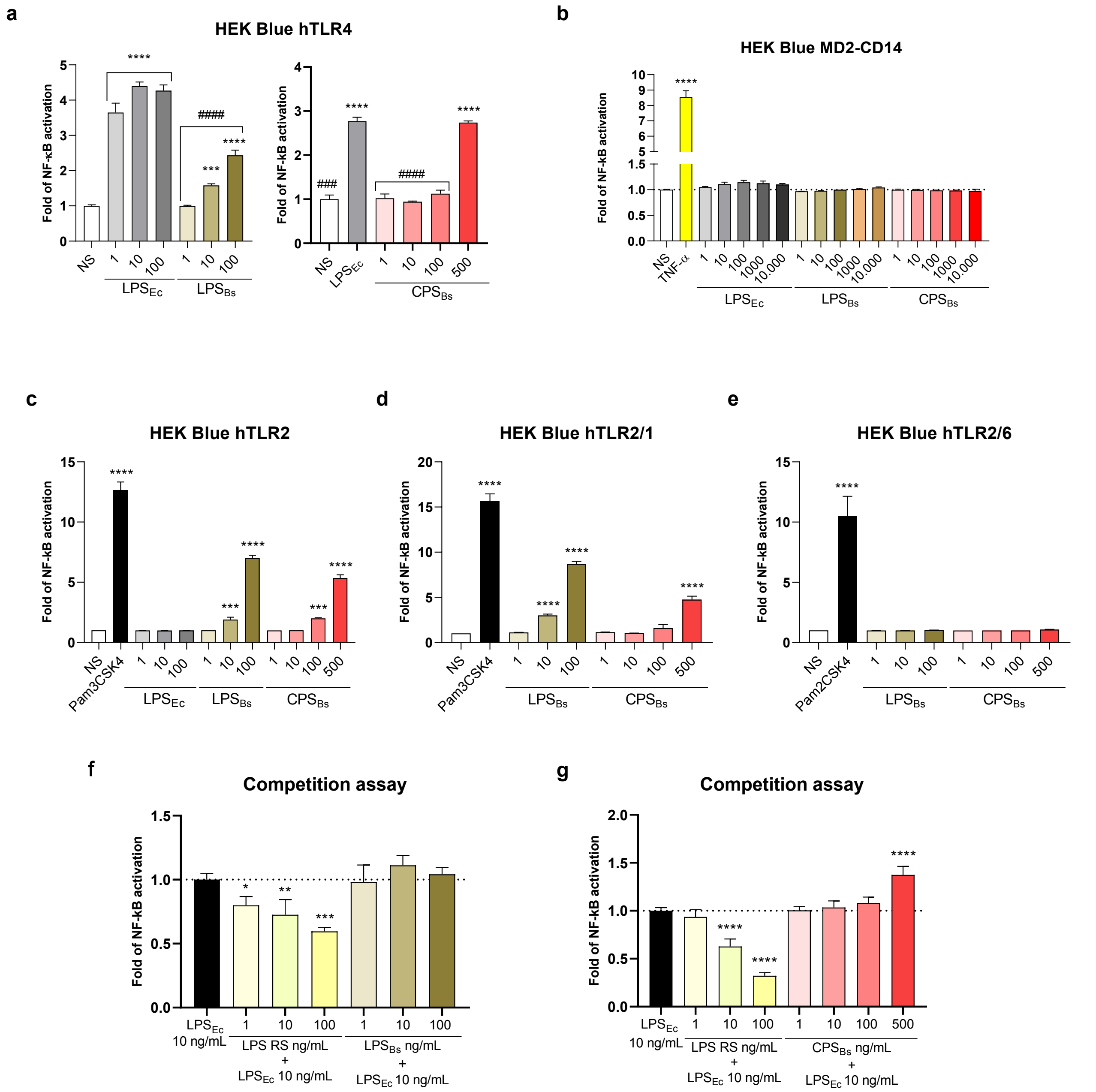

Figure S14. NF-κB activation in HEK-Blue TLRs cells stimulated with *B. stercoris* glycoconjugates. The effects of glycoconjugates were evaluated on HEK Blue™ TLR4 (**a**), HEK Blue™ MD2-CD14, HEK Blue™ TLR2, HEK Blue™ TLR2/1, and HEK Blue™ TLR2/6 (**c-d**). Cells were stimulated for 18 h with *E. coli* LPS (LPS_{Ec}), *B. stercoris* R-LPS (LPS_{Bs}), or CPS (CPS_{BSs}), and NF-κB activation was quantified by Quanti-Blue assay (OD 620 nm). LPS_{Ec} served as a positive control for TLR4 activation, while PAM3CSK4 (500 ng mL^{-1}) and PAM2CSK4 (500 ng mL^{-1}) were used as positive controls for TLR2-TLR2/1 and TLR2/6 activation, respectively. TNFα was used as positive control for HEK Blue MD2-CD14. Unstimulated (NS) cells were used as negative control. (**f-g**) Competition assay in HEK-Blue hTLR4 cells. Cells were pre-treated for 4 h with *R. sphaeroides* (RS) LPS (LPS RS), LPS_{Bs} (1-100 ng mL^{-1}), or CPS_{Bs} (1-500 ng mL^{-1}), followed by stimulation with 10 ng mL^{-1} LPS_{Ec} for 16 h. Data are presented as mean ± standard deviation. Statistical significance was determined by one-way ANOVA ($^{***}p < 0.001$; $^{****}p < 0.0001$ vs NS; $^{###}p < 0.001$; $^{####}p < 0.0001$ vs LPS_{Ec} for **a-e**, and $*p < 0.05$; $**p < 0.01$; $***p < 0.001$; $****p < 0.0001$ vs LPS_{Ec} 10 ng mL^{-1}).

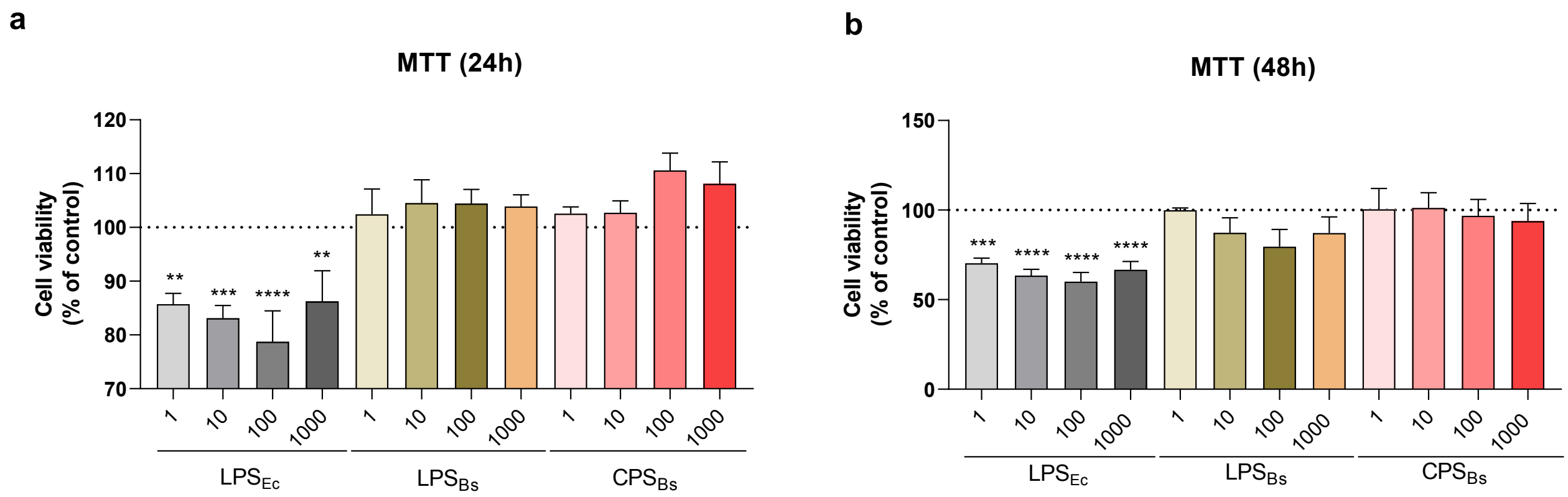

Figure S15. Cell viability assessed by MTT assay. THP-1 derived macrophages were treated for 24 h (**a**) or 48 h (**b**) with *E. coli* LPS (LPS_{Ec}), *B. stercoris* R-LPS (LPS_{Bs}), or *B. stercoris* CPS (CPS_{Bs}) at 1, 10, 100 or 1000 ng mL^{-1}. Absorbance at 570 nm was measured and normalized to the unstimulated control. Data are presented as mean ± s.d. Statistical significance was determined by one-way ANOVA against the control group (**$p < 0.01$; ***$p < 0.001$; ****$p < 0.0001$)

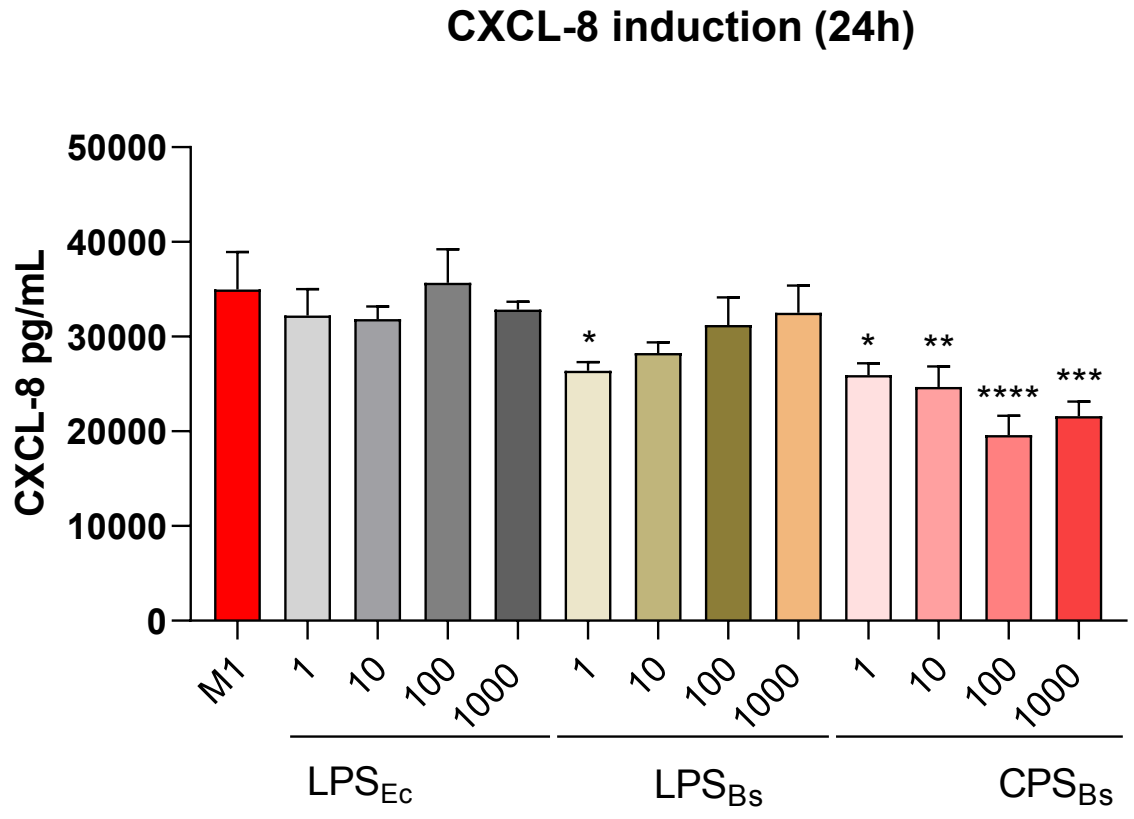

Figure S16. Stimulation of THP1 derived M(INFγ + LPS) macrophages. CXCL8 secretion by M(INFγ + LPS) macrophages after 24 h of stimulation. Data are presented as mean ± s.d. Statistical significance was determined by one-way ANOVA (**p* < 0.05; ***p* < 0.01; ****p* < 0.001; *****p* < 0.0001 vs. M1).

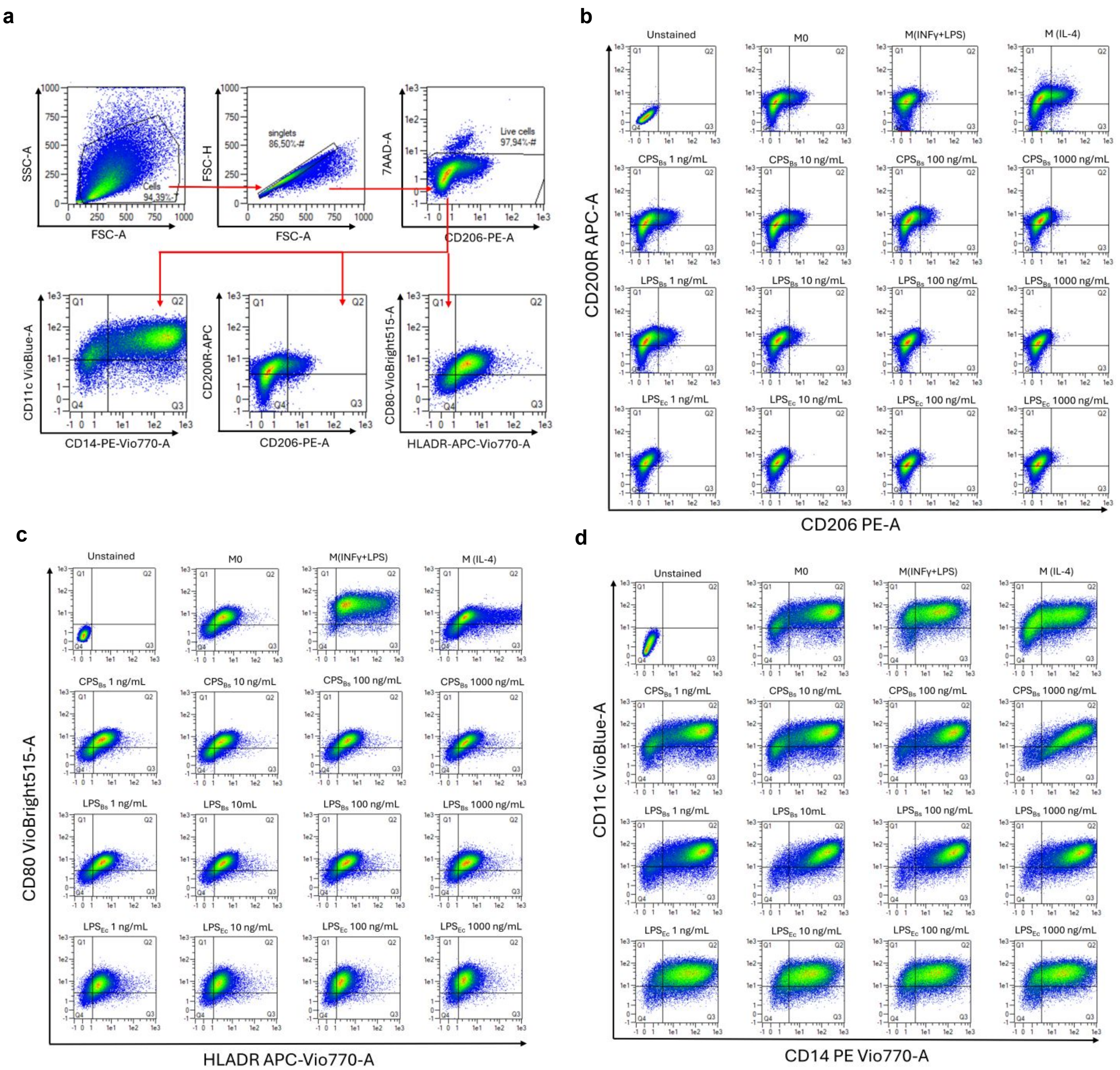

Figure S17. Visualization of the Gating Strategy applied. (**a**) Representative plots showing gating strategy used. Cells were identified by gating on live single cells. (**b-d**) Representative dot plot for all markers evaluated in the study, gating of positive cells was defined using unstained controls.

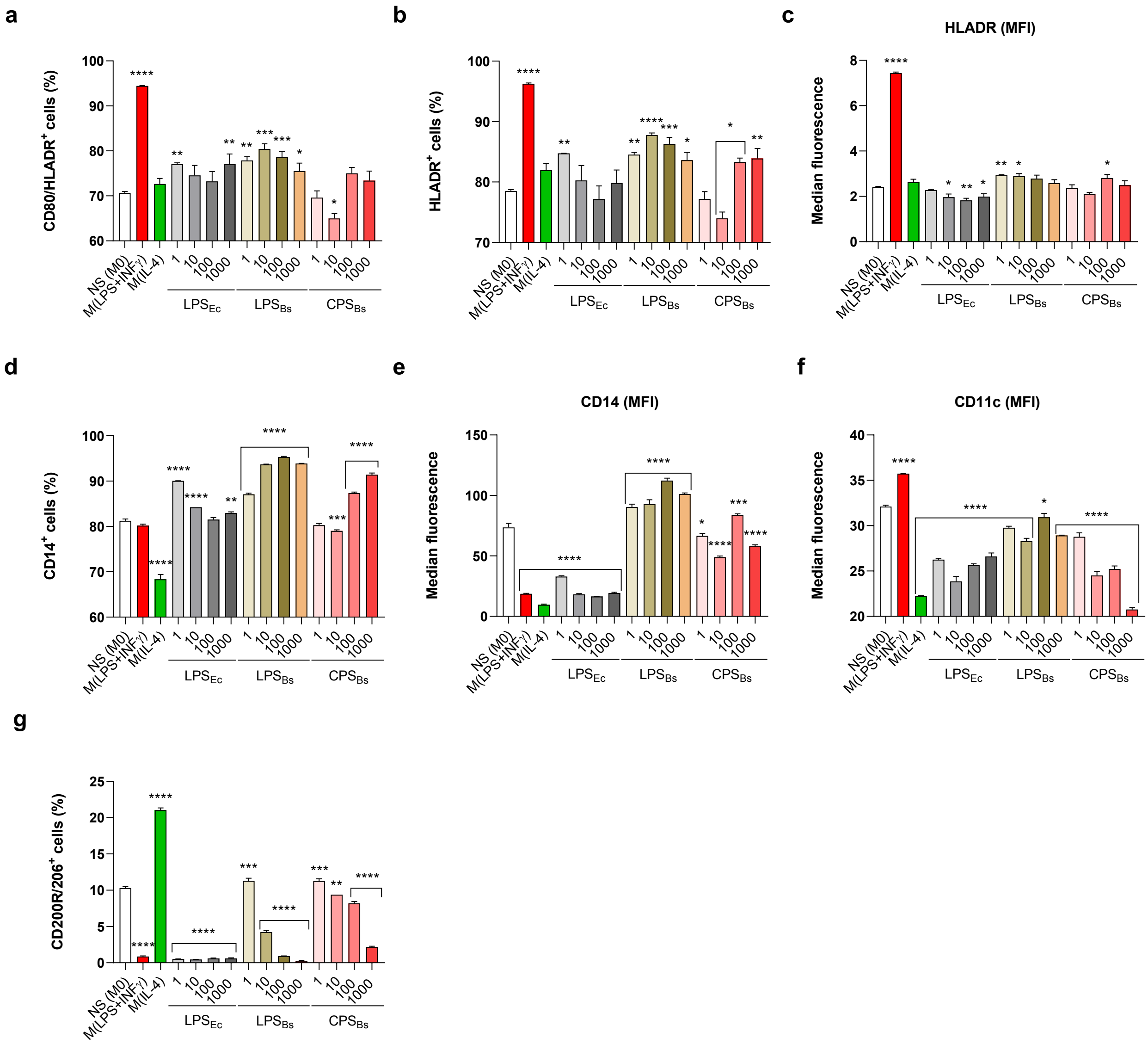

Figure S18. Flow cytometric analysis of THP1 derived macrophages M0 activation and polarization upon bacterial glycoconjugates stimulation. Cells were treated with *E. coli* LPS (LPS_{Ec}), *B. stercoris* R-LPS (LPS_{Bs}), or CPS (CPS_{BSs}) at increasing concentrations. Untreated cells (M0), M(INFγ + LPS) macrophages and M(IL-4) macrophages served as controls for different macrophages polarization state. (**a-c**) Percentage of $CD80^{+}/HLADR^{+}$ positive cells (**a**), $HLADR^{+}$ positive cells (**b**), median fluorescence intensity of $HLADR^{+}$ cells. Percentage of $CD14^{+}$ cells (**d**), median fluorescence intensity of $CD14^{+}$ (**e**) and $CD11c^{+}$ cells (**f**). Percentage of $CD200R^{+}/CD206^{+}$ positive cells (**g**). Statistical analysis was performed using one-way ANOVA with Dunnett's correction confronting each treatment with unstimulated M0 macrophages. *$p < 0.05$; **$p < 0.01$; ***$p < 0.001$; ****$p < 0.0001$.

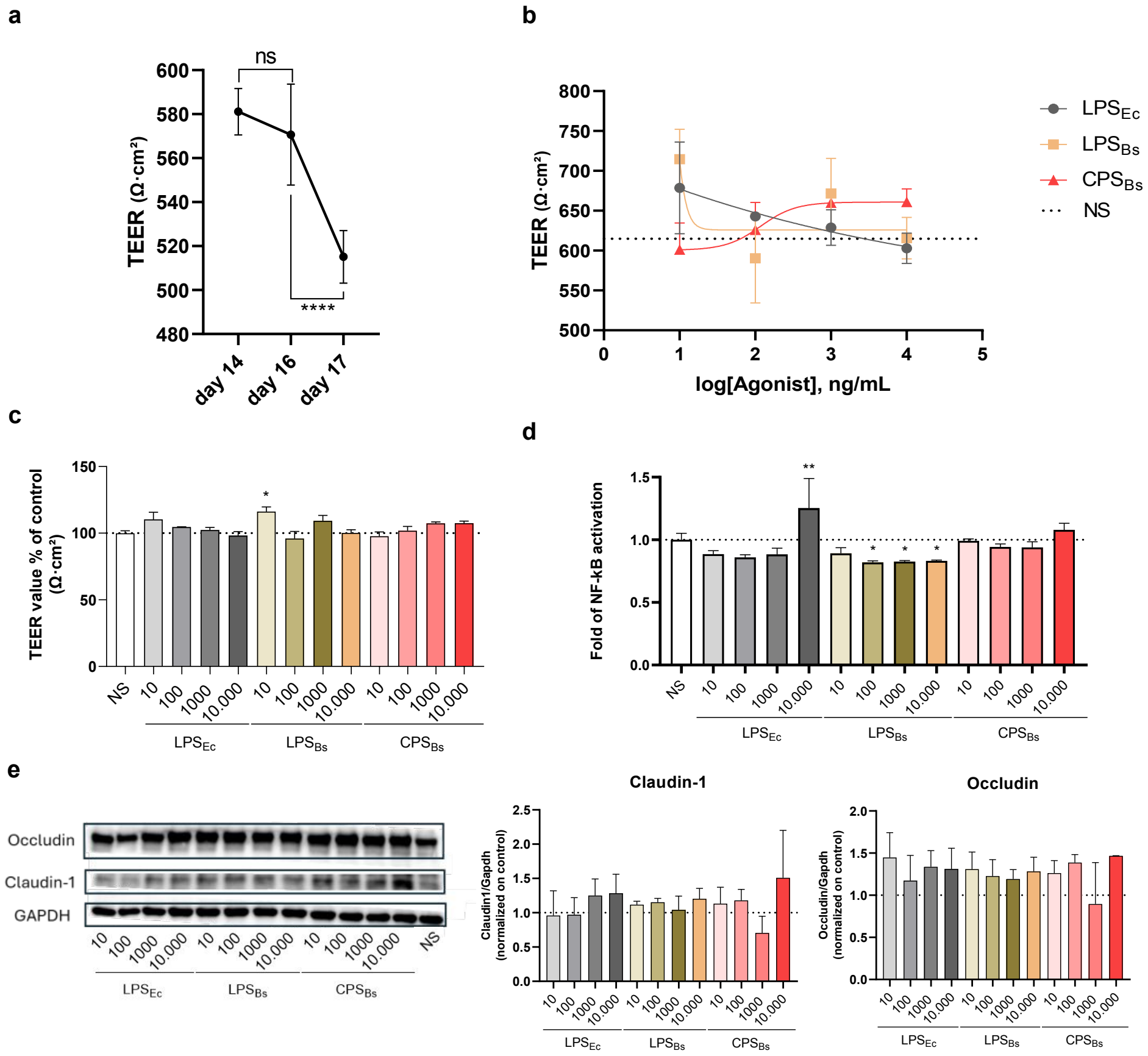

Figure S19. Effects of *B. stercoris* glycoconjugates on intestinal epithelial barrier function and tight junction expression. (**a**) TEER values of Caco-2 epithelial monolayers measured at different time points before (day 14-16) and after coculture with M(INFγ + LPS) macrophages. TEER values (**b**) and normalized TEER values (**c**) of Caco-2 epithelial monolayers after 18 h hours with increasing concentration of *E. coli* LPS (LPS_{Ec}), *B. stercoris* R-LPS (LPS_{Bs}) or *B. stercoris* CPS (CPS_{Bs}). (**d**) NF-κB activation of M(INFγ + LPS) macrophages in the basolateral compartment of the co-culture system upon stimulation with the different conditions. (**e**) Representative Western blot of tight junction proteins; Densitometric quantification of claudin-1 and occludin protein. Western blots were performed in triplicate. Data are presented as mean ± s.d. for all assays. Western blot densitometric analyses are reported as mean ± SEM. Statistical significance was assessed by one-way ANOVA.

Supporting notes

Supporting note 1

Structural characterization of the R-LPS from *B. stercoris* DSM 19555

Analysis of fully deacylated R-LPS revealed nine main spin systems corresponding to the monosaccharide residues of the R-LPS core oligosaccharide (core OS). All residues were identified in pyranose form based on their ^{13}C chemical shifts and diagnostic long-range HMBC correlations, with the exception of residue **C**, which was assigned as a galactofuranose unit on the basis of its characteristic low-field carbon signals and specific *intra*-residue correlations. The Kdo residue (**K**) was identified from its characteristic diastereotopic H-3 methylene signals (δ_H 2.02/1.82 ppm; δ_C 34.4 ppm; Figures. 1a, S1-S2, and Table S1). Its α-anomeric configuration was established from the chemical shift of H-3 and the coupling constants $^3J_{H7,H8a}$ and $^3J_{H7,H8b}$.[1] ^{31}P,^{1}H HSQC experiments (Figure S3) revealed the occurrence of three phosphate groups. One phosphate signal (δ_P 0.41 ppm) correlated with H-7 of **K** and with methylene protons at δ_H 3.92 and 3.15 ppm thus indicating the occurrence of a 2-aminoethyl phosphate (*P*EtN) unit at this position, while a second phosphate signal (δ_P 1.35 ppm) correlated with H-4 of **K** (δ_H 4.15 ppm), indicating phosphorylation at this position. A third phosphate (δ_P 1.84 ppm) correlated with anomeric signal of residue **A** (Figure S3). The non-stoichiometric phosphorylation of **K** resulted in signal splitting for residues **B**, **D** and **H**, generating additional spin systems **B'**, **D'**, and **H'**. Residues **B**/**B'** were assigned as α-rhamnopyranose (α-L-Rha*p*) units based on scalar correlations extending from H-2 to the H-6 methyl group in the TOCSY spectrum. The *manno*-configuration was supported by $^3J_{H1,H2}$ and $^3J_{H2,H3}$ values below 2 Hz, indicative of an equatorial H-2, while the α-anomeric configuration was confirmed by the $^1J_{H1,C1}$ coupling constant. Residues **A**, **D/D'**, **F** and **H/H'** were all assigned to *gluco*-configured sugars based on their large $^3J_{H,H}$ ring proton values and the chemical shift values, in agreement with *gluco*-configuration of pyranose rings. Residues **A** and **D/D'** were identified as the α- and β-glucosamine (D-GlcN) units of the lipid A backbone. Their assignment was supported by correlations of the H-2 protons with nitrogen-bearing carbon signals at δ_C 54.3 and 55.6 ppm (Table S1, Figure 1a), as well as by an *inter*-residual NOE between H-1 of residues **D/D'** and the H-6 methylene protons of residue **A** (Figure 1b). Residue **F** was assigned as β-D-Glc*p*N based on the correlation of its anomeric proton with a carbon resonance at 56.1 ppm, while residues **H/H'** were identified as β-glucose units. Spin systems **C** and **E/E'** were assigned to *galacto*-configured sugars. Residue **C** was confirmed as a β-galactofuranose (β-D-Gal*f*), as described above, whereas residues **E/E'** were identified as α-galactopyranose (α-D-Gal*p*) units based on characteristic $^3J_{H3,H4}$ and $^3J_{H4,H5}$ values (approximately 3 Hz and <1 Hz, respectively), TOCSY correlations up to H-4, diagnostic $^3J_{H1,H2}$ and $^1J_{C,H}$ coupling constants, and *intra*-residue NOE contacts between H-1 and H-2. Downfield-shifted carbon resonances indicated glycosylation at O-6 of residues **A**, **D/D'**, and **H/H'**, at O-2 and O-3 of **B/B'**, at O-3 of **E**, and at O-5 of **K**. In contrast, residues **E'**, **F**, and **C** were identified as terminal units, consistent with the linkage analysis data. The complete core OS sequence was established by combining *inter*-residue NOE contacts observed in the ROESY spectrum (Figure 1b) with long-range HMBC correlations. Starting from the lipid A disaccharide backbone, composed of a β-(1→6)-linked GlcN disaccharide (**A** and **D/D'**), residue **D/D'** was substituted at O-6 by the Kdo unit (**K**), as supported by the weak downfield shift of C-6 of residue **D/D'** (δ 62.5 ppm, Table S1), characteristic of an α-(2→6) ketosidic linkage between Kdo and the β-GlcN of lipid A. The Kdo residue was further substituted at O-5 by α-L-Rha (**B**/**B'**), as demonstrated by the NOE contact between H-1 (**B**/**B′**) and H-5 (**K**) (Figure 1b). Residue **B**/**B'** was in turn substituted at O-2 and O-3 by **H/H'** and **C**, respectively. This arrangement was confirmed by NOE correlations between H-1 of **H** and H-2 of **B**, H-1 of **H'** and H-2 of **B'**, and between H-1 of **C** and H-3 of **B/B'**. The β-D-Glc residue **H/H'** was further substituted at O-6 by α-D-Gal (**E/E'**), as evidenced by the NOE contact between the anomeric protons of **E/E'** and the H-6 methylene protons of **H/H'** (Figure 1b). Finally, a NOE correlation between H-1 of **F** and H-3 of **E** (and not of **E'**) established the linkage of β-D-GlcN **F** at O-3 of residue **E**, identifying it as the terminal sugar unit present as a non-stochiometric substituent. This structural assignment was further supported and complemented by NMR analysis of the fraction obtained through mild acid hydrolysis of an aliquot of the R-LPS (Figure S1), which also confirmed that the terminal β-D-GlcN residue **F** carries an *N*-acetyl group ($\delta_{H/C}$ 1.98/22.6 ppm). A detailed structural assessment of the R-LPS was further strengthened by ESI-MS and tandem MS analyses performed on the intact molecule (Figure 1c, S5 and S6). Direct infusion ESI-MS in

negative-ion mode revealed a heterogeneous ion distribution (Figure 1c), comprising signals attributable to fully assembled R-LPS species as well as diagnostic fragment ions derived from the lipid A. The negative-ion ESI-MS spectrum was dominated by an intense cluster of doubly charged ions in the *m/z* range 1519-1587, with a prominent $[M–2H]^{2−}$ species at *m/z* 1554.305 (3109.640 Da). This ion is consistent with an R-LPS structure composed of one Kdo, four hexoses (Hex), one deoxyhexose (dHex), one *N*-acetylhexosamine (HexNAc), one phosphate group, one *P*EtN, and a *mono*-phosphorylated penta-acylated lipid A carrying two 17:0(3-OH), two 16:0(3-OH), and one 15:0. The high structural variability in *B. stercoris* lipid A was proven by the presence of peaks differing by 14 amu, indicative of lipid A acyl chains of different length (Figure 1c), as also evidenced in the MALDI-TOF MS analysis executed on isolated lipid A fraction (Figure S9). Additional minor ion clusters were observed and corresponded to truncated R-LPS species lacking *P*EtN, HexNAc, one 16:0(3-OH), or both HexNAc and Hex residues, respectively. Negative-ion MS/MS analysis (Figure S5 and S6) was crucial to confirm the structure of the core OS deduced by NMR and to place the additional hexose not identified via NMR. The MS/MS spectra of the precursor ion at *m/z* 1554.305 displayed extensive fragmentation of the core OS following loss of the lipid A moiety. Among these, diagnostic fragments, including ions at *m/z* 461.091 and 584.078 were consistent with a *P*EtN-Kdo-*P*-Hex sequence at the proximal region of the core OS. This interpretation is reinforced by the ions at *m/z* 241.012 and 259.021, which are consistent with a *P*-Hex fragment. Overall, MS/MS analysis enabled the allocation of the additional hexose unit as a substituent of the Kdo to which it is linked via phosphodiester bridge at its O-4 position. The MS/MS spectrum of the ion at *m/z* 1419.349 (Figure S6), obtained combining a high cone voltage with a fixed collision energy, further supported the sugar sequence. The peaks at *m/z* 606.143, 751.204, and 892.510 once again confirmed the presence of the *P*EtN-Kdo-*P*-Hex moiety and the Kdo-dHex-Hex sequence.
As mentioned above, the lipid A moiety was characterized by analysing the corresponding signals detected in the ESI MS spectrum of the intact R-LPS, as well as by MALDI-TOF MS of the lipid A fraction obtained after mild acid hydrolysis (Figure S7a). As shown in Figure S7a, the lipid A displayed marked microheterogeneity, with tetra- and penta-acylated species detected in their *mono*-phosphorylated forms. Within each acylation class, additional variability was observed as recurring mass differences of 14 amu, corresponding to $–CH_2–$ units and thus reflecting differences in acyl chain length, in accordance with previous studies on *Bacteroides* lipid A.[2-4] To validate these assignments and rule out potential artifacts arising from the acid treatment, MALDI-TOF MS was also performed directly on intact bacterial cells. This analysis (Figure S7b) confirmed the presence of the same lipid A species, thereby strengthening the structural conclusions derived from the hydrolyzed samples. Therefore, NMR data, in conjugation with compositional analyses and MS data, showed that *B. stercoris* produces an R-LPS with the typical highly complex lipid A of *Bacteroides* and a peculiar core OS, decorated by *P*EtN and built up of one Kdo, one α-L-Rha, one β-D-Gal*f*, one β-D-Glc, one α-D-Gal, one non-stoichiometric β-D-GlcNAc, and an additional hexose, likely glucose, that is strikingly linked to Kdo via phosphodiester bridge (Figure 1d).

Supporting note 2

Structural characterization of the CPS from *B. stercoris* DSM 19555

The CPS was structurally characterized by NMR spectroscopy following the same approach described for R-LPS core OS (Table S4, Figures. S1, S8-S9). As shown in the ^{1}H (Figure S1) and ^{1}H, ^{13}C HSQC (Figure S8) spectra, five spin systems were recognised and all in their pyranose form. All spin systems, except for **L**, were identified as *galacto*-configured sugar units based on their low $^{3}J_{H-3,H-4}$ and $^{3}J_{H-4,H-5}$ values. While residue **O** was assigned as a galactose, **M**, **N** and **P** were attributed to amino galactoses as proven by the correlation of their H-2 with nitrogen-bearing carbon signals at δ_C 55.0, 52.0, and 55.2 ppm (Table S4), respectively. Interestingly, the chemical shift of H-2 and C-2 of **N** suggested the occurrence of an amine and not of an acetamido group at such a position.[5] By contrast, the presence of two *N*-acetyl groups was supported by methyl signals at δ_H 1.98 and 1.96 ppm (Figure S1), together with NOE correlations between H-2 of residues **M** and **P** and the corresponding acetyl methyl protons. The β-anomeric configuration was established for all *galacto*-configured residues (**M**, **O**, **P**) on the basis of the *intra*-residue NOE contacts of their H-1 with H-3 and H-5, and their $^{3}J_{H1,H2}$ and $^{1}J_{CH}$ values. Finally, spin system **L** was assigned as an α-Rha*p* residue, as described above,

with coupling constants indicative of a *manno*-configuration and α-anomeric configuration. Glycosylation shifts were observed for O-4 of **L** and **M**, for O-4 and O-6 for **N**, and for O-3 of **P**, while **O** was a terminal sugar unit. The primary sequence of the repeating unit of the CPS was inferred again by analysing both the HMBC correlations and the *inter*-residue NOE contacts (Figure 2a,b). Briefly, α-D-Gal*p*N **N** was substituted at O-4 and O-6 by β-Gal*p* **O** and β-D-Gal*p*NAc **P**, respectively, as demonstrated by the HMBC correlations between H-1 of **P** and C-6 of **N**, and between H-1 of **O** and C-4 of **N**. Residue **N** glycosylated position O-4 of β-D-Gal*p*NAc **M**, which in turn was connected at O-4 to α-L-Rha*p* **L**, as supported by the HMBC correlation between H-1 of **N** and C-4 of **M** and the strong NOE contact between H-1 of **M** and H-4 of **L**. Finally, α-L-Rha*p* **L** was found to be attached to residue **P** at O-3, as evidenced by the long-range correlation between H-1 of **L** and C-3 of **P** together with the corresponding *inter*-residue NOE contact between H-1 of **L** and H-3 of **P**. In conclusion, the repeating unit of the *B. stercoris* CPS was defined as a novel branched, positively charged pentasaccharide enriched in *galacto*-configured and amino sugars, as depicted in Figure 2c.

Supporting note 3

Bioinformatic analysis of putative genes encoding for *B. stercoris* DSM 19555 R-LPS biosynthesis

We performed an *in silico* analysis of *B. stercoris* DSM 19555 genome (GenBank accession GCF_900106605.1) to identify and annotate the putative biosynthetic loci responsible for R-LPS and CPS production. Gene function predictions were based on KEGG Orthology (KofamKOALA), InterPro domain annotation,[6] dbCAN3 for CAZyme classification[7,8] and BLAST-based homology searches against experimentally characterized enzymes from *B. thetaiotaomicron*, *Porphyromonas gingivalis* and *B. fragilis*. Operon boundaries together with COGs were predicted using Operon Mapper web server.[9] The predicted biosynthetic loci were visualized using DNA Features Viewer library and EasyFig.[10] A comparative GT family distribution across multiple Bacteroidales and outgroup genomes was computed using dbCAN3 and visualized as annotated heatmaps. Homologs of all core Raetz pathway enzymes were identified in the *B. stercoris* DSM 19555 genome (Table S5). Consistent with the penta-acylated lipid A structures determined by MALDI-TOF MS and ESI-MS, the genome encodes a single secondary acyltransferase (BLV03_RS00695) homologous to LpxL, while no LpxM homolog was detected. Two copies of *lpxA* were identified, a feature previously reported in gut-associated Bacteroidales and potentially contributing to the lipid A heterogeneity observed by MS.[11] The genome further encodes lipid A-modifying enzymes consistent with the structural features identified by NMR and MS. Two PAP2-family phosphatases were assigned as homologs of *B. thetaiotaomicron* LpxF and *B. fragilis* LpxE, accounting for the *mono*-phosphorylated lipid A backbone. In addition, homologs of PagL (3-*O*-deacylase) and EptB (*P*EtN transferases) were identified. In particular, EptB likely mediates the *P*EtN substitution at O-7 of Kdo, as supported by ^{1}H, ^{31}P HSQC data.

A ~12 kb genomic region (BLV03_RS02290-BLV03_RS02340) was identified as the putative locus for core OS biosynthesis (Figure S10). Within this region, a conserved inner core module was identified, including a *waaP*-like kinase (BLV03_RS02335), predicted to phosphorylate the Kdo residue, a GT2 rhamnosyltransferase (BLV03_RS02340), and another GT2 (BLV03_RS02330) that is predicted to be a β-1,3/1,4-galactofuranosyltransferase [EC:2.4.1.287; K16649], all organized within a predicted operon. This organization is consistent with the biosynthesis of the β-Gal*f*-(1→3)-α-Rha-(1→5)-α-Kdo(4P) trisaccharide motif that is conserved in *B. eggerthii* 1_2_48FAA (GCF_000185605.1) and *P. vulgatus* ATCC 8482 (GCF_000012825.1). The presence of a UDP-galactopyranose mutase (BLV03_RS02295), required for the biosynthesis of the UDP-galactofuranose donor substrate,[12] further supports the incorporation of β-Gal*f*. In contrast, the outer core biosynthetic region shows substantial divergence (Figure S10). The locus encodes glycosyltransferases from GT6, GT11, GT32, GT8, and GT90 families, suggesting a more specialized and potentially unique assembly pathway. The GT6 enzyme (BLV03_RS02320) (absent in most other Bacteroidales, based on the comparative GT heatmap, Figure S11) is consistent with the incorporation of α-Gal*p* identified experimentally.[13] BLV03_RS02325 was predicted to be a GT32-family enzyme likely accounting for the addition of the terminal β-Glc*p*NAc residue as in bacteria GT32 homologs have been described to transfer GlcNAc to tyrosine residues in *Yersinia* effector proteins[14] and to surface glycans in *Streptococcus*.[15] Notably, *B. eggerthii* 1_2_48FAA lacks the GT32 but present a GT14 (also predicted Core-

2/I-Branching enzyme, PF02485) that is known to have β-1,6-N-acetylglucosaminyltransferase activity[16] possibly explaining why both bacteria present a terminal GlcNAc. BLV03_RS02310 was predicted to be a GT90 by HMMER with dbCAN3 and as an EGF-domain serine glucosyl/xylosyltransferase [EC:2.4.1.376/2.4.2.63] based on KofamKOALA annotation (E value $6e^{-13}$). GT90 are known to act as protein *O*-β-glucosyltransferase/*O*-β-xylosyltransferase in human[17] and as a xylosyltransferase in *Cryptococcus deneoformans* where are involved in CPS and sphingolipids modification.[18,19] To the best of our knowledge, no bacterial GT90 has been functionally characterized to date (http://www.cazy.org). While the KofamKOALA annotation reflects the closest characterized homologs, the eukaryotic acceptor specificity, a serine residue within an EGF consensus motif, is not applicable to a bacterial LPS context,[20] and the true acceptor substrate of BLV03_RS02310 remains to be defined. Given the absence of canonical acceptor substrates in LPS biosynthesis, it is tempting to hypothesize that this enzyme may be involved in the installation of the unusual phosphodiester-linked hexose detected on Kdo, although its precise role remains to be established. Notably, BLV03_RS02315, which encodes a GT11-family enzyme typically associated with fucosylation[21,22] and present in related species such as *B. eggerthii* (GCF_000185605.1) and *P. vulgatus* (GCF_000012825.1), is present in the locus despite the absence of fucose in the determined structure. This suggests that fucosylation capacity may be inactive, differently regulated, or directed toward alternative substrates. Other more common GT families that were present in the region were a GT8 (BLV03_RS02305) and a third GT2 (BLV03_RS02300). The GT8 resulted to share 27.34 % sequence identity (BLASTp) whit *E. coli* RfaJ (UniProtKB/Swiss-Prot: P27129.2), a known glycosyltransferase, usually found in *E. coli* core OS gene cluster[23] and is also predicted to be an LPS 1-2-glucosyltransferase in *B. stercoris* DSM19555 genome (ID 46506.1562); KofamKOALA annotation predicted it to be an UDP-glucose:(galactosyl)LPS alpha-1,2-glucosyltransferase [EC:2.4.1.58] (E value $6.1e^{-53}$). Altogether consistent with the presence of a β-1,2 linked Glc residue according to NMR characterization.

Supporting note 4

Bioinformatic analysis of putative genes encoding for *B. stercoris* DSM 19555 CPS biosynthesis

Putative CPS loci were identified following the established *Bacteroides* CPS mining framework as implemented for *B. thetaiotaomicron*.[24] Candidate regions were defined by the presence of at least one *upxY* homolog[25] (the upstream activating element of CPS operons) flanked by glycosyltransferases with Pfam domains consistent with GT-A or GT-B folds. Among the six putative *upxY* homologs identified in the genome of *B. stercoris* DSM 19555, four were associated with at least one such glycosyltransferase, but only two (BLV03_RS04505 and BLV03_RS05350) were co-localized with a transcriptional repressor *upxZ* homolog within the same operon, corresponding to the canonical regulatory architecture of CPS loci in *Bacteroides* sp.[26] These two loci were therefore retained as primary CPS candidates, defining four multi-operon clusters (cps1-cps4; Figure 2 and Figure S12). Among these, we propose the cps1 locus (BLV03_RS05350–RS05435) as the biosynthetic cluster responsible for the characterized cationic CPS based on multiple convergent lines of evidence. The glycosyltransferase repertoire encoded within cps1 accounts for all sugar residues identified in the repeating unit in a near one-to-one correspondence, and a plausible biosynthetic sequence can be inferred. Assembly is predicted to initiate with RS05360 (GT4), which transfers GalNAc-1-phosphate onto the undecaprenyl carrier, forming the lipid-linked primer Und-PP-GalNAc. Although annotated as a GlcNAc-1-phosphate transferase by KofamKOALA [EC:2.7.8.33/2.7.8.35],[27] the absence of GlcNAc in the NMR-defined repeating unit suggests that the enzyme instead utilizes GalNAc, a reassignment consistent with the known substrate flexibility of GT4-family enzymes. In the subsequent step, BLV03_RS05435 is predicted (KofamKOALA, E-value 2.8e−45) to encode an N-acetylglucosaminyl-diphospho-decaprenol L-rhamnosyltransferase [EC:2.4.1.289], whose annotated acceptor substrate is a lipid-linked monosaccharide, consistent with the addition of α1-3 rhamnose at an early stage of assembly. BLV03_RS05410 is predicted (KofamKOALA, E-value 8.9e−12) to encode a β-1,4-N-acetylgalactosaminyltransferase and is assigned to the GT82 family by dbCAN3; this inverting GT-A fold family is restricted to prokaryotes and includes only one functionally characterized member, a β-1,4-N-acetylgalactosaminyltransferase from *Campylobacter jejuni*,[28] supporting its assignment to incorporation of another GalNAc residue. The remaining GT2-family

glycosyltransferases (RS05420 and RS05430; PF00535), given their broad donor specificity, are more consistent with the addition of galactose and remaining amino sugar residue. The locus also encodes the nucleotide-sugar biosynthetic machinery required for donor supply, including RS05390, a dTDP-glucose 4,6-dehydratase initiating dTDP-rhamnose biosynthesis, and RS05395, a predicted sugar nucleotidyltransferase likely involved in generating activated sugar donors. The regulatory architecture of cps1, with *upxY* upstream and *upxZ* downstream within the same operon, matches that of experimentally validated CPS loci in *Bacteroides*, while the presence of an integrase/recombinase gene immediately upstream is consistent with phase-variable regulation through invertible promoter elements, as described in *B. thetaiotaomicron* and *B. fragilis*.[29,30] In addition, cps1 encodes a PST-family polysaccharide transporter (BLV03_RS05405; KofamKOALA E-value 1.3e−93; PF13440, PF14667), supporting export and surface localization of the assembled polymer, as well as a putative lipopolysaccharide cholinephosphotransferase (LicD family protein, PF04991) [EC:2.7.8.-].

The enzymatic basis for the *N*-deacetylation of the GalN residue remains unresolved. Two CE4-family carbohydrate esterases are encoded outside cps1: a single-domain polysaccharide deacetylase (BLV03_RS03015) and a bifunctional protein (BLV03_RS12960) containing an N-terminal GH9 endoglucanase domain (PF00759) with a cellulase Ig-like module and a C-terminal NodB-like CE4 deacetylase domain (PF01522), as well as an N-terminal signal peptide consistent with extracytoplasmic localization. Structural modeling using AlphaFold3[31] and DALI[32] analysis confirmed the bifunctionality of BLV03_RS12960, identifying GH9 (PDB: 6fhj, 1ut9; Z-score 32.9) and CE4 (PDB: 3s6o, 2c1g; Z-score 16.5–20.8) enzymes among the top hits, together with carbohydrate deacetylases such as Agd (PDB: 6nwz; Z-score 11.4) and PgaB (PDB: 4f9j; Z-score 9.6).[33] Notably, PgaB selectively de-N-acetylates β-1,6-linked but not β-1,4-linked GlcNAc,[34] partially mirroring the structural context of the CPS described here, where the β-GalN residue is 4,6-substituted and the acetylated β-GalNAc units are substituted at O-3 or O-4. Similarly, Agd3 from *Aspergillus fumigatus* de-N-acetylates GalNAc residues in galactosaminogalactan, generating a cationic polymer essential for biofilm formation, and bacterial homologs of Agd3 have been identified in exopolysaccharide biosynthetic operons, suggesting that post-assembly de-N-acetylation may represent a conserved mechanism for generating cationic polysaccharides.[33] Whether BLV03_RS03015 or BLV03_RS12960 performs this function remains to be determined, and direct incorporation of GalN by a glycosyltransferase cannot be excluded.

The remaining CPS loci were excluded based on their predicted biosynthetic outputs. The cps2 locus (RS04445–RS04505) encodes enzymes directing synthesis toward UDP-glucuronic acid-containing polysaccharides, which are incompatible with the cationic CPS identified here. Similarly, cps3 (RS07630–RS07745) and cps4 (RS11430–RS11520) encode a UDP-N-acetyl-D-mannosaminuronic acid dehydrogenase, directing biosynthesis toward anionic ManNAcUA-containing polymers, and display a Wzy-dependent group 1 capsule architecture characterized by Wzc-type tyrosine kinases and CpsB/CapC phosphatases.[35-37] Importantly, none of these loci encodes a GT82-family glycosyltransferase or a glycosyltransferase repertoire matching the NMR-defined structure. A fifth locus containing an *upxY* homolog was also excluded, as its gene content closely matches the *nanLET/nanR* operon involved in sialic acid utilization in *B. fragilis*, including genes encoding sialidases (*nanL*), N-acyl-D-glucosamine-2-epimerase (*nanE*), a major facilitator superfamily transporter (*nanT*), and a ROK-family transcriptional regulator (*nanR*).[38]

Supporting note 5

Comparative CAZyme and GT family profiling across Bacteroidales

To contextualize the carbohydrate-active enzyme (CAZyme) repertoire of *B. stercoris* DSM 19555 within Bacteroidales, we computed and visualized CAZyme category distributions and glycosyltransferase (GT) family profiles across 38 reference genomes, including 26 *Bacteroides* spp., 4 *Phocaeicola* spp., 3 *Akkermansia* spp., *Odoribacter splanchnicus*, *Segatella copri*, and representative enteric outgroups (*E. coli* K-12 MG1655, *E. coli* O157:H7, and *Salmonella enterica* Typhimurium LT2). Genomes were annotated with dbCAN3 using HMMER (E-value < 1×10^{-15}), DIAMOND (E-value < 1×10^{-102}), and eCAMI substrate prediction, and only CAZymes supported by at least two methods were retained.

The CAZyme profile of *B. stercoris* DSM 19555 comprises 202 predicted enzymes, including 95 glycoside hydrolases (GH), 53 glycosyltransferases (GT), 23 polysaccharide lyases (PL), 15 carbohydrate esterases (CE), 0 auxiliary activity (AA) enzymes, and 16 carbohydrate-binding modules (CBM) (Figure S13). This repertoire places *B. stercoris* among species with intermediate glycan-processing capacity within the Bacteroidales panel. While GT and CE numbers fall within the typical range observed across *Bacteroides* spp. (GT: 31-94; CE: 7-44), the GH and CBM complements are markedly lower than those of highly saccharolytic species such as *B. cellulosilyticus* (423 GH, 77 CBM) and *B. ovatus* (351 GH, 62 CBM), which possess extensive polysaccharide utilization loci (PUL) repertoires adapted to complex dietary glycans.[39,40] This reduced GH/CBM content is consistent with a more restricted substrate utilization range and a comparatively limited PUL repertoire, in line with previous observations that *B. stercoris* lacks specific loci required for the metabolism of selected glycans.[41]

Comparative analysis of GT family distributions (Figure S11) highlights a marked functional divergence between gut commensals and enteric pathogens, alongside a selective convergence between commensal bacteria and host glycosylation pathways.[42,43] Human cells express 155 GT genes across 44 families, several of which (including GT6, GT8, GT10, GT11, GT14, GT23, and GT32) are also detected in commensal genomes. Notably, *B. stercoris* encodes GT6 (1 copy), GT8 (2 copies), GT11 (1 copy), and GT32 (2 copies), while GT14 is present in a subset of related species (e.g. *B. thetaiotaomicron*, *P. vulgatus*, *P. dorei*, and selected *Akkermansia* spp.). GT32 is broadly distributed (24/35 commensal species) and includes functionally diverse enzymes, ranging from β-1,4-GlcNAc transferases in bacteria to enzymes involved in mucin-type glycan and glycosphingolipid biosynthesis in mammals. [44]

A notable feature of the commensal GT repertoire is the widespread presence of fucosyltransferase-associated families (GT10, GT11, GT23), detected in 27 out of 35 commensal genomes. For example, *P. dorei* encodes GT10 (1 copy), GT11 (4 copies), and GT23 (2 copies), indicating a substantial capacity for the biosynthesis of fucosylated glycans, while *A. muciniphila* encodes multiple GT10 and GT11 homologs. Given that GT10 and GT11 are involved in the synthesis of Lewis-type epitopes[21] and GT23 contributes to Fucα1-6GlcNAc linkages[45], their prevalence supports a broader trend toward host glycan mimicry among gut commensals. In contrast, facultative enteric bacteria such as *E. coli* and *S. enterica* lack these GT families but are enriched in GT9 enzymes (3-4 copies), which mediate ADP-heptose-dependent inner core LPS biosynthesis. Consistently, heptose residues are absent or under-represented in Bacteroidales LPS structures, reflecting distinct evolutionary and ecological strategies between commensal and pathogenic bacteria. Together, these data position *B. stercoris* within a commensal-specific glycosylation landscape characterized by a GT repertoire partially overlapping with host glycosylation pathways, supporting its potential to produce structurally diverse and host-relevant glycoconjugates.

Supporting note 6

Methods

Bacterial Growth

Bacteroides stercoris DSM 19555 was obtained from the DSMZ, German Collection of Microorganisms and Cell Cultures GmbH, Braunschweig, Germany. It was grown in yeast casitone fatty acid medium with glucose, starch, cellobiose (YCFAGSC)[46] with the following modifications: the short-chain fatty acid mix was omitted, yeast extract was increased to 0.5% final concentration and the final hemin concentration was reduced to 1 μg/mL. To achieve anaerobic conditions, the medium was boiled before and after adding cysteine (0.1% final concentration) as a reducing agent and flushed with carbon dioxide. The pH was set to 7.5 when the medium reached approximately 65 °C to achieve a final medium pH of approximately 6.5 after autoclaving. Cultures were grown static anaerobically (under carbon dioxide) at 37 °C overnight. Pre-cultures were grown in Hungate tubes (10 mL medium), main cultures were grown after inoculation with 1% (v/v) preculture in 100 ml Wheaton bottles. Cells were harvested by centrifugation in sterile 250 ml Sorvall Lynx bottles (Thermo Fisher Scientific, Paisley, UK) for 15 min at 30239 × *g* (14000 rpm), 4 °C, in a Sorvall Lynx centrifuge (Thermo Fisher Scientific, Paisley, UK). The supernatant was discarded and the pellet was resuspended in 40

mL deionized sterile water, transferred to 50 mL Falcon tubes and centrifuged for 20 min at 6000 × *g*, 4 °C, in Jouan centrifuge MR 18 22 (DJB Labcare Ltd, Newport Pagnell, UK). The supernatant was discarded and the pellet was freeze-dried for 3-4 days in an Edwards Modulyo Freeze Dryer (Edwards Ltd, Burgess Hill, UK).

R-LPS isolation and purification

Freeze-dried *B. stercoris* DSM 19555 cells were subjected to extraction using the phenol/chloroform/light petroleum protocol.[47] The crude extract was subsequently purified by repeated ultracentrifugation cycles (100,000 × *g*, 24 h, 4 °C), followed by size-exclusion chromatography on a Sephacryl S-300 High Resolution column (GE Healthcare, Little Chalfont, UK), eluted with 50 mM ammonium bicarbonate. Fractions containing R-LPS were pooled, lyophilized and then treated with sequential organic solvent washes, first using chloroform/methanol (1:2, v/v) and then chloroform/methanol/water (3:2:0.25, v/v), to remove contaminating phospholipids. After complete solvent removal, the R-LPS fraction was subjected to an additional lipoprotein depletion step following the procedure described by Hirschfeld *et al.*[48] Purity of the R-LPS preparation was assessed by SDS-PAGE followed by silver staining, and potential protein or lipoprotein contamination was evaluated by Coomassie Brilliant Blue staining on parallel gels. In SDS-PAGE analysis, the purified R-LPS was prepared at the concentration of 1 mg/mL and boiled (10 min). Then, 8 µL/well were loaded on a 13.5% SDS gel with a 5% stacking gel and separated using a mini-PROTEAN electrophoresis instrument (Bio-Rad Laboratories). Residual protein content was quantified using the Pierce Micro BCA Protein Assay (Thermo Scientific, #23235), performed at a concentration 10,000-fold higher than that used in cell-based assays. To minimize lipid interference, the assay was performed in the presence of 2% SDS. Absorbance was measured at 562 nm using a TECAN Infinite M Plex spectrophotometer (Tecan, Grödig, Austria).

CPS isolation and purification

CPS was extracted using a hot phenol/water protocol adapted from previously described procedures.[49] Briefly, bacterial pellet, previously subjected to R-LPS extraction, was resuspended in water and extracted with preheated 75% phenol at 68 °C under vigorous mixing. Following incubation, phase separation was achieved by centrifugation, and the aqueous phase containing CPS together with residual R-LPS was collected. Residual phenol was removed by extensive dialysis (cutoff 6-8 kDa) against distilled water. The dialyzed extract was then treated sequentially with DNase (Roth) and RNase (Roth), followed by proteinase K (Roth) digestion, to remove nucleic acids and protein contaminants. CPS was subsequently precipitated by addition of cold ethanol, and the resulting pellet was collected by centrifugation, resuspended in water, and lyophilized. To eliminate residual R-LPS, the sample was subjected to repeated ultracentrifugation steps (100,000 × *g*, 16 h, 4 °C). After each cycle, both supernatant and pellet were analyzed by SDS-PAGE followed by silver and Coomassie Blue staining as well as by 1D NMR. Supernatants enriched in CPS were pooled and subjected to final purification by size-exclusion chromatography on a Sephacryl S-400 High Resolution column (GE Healthcare, Little Chalfont, UK) in the presence of deoxycholate to achieve complete separation from R-LPS. CPS-containing fractions were collected, extensively dialyzed to remove detergent, and lyophilized. The purity of the CPS preparation was assessed by SDS-PAGE and NMR spectroscopy, confirming the absence of R-LPS and (lipo)protein contaminants.

Chemical characterization

Monosaccharide composition was determined by GC-MS analysis of acetylated *O*-methyl glycoside (AMG) derivatives obtained after methanolysis of an aliquot of the R-LPS and CPS sample with 1.25 M HCl in methanol (85 °C, 16 h), followed by acetylation with acetic anhydride in pyridine (85 °C, 30 min).[50] The absolute configuration of the monosaccharides was established by preparing the corresponding *O*-octyl glycoside (OGA) derivatives and analyzing them as previously described.[51] Glycosidic linkage analysis was performed by conversion of monosaccharides into partially methylated alditol acetates (PMAA), followed by GC-MS analysis. Briefly, dried samples were suspended in DMSO containing powdered NaOH and subjected to alternating stirring and sonication for 2 h at room temperature prior to methylation with iodomethane (16 h, room temperature). Samples were then treated with sodium tetradeuteroborate ($NaBD_4$). Subsequent steps included hydrolysis with trifluoroacetic acid (2 M, 100 °C, 4 h), carbonyl reduction again with $NaBD_4$, and

final acetylation with acetic anhydride in pyridine (85 °C, 30 min). Fatty acid composition was determined by subjecting R-LPS to acid hydrolysis (4 M HCl, 100 °C, 4 h) followed by alkaline treatment (5 M NaOH, 100 °C, 30 min). After adjusting the pH to approximately 3, fatty acids were extracted with chloroform, methylated using diazomethane, and analyzed by GC-MS. Ester-bound fatty acids were selectively released by treatment with 0.5 M NaOH in methanol/water (1:1, v/v) at 85 °C for 2 h, followed by acidification, extraction, methylation, and GC-MS analysis. In parallel, fatty acids were also analyzed as methyl esters generated during AMG preparation and compared with those obtained by the procedures described above. In addition, an independent methanolysis (1.25 M HCl in methanol, 85 °C, 16 h) was performed to generate fatty acid methyl esters, which were extracted in hexane and analyzed by GC-MS. The absolute configuration of hydroxylated fatty acids was determined by releasing 3-hydroxy fatty acids through alkaline hydrolysis (4 M NaOH, 100 °C, 5 h), followed by conversion into their 3-methoxy acid L-phenylethylamide derivatives and GC-MS analysis.[52] Assignment was achieved by comparison of retention times with those of authentic standards, confirming the *R* configuration for all 3-hydroxy fatty acids. All derivatives were analyzed using an Agilent 7820A gas chromatograph coupled to a 5977B mass selective detector, equipped with an HP-5 capillary column (30 m × 0.25 mm i.d.; helium carrier gas, 1 mL/min). The temperature program for AMG and OGA derivatives was: 140 °C (3 min), then 140–240 °C at 3 °C/min. PMAA were analyzed using the following program: 90 °C (1 min), 90–140 °C at 25 °C/min, 140–200 °C at 5 °C/min, 200–280 °C at 10 °C/min, and 280 °C for 10 min. Fatty acid methyl esters were analyzed using: 150 °C (5 min), 150–280 °C at 3 °C/min, and 280 °C for 5 min.

Isolation of the core OS and lipid A from R-LPS

An aliquot of purified R-LPS was subjected to *O*-deacylation by treatment with anhydrous hydrazine (2 mL) under stirring at 37 °C for 90 min. After cooling, the reaction mixture was added dropwise to ice-cold acetone (~10 mL), and the resulting precipitate was collected by centrifugation (4000 × *g*, 30 min), extensively washed with cold acetone, dried, resuspended in water, and lyophilized. The *O*-deacylated product was subsequently *N*-deacylated by treatment with 4 M KOH at 120 °C for 16 h. Desalting was performed by gel filtration on a Sephadex G-10 column (50 × 1.5 cm, Pharmacia). Final purification of the fully deacylated product was achieved by size-exclusion chromatography on a Bio-Gel P4 (Bio-Rad Laboratories) using water as eluent. The purified material was then analyzed by 1D and 2D NMR spectroscopy. In parallel, a separate aliquot of R-LPS was subjected to mild acid hydrolysis in acetate buffer (pH 4.4) at 100 °C for 2 h under continuous stirring. After hydrolysis, a mixture of methanol and chloroform was added to reach a final $CH_3OH/CHCl_3$/hydrolysate ratio of 2:2:1.8 (v/v/v). The suspension was vigorously mixed and centrifuged (8800 × *g*, 30 min, 4 °C). The water phase, containing the carbohydrate fraction, was collected, and lyophilized, whereas the lower chloroform phase, containing the lipid A fraction, was recovered and further washed using the aqueous phase of a freshly prepared Bligh and Dyer mixture ($CHCl_3/CH_3OH/H_2O$, 2:2:1.8, v/v/v). Organic phases were combined, dried, and analyzed by MALDI-TOF MS for lipid A characterization. The lyophilized carbohydrate-containing fraction was further purified by size-exclusion chromatography on a Bio-Gel P4 column (Bio-Rad Laboratories) and subsequently analyzed by NMR spectroscopy.

NMR spectroscopy

1D and 2D NMR spectra were recorded at 298 K in D_2O (pD 7) on a Bruker AVANCE NEO 600 MHz spectrometer equipped with a cryoprobe. Chemical shifts were referenced to internal acetone (δ_H 2.225 ppm; δ_C 31.45 ppm). ^{31}P NMR experiments were performed on a Bruker DRX-400 spectrometer using 85% aqueous phosphoric acid as external reference (δ 0.00 ppm). Double-quantum-filtered phase sensitive correlation spectroscopy (DQF-COSY) experiments were recorded by using data sets of 4096 × 512 points. Total correlation spectroscopy (TOCSY) experiments were carried out with spinlock times of 100 ms using data sets (t1 × t2) of 4096 × 256 points. Rotating frame Overhauser enhancement spectroscopy (ROESY) was performed using data sets (t1 × t2) of 4096 × 256 points and mixing times between 100 and 300 ms, acquiring 16 scans. The data matrix in all the homonuclear experiments was zero-filled in both dimensions to give a matrix of 4K × 2K points and was resolution-enhanced in both dimensions by a cosine-bell function before Fourier transformation. Coupling constants were attained from 2D phase-sensitive DQF-COSY. Heteronuclear single quantum coherence (^{1}H,^{13}C HSQC) and heteronuclear multiple bond correlation (^{1}H,^{13}C HMBC) experiments

were carried out in ^{1}H-detection mode by single-quantum coherence with proton decoupling in the ^{13}C domain using data sets of 2048 × 256 points. ^{1}H,^{13}C HSQC was performed using sensitivity improvement and in the phase-sensitive mode using Echo/Antiecho gradient selection, with multiplicity editing during selection step. ^{1}H,^{13}C HMBC was optimized on long range coupling constants, with low-pass J-filter to suppress one-bond correlations, using gradient pulses for selection. A 60 ms delay was adopted for the evolution of long-range correlations. ^{1}H,^{13}C HMBC was optimized for a 6 Hz coupling constant, and ^{1}H,^{31}P HSQC was optimized for an 8 Hz coupling constant. The data matrix in all the heteronuclear experiments was extended to 2048 × 1024 points by using forward linear prediction extrapolation.[53]

ESI MS and MS/MS analysis

MS and MS/MS analyses were carried out on a Waters Synapt XS mass spectrometer equipped with an 8 kDa quadrupole. R-LPS was dissolved in a 50:50 (v/v) 2-propanol/water mixture and directly infused into an electrospray ionization (ESI) source using a syringe pump at a flow rate of 10 μL min^{-1} at an estimated concentration of 20 μg m L^{-1}. All the spectra were acquired in negative polarity and Resolution mode, within a scan range of *m/z* 50–1800. For MS analysis, the following parameters were applied: capillary voltage -2.3 kV, source temperature 120 °C, sampling cone voltage 40 V, source offset 10 V, source gas (N2) flow 0.0 mL min^{-1}, desolvation temperature 300 °C, cone gas flow 20 L h^{-1}, desolvation gas flow 300 L h^{-1}, scan time 0.5 s, and nebulizer gas pressure 2.5 bar. MS/MS experiments on the precursor ion at *m/z* 1554.305 was conducted with a scan range of *m/z* 50–3500 in the trap cell using ultra-pure argon (SOL SpA) as collision gas, with a fixed collision energy of 30 eV, and a scan time of 0.5 s. Technical parameters for MS/MS analysis were: capillary voltage -2.2 kV, source temperature 120 °C, sampling cone voltage 40.0 V, source offset 10 V, source gas (N2) flow 0.0 mL min^{-1}, desolvation temperature 300 °C, cone gas flow 20 L h^{-1}, desolvation gas flow 350 L h^{-1}, and nebulizer gas pressure 2.5 bar. MS/MS analysis of the ion at *m/z* 1419.349 was conducted with a scan range of *m/z* 50–3500 using ultra-pure argon (SOL SpA) as collision gas, with a fixed collision energy of 68 eV, and a scan time of 0.5 s and the following technical parameters: capillary voltage -2.3 kV, source temperature 120 °C, sampling cone voltage 140.0 V, source offset 10 V, source gas (N2) flow 0.0 mL min^{-1}, desolvation temperature 300 °C, cone gas flow 20 L h^{-1}, desolvation gas flow 300 L h^{-1}, and nebulizer gas pressure 2.5 bar. Nitrogen was generated by a PLINIUS N45-1 system (CLAIND) and employed as both desolvation and cone gas. Instrument calibration over the 50–3500 Da range was performed using a 2 μg/μL NaI solution in 1:1 (v/v) 2-propanol/water. All spectra were acquired and subsequently processed using MassLynx™ software (V4.2).

MALDI-TOF MS analysis

MALDI-TOF MS spectrum was acquired using reflectron mode and negative ion polarity on an ABSCIEX TOF/TOF™ 5800 mass spectrometer (Applied Biosystems, Foster City, CA, USA). The instrument was equipped with an Nd:YAG laser (λ = 349 nm), a 3 ns pulse width, and a repetition rate of up to 1000 Hz, along with delayed extraction technology. Lipid A from *B. stercoris* was dissolved in a chloroform/methanol 50:50 (v/v) mixture, while the matrix solution was 2,4,6-trihydroxyacetophenone dissolved in methanol/0.1% trifluoroacetic acid/acetonitrile 7:2:1 (v/v/v) at a concentration of 75 mg mL^{-1}.[54] For analysis of lipid A directly from bacterial pellet, 2,5-dihydroxybenzoic acid (DHB) at 10 mg/mL in chloroform/methanol (9:1, v/v) was used as the matrix.[55] 0.5 μL of the sample and 0.5 μL of the matrix solution were applied to a stainless-steel target plate and allowed to air dry at room temperature. Each MS spectrum was averaged from 2000 laser shots, while MS/MS spectra were obtained by accumulating 5000-7000 shots. Acquisition was performed ensuring random yet uniform sampling across the sample spot.

Bioinformatics analysis

To characterize the R-LPS and CPS biosynthetic gene clusters of *B. stercoris* DSM 19555, we retrieved the annotated genome from NCBI RefSeq (accession GCF_900106605.1) and predicted operon boundaries, COG assignments, and protein sequences using the Operon-mapper web server.[9] Functional annotation was performed by submitting all predicted protein sequences to KofamKOALA[56] and to InterProScan[6] for Pfam

domain identification. Glycosyltransferase CAZy family assignments were determined using dbCAN3 (v5.2.8),[7] employing three complementary tools: HMMER (E-value < 1×10^{-15}), DIAMOND (E-value < 1×10^{-102}), and eCAMI for substrate prediction. The putative R-LPS biosynthetic locus was identified by searching for homologs of key conserved genes including *msbA* and GT9-family glycosyltransferases, following established knowledge on LPS loci genetic content.[57, 58] Candidate CPS loci were identified by locating operons encoding the transcriptional regulators *UpxY* (COG0250; PF02357) and *UpxZ* (PF06603), followed by screening the surrounding genomic regions for genes belonging to COG class M (Cell wall/membrane/envelope biogenesis), with particular emphasis on glycosyltransferases carrying Pfam domains PF00534, PF00535, PF01755, PF02397, PF02485, PF08759, PF13439, PF13477, PF13579, PF13692, and PF14305, as described for *B. thetaiotaomicron* VPI-5482, *B. fragilis* NCTC9343, and *P. vulgatus* ATCC 8482.[24] Genomic regions and operons of interest were visualized using a custom script based on the DNA Features Viewer library.[59] The putative LPS biosynthetic loci of *B. eggerthii* 1_2_48FAA (GCF_000185605.1) and *P. vulgatus* ATCC 8482 (GCF_000012825.1), two species for which we previously elucidated the LPS structure [4, 9] were identified using the same approach and aligned to the *B. stercoris* R-LPS locus by BLASTn.[60] Homologous regions were visualized with Easyfig 2.2.5.[10] To contextualize the CAZyme repertoire of *B. stercoris* DSM 19555 within Bacteroidales, a comparative analysis was conducted across 38 reference genomes obtained from NCBI, comprising 26 *Bacteroides* spp., 4 *Phocaeicola* spp., 3 *Akkermansia* spp., *Odoribacter splanchnicus*, *Segatella copri*, and three enteric outgroup strains (*Escherichia coli* K-12 MG1655, *E. coli* O157:H7, and *Salmonella enterica* serovar Typhimurium LT2). All genomes were annotated with dbCAN3 (v5.2.8) using the same filtering criteria described above. CAZyme category distributions and glycosyltransferase (GT) family counts were computed per genome. To reduce the influence of highly expanded families, GT counts were log-transformed as $\log(1 + x)$ prior to hierarchical clustering. Genomes were clustered based on their GT family profiles using Euclidean distance with the UPGMA (average) linkage method, as implemented in the SciPy library.[61] GT families were ordered numerically along the column axis without clustering. Heatmaps and row dendrograms were generated using the seaborn clustermap function.[62]

Reagents and cell lines

HEK-Blue™ and THP-1 Blue™ cells lines were provided by InvivoGen (Toulouse, France). Caco-2 cells (#300137) were provided by Cytion biosciences (Heidelberg, Germany). HEK-Blue™ cells were cultured in Dulbecco's Modified Eagle's Medium (DMEM) supplemented with 10% heat inactivated fetal bovine serum (FBS), 1% penicillin/streptomycin, 2 mM l-glutamine and 100 µg mL^{-1} Normocin. THP-1 Blue™ cells were cultured in RPMI medium supplemented with 10% heat-inactivated FBS, 2 mM l glutamine, 1% Pen/Strep and HEPES 1 mM. To ensure plasmid selection, HEK Blue™ selection and blasticidin were added, according to the manufacturer instruction, to the HEK-Blue™ cell and THP-1 culture medium respectively. Caco-2 cells were cultured in DMEM supplemented with 10% heat inactivated fetal bovine serum (FBS), 1% penicillin/streptomycin, 2 mM l-glutamine and MEM Non-Essential Amino Acids 1%. All cell lines were grown at 37 °C in a humidified atmosphere containing 5% CO2. Cell culture products were purchased from Thermo Fisher Scientific (Gibco). In addition, normocin, blasticidin, HEK Blue selection, *E. coli* O111:B4 LPS, *Rhodobacter sphaeroides* (RS) LPS (#tlrl-prslps) and QUANTI-Blue™ - SEAP Detection Reagent were obtained from InvivoGen (Toulouse, France). Phorbol 12-myristate 13-acetate (PMA) was obtained from Thermo Fisher Scientific (Kandel, Germany).

LPS stimulation of HEK-Blue™ cell reporter lines and Competition assays

HEK-Blue™ hTLR4, HEK-Blue™ MD2-CD14, HEK-Blue™ hTLR2, HEK-Blue™ hTLR2/1 and HEK-Blue™ hTLR2/6 cells were seeded in a 96-well plate (3×10^4 cells per well). After allowing overnight attachment, HEK-Blue™ cells were stimulated overnight with various concentrations of *B. stercoris* R-LPS or CPS. *E. coli* LPS served as positive control in HEK-Blue™ hTLR4 based assays. PAM3CSK4 (500 ng mL^{-1}) for TLR2 and TLR2/1 activation, PAM2CSK4 (500 ng mL^{-1}) for TLR2/6 activation were used as positive controls. TNFα (1 ng mL^{-1}) was used as positive control for HEK-Blue™ MD2-CD14 cell line. HEK-Blue™ cell supernatants were collected to assess the secreted embryonic alkaline phosphatase (SEAP) levels, using QUANTI-Blue™ - SEAP Detection Reagent, as an indicator of NF-κB activation following LPS

stimulation, according to the manufacturer's protocol. The absorbance at 620 nm was measured using a TECAN Infinite M Plex spectrophotometer (Tecan, Grödig, Austria). For competition assays, HEK-Blue™ hTLR4 cells were primed with various concentrations of *Rhodobacter sphaeroides* LPS (LPS RS) or *B. stercoris* R-LPS or CPS for 4 h and then stimulated with 10 ng mL^{-1} of *E. coli* LPS for additional 16 h. Cell supernatants were collected to assess the SEAP levels using QUANTI-Blue™ - SEAP Detection Reagent. Results were represented has fold of the control group stimulated with 10 ng mL^{-1} or 100 ng mL^{-1} of *E. coli* LPS for 16 h.

NF-κB activation by *B. stercoris* glycoconjugates under TLR4 inhibitory conditions

THP-1 were primed for 2 h with increasing concentration of LPS RS (1-1000 ng mL^{-1}), afterwards *B. stercoris* R-LPS or *E. coli* LPS were added with a final concentration of 10 ng mL^{-1}. *B. stercoris* CPS was added with a final concentration of 1000 ng mL^{-1}. The interference mediated by LPS RS was compared to controls stimulated only with *B. stercoris* R-LPS, *E. coli* LPS (10 ng mL^{-1}) or *B. stercoris* CPS (1000 ng mL^{-1}). NF-κB activity was quantified as previously described.

Cell viability (MTT) assay

Cell viability of THP-1 derived macrophages was assessed using a 3-(4,5-dimethylthiazol-2-yl)-2,5-diphenyltetrazolium bromide (MTT) assay. Cells were treated with medium alone or containing various concentrations of *E. coli* LPS or *B. stercoris* R-LPS or CPS (1-1000 ng mL^{-1}) for 24 h or 48 h in a 96-well plate. At the end of treatment supernatants were collected and, 100 µL of medium complemented with MTT (0.5 mg mL^{-1}) was added to each well. After 3 hours of incubation at 37 °C, the medium was removed, and the formazan crystals were dissolved with 100 µL of dimethyl sulfoxide (DMSO). Absorbance was measured on a TECAN Infinite M Plex spectrophotometer (Tecan, Grödig, Austria). Cell viability data were normalized towards unstimulated control cells.

Differentiation of THP-1 Blue™ cells

THP-1 Blue™ cells were differentiated in M0, M(INFγ+LPS) and M(IL-4) according to Baxter *et al.*[63] Briefly M0 was obtained by stimulating cells for 24 h with 8 nM PMA. Then, PMA complemented medium was discarded and freshly prepared complete culture medium was added. Cells were rested for 72 h. Differentiated THP-1 were used directly for experiment or further differentiated in M(IL-4) and M(INFγ+LPS). Proper differentiation was confirmed by adhesion of the cells and by flow cytometry experiment.

Flow Cytometry experiments on differentiated THP-1 macrophages

Fluorochrome-conjugated anti-human monoclonal antibodies were purchased from Miltenyi Biotec (Bergisch Gladbach, Germany): CD11c-Vioblue (clone REA618, Cat. 130-120-032), CD14-PE-Vio770 (clone REA599, Cat. 130-110-521), CD80-VioBright B515 (clone REA661, Cat. 130-130-555), HLA-DR-APC-Vio770 (clone REA805, Cat. 130-111-792), CD200R-APC (clone REA725, Cat. 130-111-291), and CD206-PE (clone REA1207, Cat. 130-123-159). 7-AAD viability dye (Cat. 130-111-568) was used immediately prior to acquisition. All reagents were used as provided by the manufacturer. Cells were washed with PBS, resuspended in 100 µL of antibody master mix, and incubated for 15 min at room temperature protected from light. After staining, samples were washed with 1 mL of PBS and centrifuged for 5 min at 300 × *g*. Pellets were resuspended in 100 µL of acquisition buffer. Immediately before analysis, 7-AAD was added according to manufacturer instruction. Samples were acquired within 30 min of staining. Data were collected on a flow cytometer equipped with 405, 488, and 633 nm lasers (MACSQuant Analyzer 10, Miltenyi Biotec). Flow cytometry data were analyzed using MACSQuantify software (version 2.13.3; Miltenyi Biotec). A standardized gating strategy was applied to all samples (Figure S17). Debris was excluded based on forward and side scatter (FSC-A vs SSC-A). Doublets were excluded using FSC-H versus FSC-A. Viable cells were identified by exclusion of 7-AAD-positive events. Marker-positive populations were defined using unstained controls, while voltages and compensation matrices were optimized using fluorescence minus one (FMO) controls prior to acquisition.

Differentiation of Caco-2 cells and coculture experiments

Caco-2 cells were seeded 1.8E+5 cells cm^{-2} in 24-well semi-permeable inserts (Falcon® Permeable Support for 24-well Plate with 0.4 µm Transparent PET Membrane, #353095). Caco-2 cell monolayers were cultured for 14 days, until a functional cell monolayer with a Transepithelial electrical resistance (TEER) of more than 300 Ω x cm^2 was obtained.[64] Medium was changed every 2-3 days and prior to co-culture, medium in the basolateral compartment was gradually changed to RPMI. TEER was measured using an EVOM™ Manual Meter (World Precision Instrument, EVMMT0302), the TEER of an empty insert was subtracted from all readings. Further confirmation of Caco-2 cells differentiation was obtained by observing occludin and claudin-1 expression at the protein level using western blot technique. The co-culture model was assembled by placing M(INFγ+LPS) macrophages (obtained as previously described) in the basolateral compartment. A resting of 24 h was implemented to allow the system to stabilize. *E. coli* LPS, B. *stercoris* LPS or CPS were then added apically, and the systems were incubated for 18 h.

Cytokines quantification upon *E. coli* LPS or *B. stercoris* R-LPS and CPS stimulation

Cytokine levels for CXCL8, IL-10, IL6 and TNFα were quantified from the supernatants using a DuoSet ELISA kit (R&D system, Bio-Techne, Minneapolis, USA). Unstimulated cells were considered as a control to quantify basal cytokine levels. Absorbance values were read at 450 nm with background subtraction at 570 nm using a TECAN Infinite M Plex spectrophotometer (Tecan, Grödig, Austria). Cytokine concentrations (pg mL^{-1}) were determined from a standard curve prepared from cytokine standards according to the manufacturer's protocol.

Western Blot

THP-1 cells from monoculture experiments, THP-1 M(INFγ+LPS) and differentiated Caco-2 cells from the co-culture system were lysed in RIPA buffer (Thermo Fisher Scientific) supplemented with phosphatase and protease inhibitor cocktails (Thermo Fisher Scientific). Total protein content was quantified using a BCA Protein Assay Kit (Thermo Fisher Scientific). For western blot analysis, 20 µg of protein per sample was resolved on 4-20% Mini-PROTEAN® TGX Stain-Free™ Protein Gels (Bio-Rad) and transferred to PVDF membranes using the Trans-Blot® Turbo™. The following primary antibodies were used: phospho-NF-κB p65 (Ser536) (1: 1000; #3033, Cell Signaling Technology, CST), total NF-κB p65 (1: 1000; #8242, CST), GAPDH (1 : 3000; #97166, CST), phospho-STAT3 (phospho S727) (1:1000; ab32143, abcam), STAT3 (1:1000; ab68153, abcam), Claudin-1(1:1000; #13995 CST), Occludin (1:1000; #31131 CST), phospho-p38 MAPK (Thr180/Tyr182) (1:1000; #9211 CST), p38 MAPK(1:1000; #9212 CST), α-Tubulin (1:3000; #3873 CST. HRP-conjugated secondary antibodies-anti-rabbit IgG (#7074, CST) and anti-mouse IgG (#7076, CST) were used at a 1:3000 dilution. Immunoreactive bands were visualized using chemiluminescence and imaged with an Azure 600® Western Blot Imaging Systems (Azure Biosystems, Inc.)

FITC dextran permeability

FITC-dextran 4 kDa was added to the apical chamber of the Transwell™ inserts at a concentration of 1 mg mL^{-1} dissolved in complete cell culture medium. After 2 h of incubation, supernatants from the basolateral chambers were collected and analyzed for FITC by fluorometry at an excitation of 485 nm and an emission of 535 nm using the plate reader TECAN Infinite M Plex spectrophotometer (Tecan, Grödig, Austria).

Statistical Analysis

Statistical analyses were performed using GraphPad Prism (version 10.0, GraphPad Software, San Diego, CA, USA). Data are presented as mean ± standard deviation (SD) of at least three independent biological replicates, unless otherwise stated. Differences among multiple groups were assessed by one-way ANOVA followed by Dunnett’s multiple comparison test, using the unstimulated control as the reference group. For flow cytometry data, one-way ANOVA with Dunnett’s post hoc test was applied, comparing each treatment group against the resting M0 macrophage control. Statistical significance was set at $p < 0.05$.

Supporting References

1. Birnbaum, G. I. Conformations of ammonium 3-deoxy-D-manno-2-octulosonate (KDO) and methyl α- and β-ketopyranosides of KDO: X-ray structure and ^{1}H NMR analyses. *J. Carbohydr. Chem.* **6**, 17–39 (1987).
2. Berezow, A. B. et al. The structurally similar, penta-acylated lipopolysaccharides of Porphyromonas gingivalis and Bacteroides elicit strikingly different innate immune responses. *Microbial Pathogenesis*, **47**, 68–77 (2009).
3. Di Lorenzo, F., De Castro, C., Silipo, A., & Molinaro, A.. Lipopolysaccharide structures of Gram-negative populations in the gut microbiota and effects on host interactions. *FEMS Microbiol. Rev.* **43**, 257–272 (2019).
4. Pither, M. D. et al. *Bacteroides thetaiotaomicron* rough-type lipopolysaccharide: The chemical structure and the immunological activity. *Carbohydr. Polym.* **297**, 120021 (2022).
5. Wang Y, Kalka-Moll WM, Roehrl MH & Kasper DL. Structural basis of the abscess-modulating polysaccharide A2 from Bacteroides fragilis. *Proc Natl Acad Sci. U S A* **97***,* 13478-83(2000)
6. Paysan-Lafosse, T. et al. The Pfam protein families database:embracing AI/ML. *Nucleic Acids Res.* ***53***, D3689–D378 (2025).
7. Zheng, J. et al. dbCAN3: automated carbohydrate-active enzyme and substrate annotation. *Nucleic Acids Res.* **51**, W115–W121 (2023).
8. Lombard, V., Golaconda Ramulu, H., Drula, E., Coutinho, P. M. & Henrissat, B. The carbohydrate-active enzymes database (CAZy) in 2013. *Nucleic Acids Res.* **42**, D490–D495 (2014).
9. Taboada, B. et al. Operon-mapper: a web server for precise operon identification in bacterial and archaeal genomes. *Bioinformatics* **34** 4118-4120 (2018)
10. Sullivan, M.J., Petty, N.K. & Beatson, S.A. Easyfig: a genome comparison visualizer. *Bioinformatics* **27**, 1009–1010 (2011).
11. De Simone Carone L. et al. Decoding a Gut Commensal Signal: Structural and Immunological Profiling of Segatella Copri Lipopolysaccharide. *Angew. Chem. Int. Ed.* **64**, e202512947 (2025).
12. Seničar, M. et al. Galactofuranose-Related Enzymes: Challenges and Hopes. *Int. J. Mol. Sci.* **21**, 3465 (2020).
13. Brew, K., Tumbale, P. & Acharya, K.R. Family 6 glycosyltransferases in vertebrates and bacteria: inactivation and horizontal gene transfer may enhance mutualism between vertebrates and bacteria. *J. Biol. Chem*. **285**, 37121–37127 (2010).
14. Schneider, S. et al. Tyrosine-modifying glycosylation by Yersinia effectors. *J. Biol. Chem*. **300**, 107331 (2024).
15. Oliver, M.B. et al. Streptococcus pneumoniae serotype 11D has a bispecific glycosyltransferase and expresses two different capsular polysaccharide repeating units. *J. Biol. Chem*. **288**, 21945–21954 (2013).
16. Bierhuizen, M F et al. Genomic organization of core 2 and I branching beta-1,6-N-acetylglucosaminyltransferases. Implication for evolution of the beta-1,6-N-acetylglucosaminyltransferase gene family. *Glycobiology* **5**, 417–425 (1995)
17. Li, Z. et al. Structural basis of Notch O-glucosylation and O–xylosylation by mammalian protein–O-glucosyltransferase 1 (POGLUT1). *Nat. Commun.* **8**, 185 (2017).
18. Klutts, J. S. & Doering, T. L. Cryptococcal xylosyltransferase 1 (Cxt1p) from Cryptococcus neoformans plays a direct role in the synthesis of capsule polysaccharides. *J. Biol. Chem*. **283**, 14327–14334 (2008).
19. Castle, S. A. et al. Beta1,2-xylosyltransferase Cxt1p is solely responsible for xylose incorporation into Cryptococcus neoformans glycosphingolipids. *Eukaryot. Cell* **7,** 1611-1615 (2008).
20. Xia, Y. et al. Structural basis of EGF-repeat O-glucosylation by the pr8otein O glucosyltransferase POGLUT2. *J. Biol. Chem*. **302**, 111361 2026).
21. Ghirardello, M.et al. Structure, function, and implications of fucosyltransferases in health and disease. *Nat. Commun*. **16**, 11279 (2025).

22. Garcia-Vello, P.et al. The lipooligosaccharide of the gut symbiont Akkermansia muciniphila exhibits a remarkable structure and TLR signaling capacity. *Nat. Commun*. **15**, 8411 (2024).
23. Pradel, E., Parker, C. T. & Schnaitman, C. A. Structures of the *rfaB*, *rfaI*, *rfaJ*, and *rfaS* genes of *Escherichia coli* K-12 and their roles in assembly of the lipopolysaccharide core. *J. Bacteriol.* **174**, 4736–4745 (1992).
24. Porter, N.T. et al. Phase-variable capsular polysaccharides and lipoproteins modify bacteriophage susceptibility in Bacteroides thetaiotaomicron. *Nat. Microbiol.* **5**, 1170–1181 (2020).
25. Chatzidaki-Livanis, Maria et al. A family of transcriptional antitermination factors necessary for synthesis of the capsular polysaccharides of *Bacteroides fragilis*. al antitermination factors necessary for synthesis of the capsular polysaccharides of *Bacteroides fragilis*. *J. Bacteriol.* **191**, 7288–7295 (2009).
26. Chatzidaki-Livanis M, Weinacht K. G. & Comstock L. E. Trans locus inhibitors limit concomitant polysaccharide synthesis in the human gut symbiont *Bacteroides fragilis*. *Proc. Natl. Acad. Sci. U S A*. 107, 11976-11980 (2010).
27. Lehrer, J., Vigeant, K. A., Tatar, L. D. & Valvano, M. A. Functional characterization and membrane topology of *Escherichia coli* WecA, a sugar-phosphate transferase initiating the biosynthesis of enterobacterial common antigen and O-antigen lipopolysaccharide. *J. Bacteriol.* **189**, 2618–2628 (2007).
28. Gilbert, M. et al. The genetic bases for the variation in the lipo-oligosaccharide of the mucosal pathogen, *Campylobacter jejuni*: biosynthesis of sialylated ganglioside mimics in the core oligosaccharide. *J. Biol. Chem.* **277**, 327–337 (2002).
29. Kappler, K. et al. Increased antibody response to fucosylated oligosaccharides and fucose-carrying Bacteroides species in Crohn's disease. *Front. Microbiol*. **11**, 1553 (2020).
30. Kuwahara, T. et al. Genomic analysis of *Bacteroides fragilis* reveals extensive DNA inversions regulating cell surface adaptation. *Proc. Natl Acad. Sci. USA* **101**, 14919–14924 (2004).
31. Abramson, J. et al. Accurate structure prediction of biomolecular interactions with AlphaFold 3. *Nature* **630**, 493–500 (2024).
32. Holm, L. & Laakso, L. M. Dali server update. *Nucleic Acids Res*. **44**, W351–W355 (2016).
33. Bamford, N. C. et al. Structural and biochemical characterization of the exopolysaccharide deacetylase Agd3 required for Aspergillus fumigatus biofilm formation. *Nat. Commun.* **11**, 2450 (2020).
34. Little, D. J. et al. The structure- and metal-dependent activity of Escherichia coli PgaB provides insight into the partial de-N-acetylation of poly-β-1,6-N-acetyl-D-glucosamine. *J. Biol. Chem.* **287**, 31126-37 (2012).
35. Ghosh, M. K. & Raushel, F. M. Biosynthesis of UDP-α-N-acetyl-D-mannosaminuronic acid and CMP-β-N-acetyl-d-neuraminic acid for the capsular polysaccharides of *Campylobacter jejuni*. *Biochemistry* **63**, 688–698 (2024).
36. Whitfield, Chris. Biosynthesis and assembly of capsular polysaccharides in *Escherichia coli*. Annu. rev. biochem. v**75,** 39-68 (2006).
37. Wugeditsch, T. et al. Phosphorylation of Wzc, a tyrosine autokinase, is essential for assembly of group 1 capsular polysaccharides in Escherichia coli. *J. Biol. Chem.* **276**, 2361–2371 (2001).
38. Brigham, C. et al. Sialic acid (N-acetyl neuraminic acid) utilization by *Bacteroides fragilis* requires a novel N-acetyl mannosamine epimerase. *J.bacteriol.* **191**, 3629-3638 (2009).
39. McKee, L. S. et al. Polysaccharide degradation by the Bacteroidetes: mechanisms and nomenclature. *Environ. Microbiol. Rep*. **13**, 559-581 (2021).
40. Grondin, J. M., Tamura, K., Déjean, G., Abbott, D. W. & Brumer, H. Polysaccharide utilization loci: fueling microbial communities. *J. Bacteriol*. **199**, e00860-16 (2017).
41. Kijner, S., Ennis, D., Shmorak, S., Florentin, A. & Yassour, M. CRISPR-Cas-based identification of a sialylated human milk oligosaccharides utilization cluster in the infant gut commensal *Bacteroides dorei*. *Nat. Commun.* **15**, 105 (2024).
42. Brockhausen, I. Crossroads between Bacterial and Mammalian Glycosyltransferases. *Front. Immunol*. **5**, 492(2014).

43. Jöud, M., Möller, M. & Olsson, M.L. Identification of human glycosyltransferase genes expressed in erythroid cells predicts potential carbohydrate blood group loci. *Sci. Rep.* **8**, 6040 (2018).
44. Nakayama, J. et al. Expression cloning of a human α1,4-N-acetylglucosaminyltransferase that forms GlcNAcα1→4Galβ→R. *Proc. Natl Acad. Sci. USA* **96**, 8991–8996 (1999).
45. Ihara, H. et al. Crystal structure of mammalian α1,6-fucosyltransferase, FUT8. *Glycobiology* **17**, 455–466 (2007).
46. Duncan, S.H., Barcenilla, A., Stewart, C.S., Pryde, S.E. & Flint, H.J. *Appl. Environ. Microbiol.* **68**, 5186-5190 (2002).
47. Galanos, C., Lüderitz, O. & Westphal, O. *Eur. J. Biochem.* **9**, 245–249 (1969).
48. Hirschfeld, M., Ma, Y., Weis, J. H., Vogel, S. N. & Weis, J. J. Cutting edge: repurification of lipopolysaccharide eliminates signaling through both Toll-like receptor 2 and Toll-like receptor 4. *J. Immunol.* **165**, 618–622 (2000).
49. Alvarez, C. A. & Cobb, B. A. Purification of capsular polysaccharide complex from Gram-negative bacteria. *Methods Mol. Biol.* **1954**, 25–35 (2019).
50. Garcia-Vello, P., Speciale, I., Di Lorenzo, F., Molinaro, A. & De Castro, C. Structural analysis of lipopolysaccharides from Gram-negative bacteria. *Methods Mol. Biol.* **2548**, 181–209 (2022).
51. Leontein, K. Assignment of absolute configuration of sugars by g.l.c. of their acetylated glycosides formed from chiral alcohols. *Methods Carbohydr. Chem.* **1978**, *62*, 359–362.
52. Rietschel, E. T. Absolute Configuration of 3-Hydroxy Fatty Acids Present in Lipopolysaccharides from Various Bacterial Groups. *Eur. J. Biochem.* **64**, 423–428 (1976).
53. Stern, A. S., Li, K. B. & Hoch, J. C. Modern spectrum analysis in multidimensional NMR spectroscopy: comparison of linear-prediction extrapolation and maximum-entropy reconstruction. *J. Am. Chem. Soc.* **124**, 1982–1993 (2002).
54. Di Lorenzo, F. The lipopolysaccharide lipid A structure from the marine sponge-associated bacterium *Pseudoalteromonas* sp. 2A. *Antonie Van Leeuwenhoek* **110**, 1401–1412 (2017).
55. Larrouy-Maumus, G., Clements, A., Filloux, A., McCarthy, R. R. & Mostowy, S. Direct evidence of lipid A heterogeneity in clinical isolates of *Pseudomonas aeruginosa*. *J. Microbiol. Methods* **120**, 68–71 (2016).
56. Aramaki, T. et al. KofamKOALA: KEGG ortholog assignment based on profile HMM and adaptive score threshold. *Bioinformatics* **36**, 2251–2252 (2020).
57. Lam, J. S. et al. Genetic and functional diversity of *Pseudomonas aeruginosa* lipopolysaccharide. *Front. Microbiol.* **2**, 118 (2011).
58. Jacobson, A. N., Choudhury, B. P., & Fischbach, M. A.. The Biosynthesis of Lipooligosaccharide from *Bacteroides thetaiotaomicron*. *mBio*, **9**, e02289-17 (2018).
59. Zulkower, V. & Rosser, S. DNA Features Viewer: a sequence annotation formatting and plotting library for Python. *Bioinformatics* **36**, 4350–4352 (2020).
60. Camacho, C. et al. BLAST+: architecture and applications. *BMC Bioinformatics* **10**, 421 (2009).
61. Virtanen, P. et al. SciPy 1.0: fundamental algorithms for scientific computing in Python. *Nat. Methods* **17**, 261–272 (2020).
62. Waskom, M. seaborn: statistical data visualization. *J. Open Source Softw.* **6**, 3021 (2021).
63. Baxter, E. W. et al. Standardized protocols for differentiation of THP-1 cells to macrophages with distinct M(IFNγ+LPS), M(IL-4) and M(IL-10) phenotypes. *J. Immunol. Methods* **478**, 112721 (2020).
64. Daguet, D. et al. Arabinogalactan and fructooligosaccharides improve the gut barrier function in distinct areas of the colon in the Simulator of the Human Intestinal Microbial Ecosystem. *J. Funct. Foods* **20**, 369–379 (2016).
